# Feasible deployment of carbon capture and storage and the requirements of climate targets

**DOI:** 10.1038/s41558-024-02104-0

**Published:** 2024-09-25

**Authors:** Tsimafei Kazlou, Aleh Cherp, Jessica Jewell

**Affiliations:** 1 Centre for Climate and Energy Transformation (CET), University of Bergen, Bergen, Norway; 2 Department of Geography, Faculty of Social Sciences, University of Bergen, Bergen, Norway; 3 Department of Environmental Sciences and Policy, Central European University, Vienna, Austria; 4 International Institute for Industrial Environmental Economics, Lund University, Lund, Sweden; 5 Division of Physical Resource Theory, Department of Space, Earth and Environment, Chalmers University of Technology, Gothenburg, Sweden; 6 Advancing Systems Analysis Program, International Institute for Applied Systems Analysis, Laxeburg, Austria

**Keywords:** Climate-change mitigation, Energy modelling, Climate-change mitigation

## Abstract

Climate change mitigation requires the large-scale deployment of carbon capture and storage (CCS). Recent plans indicate an eight-fold increase in CCS capacity by 2030, yet the feasibility of CCS expansion is debated. Using historical growth of CCS and other policy-driven technologies, we show that if plans double between 2023 and 2025 and their failure rates decrease by half, CCS could reach 0.37 GtCO_2 _yr^−1^ by 2030—lower than most 1.5 °C pathways but higher than most 2 °C pathways. Staying on-track to 2 °C would require that in 2030–2040 CCS accelerates at least as fast as wind power did in the 2000s, and that after 2040, it grows faster than nuclear power did in the 1970s to 1980s. Only 10% of mitigation pathways meet these feasibility constraints, and virtually all of them depict <600 GtCO_2_ captured and stored by 2100. Relaxing the constraints by assuming no failures of CCS plans and growth as fast as flue-gas desulfurization would approximately double this amount.

## Abstract (web summary)

Carbon capture and storage is a key component of mitigation scenarios, yet its feasibility is debated. An analysis based on historical trends in policy-driven technologies, current plans and their failure rates shows that a number of 2 °C pathways are feasible, but most 1.5 °C pathways are not.

## Main

Carbon capture and storage (CCS) plays a key role in climate mitigation pathways, yet its feasibility is vigorously debated^1–3^. The recent interest in CCS^4–6^, including negative emissions technologies—direct air capture (DACCS) and bioenergy with CCS (BECCS)—is reflected in plans to increase CCS capacity eight-fold from 2023 to 2030^7^. However, 10 years ago, a similar wave of CCS plans^5^ largely failed^8,9^. Can the new push bring CCS on track^10–13^ for the Paris climate targets?

Answering this question requires overcoming three challenges. The first is anticipating how many CCS plans are likely to succeed. The second is projecting medium-term growth of CCS, given the uncertainty about the drivers of, and barriers to, its uptake^14,15^. The third is estimating feasible long-term growth rates that depend on the size of the future CCS market^16,17^.

We address these challenges by building on the tradition of using empirical evidence^18–26^ from historical technology analogues or reference cases^27,28^. Using advanced policy-driven technologies as reference cases, we contribute with three methodological innovations. First, we analyse historical failure rates of planned projects to estimate feasible near-term (5–10 years) CCS deployment. Second, we use this estimate to project a range of medium-term (10–20 years) CCS expansion, assuming quasi-exponential growth typical of early stages of technology deployment. Finally, we estimate the feasible range of long-term (20–80 years) CCS growth rates based on the peak growth rates of historical analogues. Thus, we develop an approach for projecting the deployment of emerging policy-driven technologies across the first three phases of the technology life-cycle—the formative phase^29–31^, the acceleration phase^19^ and the stable growth phase^19^. Finally, we compare our findings to CCS growth in the three recent IPCC scenario ensembles^32–34^ and estimate the feasible range of CO_2_ captured and stored with CCS over the 21st century.

We find that only a handful of climate mitigation pathways (10%, IPCC categories C1–C4) depict CCS capacity growth compatible with even the most optimistic assumptions when (1) CCS plans double by 2025 and their failure rate decreases by half; (2) CCS expansion in 2030–2040 is as fast as solar power expansion was in the 2010s or nuclear power expansion was in the 1960s and 1970s; and (3) CCS grows over the following decades as fast as the growth of nuclear in the 1970s and 1980s. Only 33% of pathways meet the first two constraints and only 26% meet the last one. Virtually all pathways that meet all three constraints depict <200 GtCO_2_ captured and stored by 2070 and <600 GtCO_2_ by 2100 (at the 95th percentile). Under the less realistic assumptions of a doubling of CCS plans by 2025, a zero failure rate and growth similar to that of flue-gas desulfurization (FGD), this amount could increase to 400 GtCO_2_ by 2070 and 1,100 GtCO_2_ by 2100, which still stands in contrast to a large number of 1.5 °C- and 2 °C-compatible pathways, which envision up to 700 GtCO_2_ captured and stored by 2070 and 1,400 GtCO_2_ by 2100.

## Growth phases of policy-driven emerging technologies

**Fig. 1:**
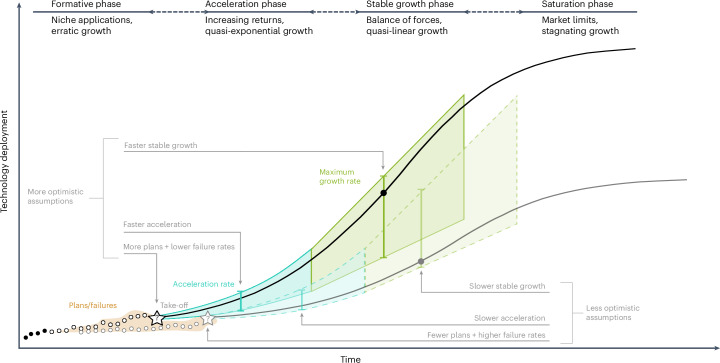
Method for projecting the feasible deployment of policy-driven technologies along the phases of technology growth using feasibility spaces. To construct each feasibility space, we use a tailored set of metrics and reference cases most appropriate to each of the first three phases of the technology life-cycle—formative, acceleration and stable growth (Methods and Table 1). For the formative phase, we project feasible CCS deployment (Gt yr^−1^) based on project plans and their failure rates; for the acceleration phase, the acceleration rate of reference technologies; and for the stable growth rate, we use the maximum growth rate at the inflection point of the S-curve normalized to the market size. This approach can be applied not only to global but also to national and regional targets, as well as to other climate mitigation and energy technologies. Error bars are used to illustrate the uncertainty in feasible deployment over time.

The growth of new technologies starts with a formative phase^30,31,35^, when “the technology is tested, refined and adapted to market conditions”^29^. At the end of the formative phase—typically between 0.1% and 2.5% of the final market^17,19,22,30,35^ (A. Jakhmola, J.J., V. Vinichenko and A.C., manuscript in preparation)—a technology ‘takes off’, driven by increasing returns^36^. This leads to an acceleration phase with quasi-exponential growth^19,21,36^. Eventually, countervailing forces such as resource availability and socio-political resistance dampen the acceleration in the stable growth phase, where the growth peaks and is no longer accelerating^19,22,37^, which is eventually followed by slow-down and saturation due to market limits^38^. Feasibility constraints in the different phases reflect different growth mechanisms, and therefore require separate assessments (Fig. 1). We use the feasibility space approach^27,39^, which compares a target case—in this case, future CCS growth—with reference cases^40,41^ appropriate for each growth phase (Table 1 and Methods).

**Table 1:** Reference cases and metrics used to construct feasibility spaces of CCS deployment in the formative, acceleration and stable growth phases

Phase	Target case	Reference cases	Metrics
**Formative**	CCS deployment by 2030	Historical CCS deployment (Fig. 2 and Supplementary Tables 1–5) Early nuclear power (United States, 1972–1982)^48^	Capacity planned to 2025 (Gt yr^−1^), failure rate of planned projects (%)
**Acceleration**	CCS growth 2030–2040	Nuclear power (global) at the acceleration phase^75^ Wind power (global) at the acceleration phase^76^ Solar power (global) at the acceleration phase^77^ FGD (global)^18,70^^,a^	CAGR (%) over 10 years since reaching the same market penetration as in the target case
**Stable growth**	CCS capacity peak annual additions	Nuclear power (global, regional and national)^55,76^ Wind power (global, regional and national)^19,55^ Solar power (global, regional and national)^19,55^ FGD (global)^18,70^^,a^	Maximum growth rate (*G*) or the most recent 3-year growth rate (*R*_3_) in cases where deployment is before the inflection point (Gt yr^−2^) ^19,55^. In both cases, normalized to the market size (%)

See Methods for additional discussion of reference cases.

^a^In this study, the use of FGD was twofold—as a reference case for the CO_2_ capture component of CCS and as the most optimistic (albeit less realistic) reference case for CCS as a whole in the sensitivity analysis (Methods and Supplementary Note 2).

For the formative phase, which we find can be complete by 2030, we construct a feasibility space based on near-term CCS plans and historical failure rates of past CCS (Supplementary Note 1) and early nuclear power plans. For the acceleration phase, which we assume would occur in 2030–2040, we construct a feasibility space using the compound annual growth rate (CAGR) metric derived from reference cases of historical nuclear, solar and wind power growth at similar levels of market penetration. For the stable growth phase in the post-2040 period, we construct a feasibility space using maximum annual capacity additions normalized to the market size^18,19^ derived from reference cases of nuclear, wind and solar power growth at the global, regional and national levels. Subsequently, we map the 1.5 °C- and 2 °C-compatible IPCC Sixth Assessment Report (AR6) pathways^10,32^ (Methods) onto the three feasibility spaces and calculate the amount of CO_2_ captured and stored in the pathways that meet the feasibility constraints. Finally, we use FGD, which is similar to the CO_2_ capture component of CCS, as a reference case for a sensitivity analysis representing the most optimistic (albeit relatively unrealistic) assumptions about CCS growth (Supplementary Note 2).

## More CCS plans and fewer failures needed for climate targets

**Fig. 2:**
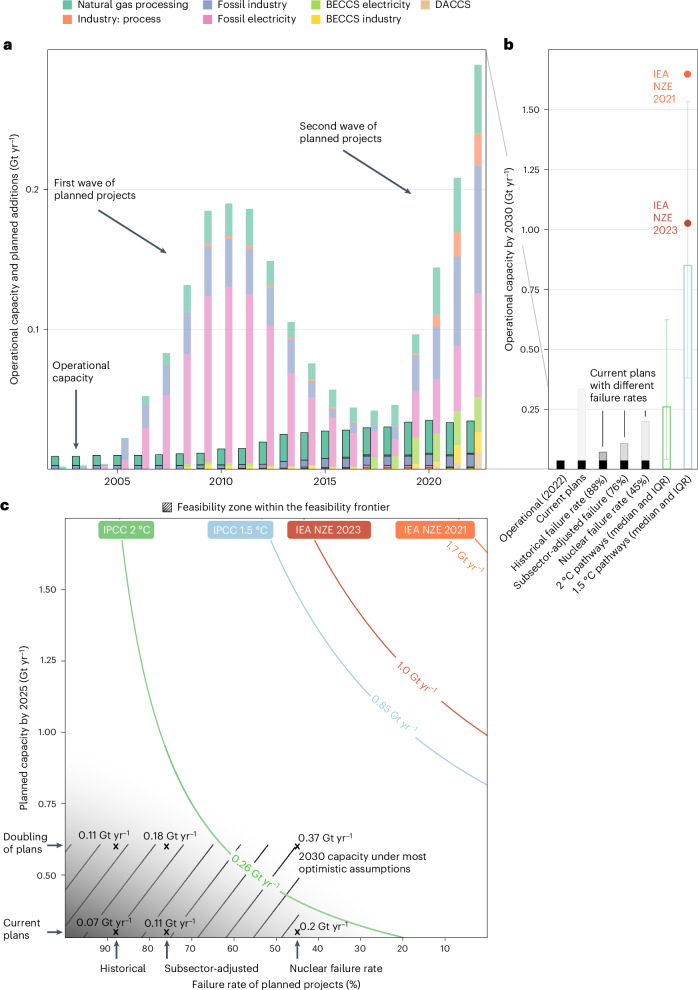
Historical development and prospects for near-term CCS deployment. **a**, Operational (dark) and planned (light) CCS capacity in 2002–2022, by sector^43^ (Methods). **b**, Operational capacity in 2030 based on current operational capacity (black bars) and current (2022) plans under different failure rates (grey bars) compared to the IPCC AR6 1.5 °C- (*n* = 218) and 2 °C-compatible (*n* = 423) pathways (coloured bars illustrate the medians; error bars show the IQR^32^) and recent IEA NZE pathways (coloured dots)^12,13^. **c**, Feasibility space of CCS deployment in the formative phase depicting operational CCS capacity (Gt yr^−1^) (Supplementary Table 6) in 2030 as a function of CCS plans (*y* axis) and their failure rates (*x* axis). The hatched zone represents all observations within the feasibility frontier and thus those consistent with empirically grounded assumptions about near-term CCS plans and failure rates (crosses, Table 1). The shading shows that this frontier is fuzzy or, in other words, not binary^27,39^. The isolines show different combinations of planned capacity and failure rates that lead to the same operational CCS capacity in 2030. The IEA NZE pathways^12,13^ are indicated by red and orange isolines, and the median CCS capacity in the IPCC AR6 pathways^32^ are shown with blue (1.5 °C-compatible) and green (2 °C-compatible) isolines. Source data

**Extended Data Fig. 1:**
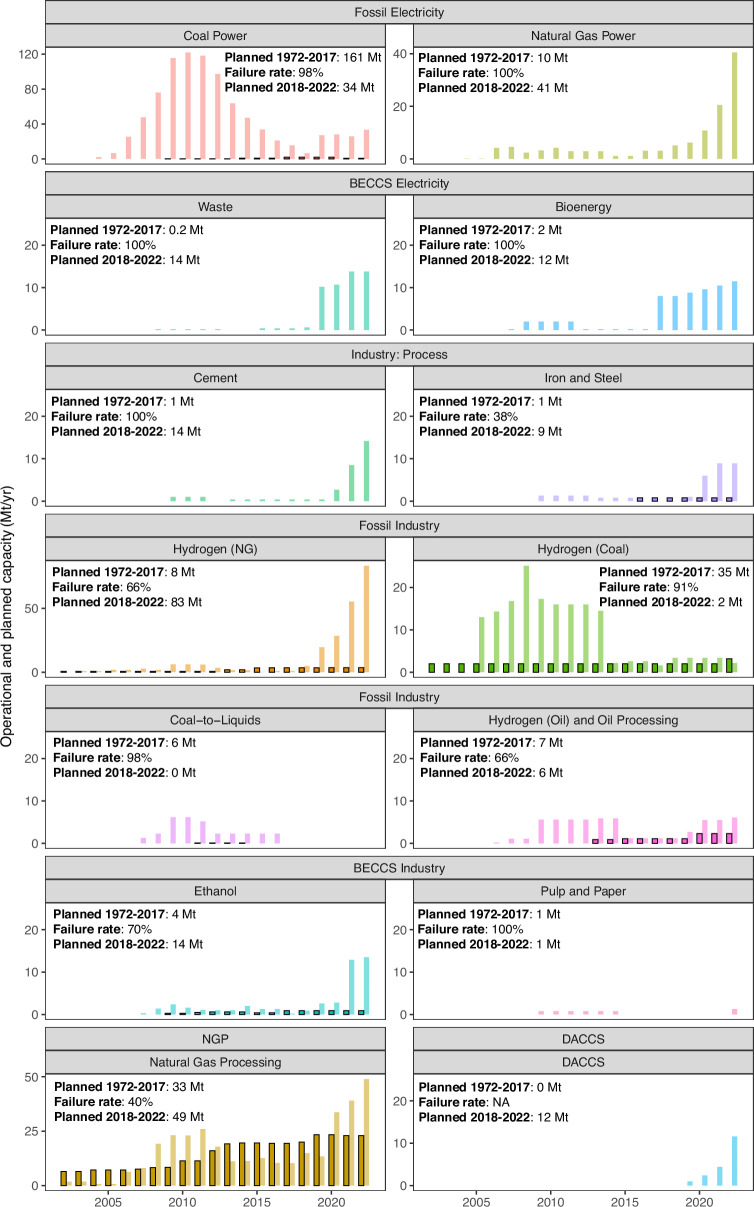
Planned and operational CCS capacity, grouped by sector and subsector. Each facet shows operational (dark colored bars) and planned (light colored bars) CCS capacity in 2002-2022, in Mt/yr. Planned capacity and historical failure rates (1972-2017) for each subsectoral application of CCS are reported in text on each panel.

Recent reports indicate that if all current project plans are realized, operational CCS capacity would reach 0.34 Gt yr^−1^ by 2030^5,7^. However, we have seen ambitious plans before; the initially promising first wave of CCS plans failed to meet expectations despite a number of supportive policies^8,9,42^ (Fig. 2 and Extended Data Fig. 1). If all plans from the first wave had been realized, today’s operational capacity would be around 0.27 Gt yr^−1^ (Supplementary Table 1), instead of the paltry 0.04 Gt yr^−1^ operational today.

The CCS capacity installed by 2030 depends on the plans announced by 2025 because CCS projects take, on average, 5 years from announcement to completion. Under an optimistic assumption that the planned capacity will double between 2022 and 2025, the 2030 capacity could reach 0.6 Gt yr^−1^ (Methods). However, how much CCS will be actually installed also depends on the failure rate—the share of planned capacity that is not realized. To determine a realistic range of failure rates, we first calculate the historical failure rate of CCS projects from 1972 to 2018 (88%)^43^ (Supplementary Tables 1–4 and Methods). This industry-wide failure rate varies across sectors and subsectors^44–47^, with CCS plans in the first wave dominated by the electricity sector, which failed at a rate of over 90%. In the current wave, the planned projects are more diversified (Fig. 2). Based on historical subsector-specific failure rates, we estimate that the aggregate failure rate of today’s plans would drop to 76% (Methods). Finally, we measure the failure rate of early nuclear power plans in the United States (45%), which was a policy-driven technology developed in the wake of the oil crises of the 1970s^48–50^. At the start of this expansion, nuclear power contributed 2% of global and 3% of US electricity markets, which was more advanced than CCS is today, yet still close to the formative phase. Given the capital intensity^51^, lumpiness^52^ and complexity^53,54^ of nuclear power, we consider it as a close technological analogue of CCS and use it as an optimistic reference case.

With the capacity announced by 2022 and the historical failure rate (88%), CCS capacity in 2030 would be 0.07 Gt yr^−1^. If the planned capacity doubles between 2023 and 2025 and the failure rate drops to that of nuclear power in the United States (45%), CCS capacity would reach 0.37 Gt yr^−1^ by 2030. We consider this the upper feasible bound. By contrast, the IPCC AR6 1.5 °C pathways^10,32^ envisage a median CCS capacity of 0.9 Gt yr^−1^ (interquartile range (IQR) 0.4–1.5) by 2030. The International Energy Agency (IEA) net-zero emissions (NZE) scenario^13^ envisages an even higher 1 Gt yr^−1^ capacity (1.7 Gt yr^−1^ in the 2021 edition^12^), which is almost definitely out of reach given the current project timeline and likely failure rates. However, the 2 °C pathways envision a median CCS capacity of 0.3 Gt yr^−1^ (IQR 0.04–0.6), which could be achieved by expanding the planned capacity to 0.4–0.6 Gt yr^−1^ by 2025 and reducing the failure rate to 45–60% (Fig. 2).

## CCS growth should accelerate at least as fast as wind power

As we show, it is realistic for CCS capacity in 2030 to reach 0.07–0.37 Gt yr^−1^ capacity. This would amount to 0.3–1.8% of the market potential, or all CO_2_ emissions in sectors where CCS plans have been announced (Methods), thus resulting in the take-off and subsequent quasi-exponential growth characteristic of the acceleration phase. Can such growth bring CCS on track for climate targets by 2040? To answer this question, we project a range of feasible 2040 CCS capacity values based on: (1) the feasible range of the 2030 capacity estimated in the previous section; and (2) feasible year-on-year growth rates in the acceleration phase in 2030–2040, derived from the reference cases of three policy-driven technologies—nuclear, wind and solar power—at similar levels of market penetration (Methods).

**Fig. 3:**
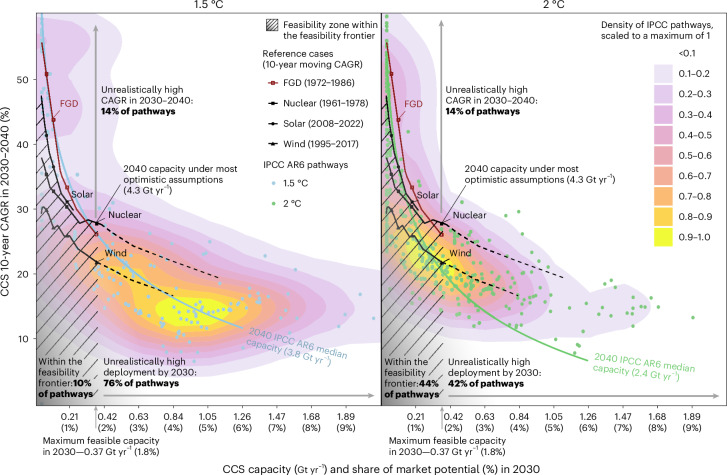
Feasibility space of CCS deployment for the acceleration phase in 2030–2040 compared to IPCC AR6 pathways. CCS capacity and market penetration achieved by 2030 (*x* axis) versus a 10-year moving CAGR in 2030–2040 (*y* axis). The maximum feasible capacity in 2030 makes up the feasibility frontier along the *x* axis (0.37 Gt yr^−^^1^ or 1.8% of the market potential) (Fig. 2c). Acceleration rates for the reference cases make up the three feasibility frontiers for CCS acceleration in 2030–2040, with the black lines showing the historical acceleration rates of nuclear (1961–1978), wind (1995–2017) and solar (2008–2022) power as reference cases for the CCS (Table 1). Dashed lines illustrate the continuation of these reference cases under higher than realistic CCS capacity by 2030. The dark red line shows the historical acceleration of FGD (1972–1986) as a reference case for the capture component of CO_2_. The hatched zone represents all observations within the feasibility frontier and the shading shows that this frontier is fuzzy, or in other words not binary^27,39^. The 1.5 °C- and 2 °C-compatible pathways^10,32^ are shown as dots and their distributions form the two-dimensional (2D) density plot (from white to yellow). Blue and green isolines show different combinations of the two metrics that lead to the median CCS capacity in the 1.5 °C- and 2 °C-compatible pathways, respectively (Table 2), regardless of feasibility considerations. The *x* axis of this figure is cut off at 2.1 Gt yr^−1^ (10% market penetration), thus excluding 47 1.5 °C pathways (20%) and 33 2 °C pathways (8%) with a CCS capacity of up to 21 Gt yr^−1^ by 2030. Density plots are constructed from the entire sample of pathways (*n* = 218 for 1.5 °C, *n* = 423 for 2 °C). Source data

We estimate the range of CCS capacity achievable by 2040 to be around 0.95–4.3 Gt yr^−1^ (Table 2). The upper end of this range can only be reached under optimistic assumptions about CCS deployment in the formative phase (0.37 Gt yr^−1^) and acceleration in 2030–2040 comparable to that of nuclear power in the 1970s (Fig. 3 and Supplementary Fig. 1), or if CCS accelerates as fast as FGD did in the 1980s. Under these optimistic assumptions, CCS capacity in 2040 would be above the median of the 1.5 °C pathways (3.8 Gt yr^−1^). The median of the 2 °C pathways (2.4 Gt yr^−1^) could be reached under more modest, albeit still optimistic, assumptions about CCS deployment in the formative phase (0.26–0.37 Gt yr^−1^) and with acceleration comparable to that of wind power in the 2000s (Table 2 and Fig. 3).

**Table 2:** Feasible upper bounds of CCS capacity in 2030 and 2040 estimated from reference cases and compared to the IPCC AR6 pathways

Formative phase (pre-2030) assumptions	Current plans and 88% failure	Current plans and 76% failure	Current plans and 45% failure	Plans doubling and 45% failure
CCS capacity in 2030	0.07	0.11	0.2	0.37
**IPCC AR6 capacity in 2030**	2 °C: 0.3 [0.04–0.6]		1.5 °C: 0.9 [0.4–1.5]	
**Acceleration in 2030–2040**	same as wind			
CAGR for reference period CCS capacity in 2040	30% 0.95	27% 1.2	26% 2.0	22% 2.6
	same as nuclear			
CAGR for reference period CCS capacity in 2040	35% 1.4	33% 1.9	30% 2.8	28% 4.3
	same as solar			
CAGR for reference period CCS capacity in 2040	41% 2.2	37% 2.5	31% 3.0	NA NA
**IPCC AR6 capacity in 2040**	2 °C: 2.4 [1.6–3.9]		1.5 °C: 3.8 [2.5–7.1]	

The columns illustrate the CCS capacity in 2030 based on different assumptions of near-term CCS plans and failure rates (Fig. 2). The rows illustrate 10-year CAGRs for each reference case corresponding to a comparable level of market penetration and the resulting CCS capacity by 2040. Values are in GtCO_2_ yr^−1^ unless otherwise indicated. The reference case growth rates in the acceleration phase reported in this table are also illustrated in Fig. 3. Solar power started accelerating recently, so it is not possible to measure its CAGR for the most optimistic outcome of the formative phase. IPCC pathways are in bold and the IQRs of CCS capacity in the IPCC pathways are indicated in square brackets. See Methods and Table 1 for details of reference cases.

Despite being in line with the realistic range of 2040 CCS capacity, most pathways fall outside the feasibility frontier when we account for the timing of acceleration (Fig. 3). A total of 76% of 1.5 °C- and 42% of 2 °C-compatible pathways depict unrealistically fast growth by 2030, with an additional 14% of both 1.5 °C- and 2 °C-compatible pathways requiring unrealistically fast acceleration from 2030–2040. Only 10% of 1.5 °C- and 44% of 2 °C-compatible pathways are located within the feasibility frontier for the formative and acceleration phases. Even under a less realistic assumption that the growth of CCS will accelerate as fast as for FGD, only 18% of the 1.5 °C-compatible pathways would be within the feasibility frontier.

## In most pathways CCS grows faster than nuclear at its peak

After acceleration, technologies enter a stable growth phase when annual additions peak at the maximum growth rate (*G*) at the inflection point of the S-curve^19^ (Fig. 1). We find that the median values of the maximum annual additions of CCS capacity were similar (0.4–0.5 Gt yr^−1^ added annually) across the 1.5 °C-, 2 °C- and 2.5 °C-compatible pathways (Supplementary Figs. 2 and 3). What varies is *when* the fastest growth is achieved: in the 1.5 °C-compatible pathways, it occurs around 2045, in the 2 °C-compatible pathways around 2055 and in the 2.5 °C-compatible pathways around 2065.

We use policy-driven low-carbon technologies—nuclear, wind and solar power—as reference cases for growth rates at the stable growth phase. We complement global observations with regional and national observations where the growth of these technologies had already peaked (Extended Data Table 1)^19,55^. To compare maximum annual capacity additions across reference cases and mitigation pathways, we normalize *G* to the size of the market—the total electricity supply for the reference cases, and the sum of the gross CO_2_ emissions in sectors with capturable emissions plus negative emissions from BECCS and DACCS for CCS (Methods).

**Extended Data Fig. 2:**
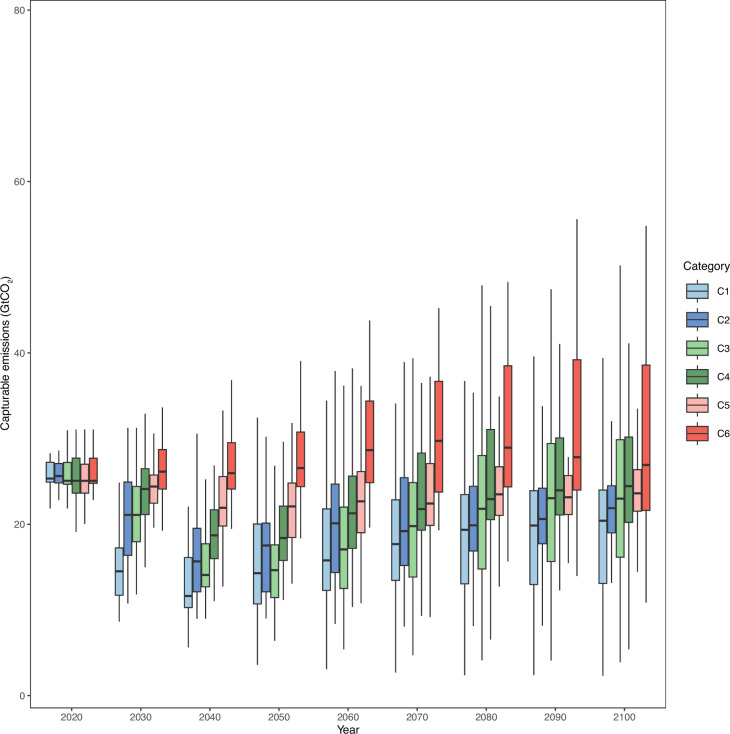
Capturable emissions in the IPCC AR6 scenario ensemble. This figure displays the size of potential CCS market over time (M_t_), in GtCO_2_ (Methods). The box-plots show the interquartile range (IQR) with the median marked with a black line and whiskers extending from the IQR range to nonoutlier minimum and maximum. Categories vary by global mean surface air temperature change by 2100: C1 pathways stay below 1.5°C with no or limited overshoot with a 50% probability (n=81); C2 - below 1.5°C with high overshoot (n=111); C3 - likely below 2°C (n=249); C4 - below 2°C (n=132); C5 - below 2.5°C (n=188); C6 - below 3.0°C (n=81)^10^. 9% of pathways (86 out of 928 across all categories; 68 of them are C1-C4) failed to produce this metric due to missing variables. Source data

**Extended Data Fig. 3:**
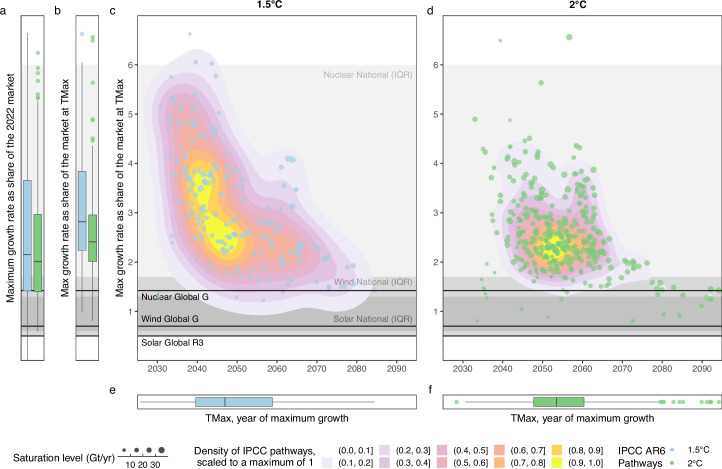
Maximum growth rates of CCS capacity in the stable growth phase in 1.5°C- and 2°C-compatible IPCC AR6 pathways, compared to maximum growth rates of nuclear, wind and solar power. a, The range of maximum annual growth rates normalised to the market size in 2022 in IPCC AR6 pathways^10,32^ (G_2_022, n=218 for 1.5°C and n=418 for 2°C; 5 pathways envision maximum growth after 2100 and thus are excluded from this analysis). b, The range of maximum annual growth rates normalised to the market size the year it is achieved (G_*TMax*_, n=568; 68 scenarios failed to produce *M*_*t*_ due to missing variables, see Extended Data Fig. 2 and Methods). c and d, The distribution of maximum growth rates (G_*TMax*_) and the years when it is achieved (TMax). 1.5°C- and 2°C-compatible pathways (n=192 for 1.5°C - panel c and n=376 for 2°C - panel d) shown as dots and their distribution forms the 2D-density plot (from white to yellow). The diameter of circles illustrates the saturation level of CCS capacity (Gt/yr) modelled for each pathway. e and f, The year of maximum annual growth (TMax, n=218 for 1.5°C and n=418 for 2°C). Each growth parameter is calculated as the average between logistic and Gompertz growth model outputs (Methods). In panels a, b, e, f, the box-plots show the interquartile range (IQR) of growth parameters in the pathway sample with the median marked with a black line and whiskers extending from the IQR range to nonoutlier minimum and maximum. In panels a-d, grey horizontal zones illustrate interquartile (IQR) ranges of G_*TMax*_ in reference cases at the national scale (n=20 for nuclear, n=35 for wind, n=24 for solar power), whereas the solid black lines illustrate reference cases of empirically observed G_*TMax*_ of wind and nuclear and R3 of solar power deployment globally^55^. Note: this figure does not display 16 outliers (2% of the sample) where G_*TMax*_ or G_2_022>7%, or 2095 > TMax < 2100 - these observations are still included in the calculation of density distribution in panels c, d and interquartile ranges in panels a, b. Source data

In most IPCC pathways, the use of fossil fuels declines earlier than the expansion of negative emissions, which means the potential CCS market may decline over time (Supplementary Fig. 4 and Extended Data Fig. 2). To account for market uncertainties, we normalize the CCS growth rates to both the maximum market size in 2022 (*G*_2022_) and to the market size at the time when the maximum growth rate was achieved (*G*_TMax_). Under both normalizations, the CCS growth in most 1.5 °C and 2 °C pathways is faster than the historical growth of nuclear, wind and solar globally. Under normalization to the 2022 market (*G*_2022_), only 26% of the 1.5 °C and 2 °C pathways depict global CCS growth consistent with the peak growth of global nuclear power (and ≤1% with the global growth of solar or wind). Under normalization to the future market (*G*_TMax_), only 5% of the 1.5 °C and 2 °C pathways depict global CCS growth consistent with the peak growth of global nuclear power (Extended Data Table 1 and Extended Data Fig. 3).

## In feasible pathways <600 GtCO_2_ is captured by 2100

**Fig. 4:**
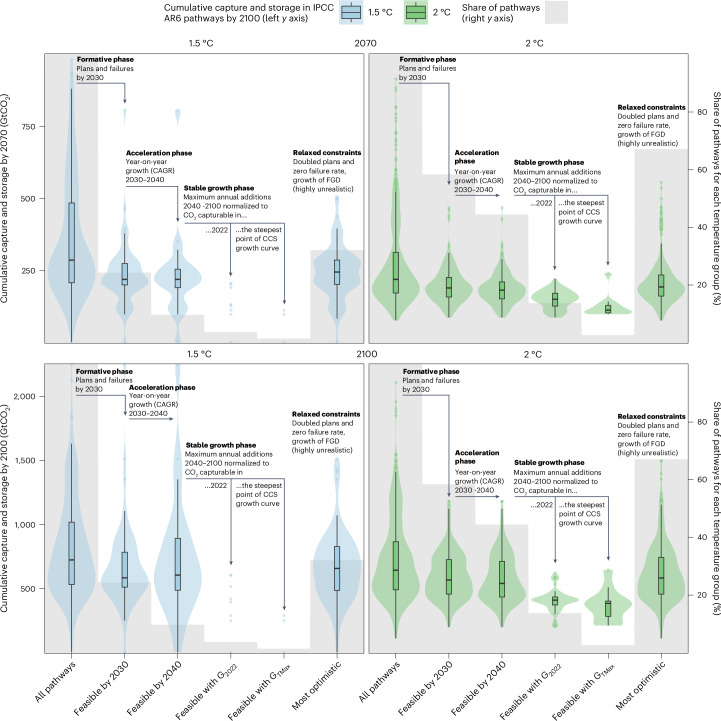
The effect of feasibility constraints on the long-term cumulative capture and storage of CO_2_ in 2030–2070 and 2030–2100 in IPCC AR6 pathways. The *x* axis contains different groups of mitigation pathways before and after imposing feasibility constraints, including feasible by 2030 under realistic project plans and failure rates, feasible by 2040 under acceleration rates similar to those of the reference cases, and feasible in the long term under maximum annual growth similar to the growth of the reference cases when normalized to capturable emissions today (*G*_2022_) and capturable emissions when the maximum growth is achieved (*G*_TMax_). ‘Most optimistic’ illustrates a group of pathways with relaxed constraints—0% failure rate and a doubling of plans, acceleration and stable growth (*G*_2022_) of FGD (Extended Data Fig. 4 and Supplementary Table 7). ‘All pathways’ is *n* = 218 for 1.5 °C in the left-hand panels and *n* = 423 for the 2 °C pathways in the right-hand panels. Violins and boxplots illustrate the cumulative CO_2_ capture and storage by 2070 and 2100 (in Gt) in these groups of pathways (left-hand *y* axis)—boxplots show the IQR, with the median marked by a black line and whiskers extending from the IQR range to the non-outlier minimum and maximum. Grey bars illustrate the share of pathways in each group (right-hand *y* axis). In groups with fewer than 10 pathways, we use dots rather than violins and boxplots. Source data

Only 10% of climate mitigation pathways (14% of the 2 °C- and 4% of the 1.5 °C-compatible pathways) meet all the feasibility constraints for CCS growth, and those that do capture and store considerably less CO_2_ over the 21st century. In fact, vetting the IPCC AR6 mitigation pathways for the feasibility of CCS growth reduces the upper bound of CO_2_ captured and stored by 2070 from 685 Gt to 201 Gt (both at the 95th percentile) (Supplementary Fig. 5), and by 2100 from 1,428 Gt to 589 Gt (both at the 95th percentile), even if the more relaxed long-term growth metric (*G*_2022_) is used (Fig. 4 and Extended Data Table 2). The effect is especially pronounced for the most stringent 1.5 °C-compatible pathways with no or limited overshoot (IPCC category C1), which typically rely on the early and rapid growth of CCS, and within which only the low-energy demand pathway (ref. ^56^) without any CCS satisfies all the CCS feasibility constraints.

**Extended Data Fig. 4:**
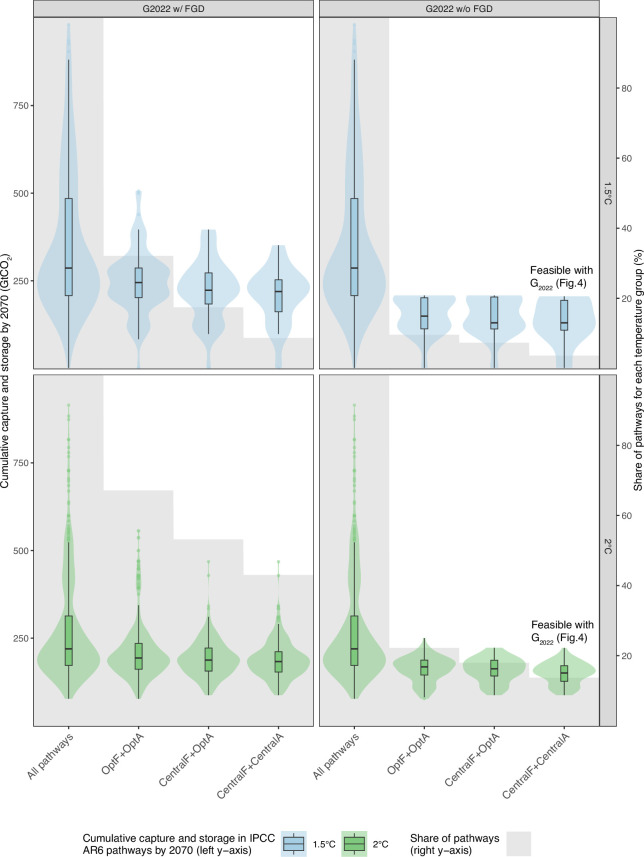
Sensitivity of long-term outcomes of CCS deployment to the most optimistic constraints in the three phases of growth. The figure illustrates how different combinations of the most optimistic constraints in the formative, acceleration (x-axis), and stable growth (columns) phases affect the cumulative CO_2_ capture and storage between 2030 and 2070 (left y-axis) and the share of IPCC pathways that meet these constraints (right y-axis). On the x-axis, ‘All pathways’ depicts 1.5°C- and 2°C-compatible (n=641, Methods) IPCC AR6 ensemble^32^, ‘OptF’ is the most optimistic constraint in the formative phase (0% failure rate and plans doubling between 2023 and 2025), ‘OptA’ is the most optimistic constraint in the acceleration phase (acceleration rate of FGD, Fig. 3), ‘CentralF’ and ‘CentralA’ are the formative and acceleration phase constraints used throughout this study (Table 1, Fig. 4, Methods). As for the stable growth phase, the right column (‘G2022 w/o FGD’) of the figure depicts the outcomes with the stable growth constraint used throughout this study (global nuclear power deployment), whereas the left column (‘G2022 w/ FGD’) depicts the outcomes with the stable growth constraint derived from the historical deployment of FGD (^18,70^, Extended Data Table 1). Violins and boxplots illustrate cumulative CO_2_ capture and storage between 2030 and 2070 (in Gt, left y-axis): the box-plots show the interquartile range (IQR) with the median marked with a black line and whiskers extending from the IQR range to nonoutlier minimum and maximum. Grey bars illustrate the share of pathways in each group (right y-axis). Source data

We test the sensitivity of these findings under 36 combinations of CCS plans, failure rates, near-term growth acceleration and long-term maximum growth rates, constrained at different levels (Supplementary Tables 7 and 8). The cumulative CO_2_ captured in IPCC pathways under the vast majority (95th percentile) of these sensitivity runs does not exceed 290 Gt by 2070 or 1,000 Gt by 2100. Out of all 36 sensitivity runs, the most optimistic one is that CCS plans double, their failure rate drops to zero and, subsequently, CCS deployment accelerates and grows as fast as FGD. Even in this case, only about half of the Paris-compatible pathways meet the constraints, with a median capture of 200 Gt (95th percentile at 398 Gt) in 2030–2070 and 592 Gt (95th percentile at 1,078 Gt), which is still considerably lower than in the full suite of AR6 pathways (95th percentile at 685 and 1,428 Gt, respectively) (Fig. 4 and Extended Data Fig. 4). We consider these relaxed constraints to be highly unrealistic, given the historical failure rates and because CCS is a more complex technology than FGD, with higher capital costs and larger infrastructure requirements, such as pipelines and storage, that potentially face more public opposition (Supplementary Note 2 and Supplementary Table 9).

## Discussion and conclusions

To delineate feasible trajectories of future CCS deployment, we develop an analytical approach based on technology diffusion theories and empirical evidence from CCS history and other policy-driven technologies. This study advances recent efforts to use empirical evidence for assessing the feasibility of low-carbon technologies^15,18,19,21,57,58^ through developing specific feasibility assessments for three separate phases of technology diffusion—formative, acceleration and stable growth—each with distinct policy challenges.

The first challenge is to increase the number of planned CCS projects while reducing their failure rates for the technology to take off. We show how realistic assumptions about failure rates, based on the history of CCS and other historical benchmarks, can identify a feasible upper bound of CCS capacity in 2030 (0.37 Gt yr^−1^). This analytical approach could be used for projecting the formative phase deployment of other policy-driven technologies, but more research is needed on failure rates and their dynamics.

The second challenge is to rapidly accelerate CCS after it has taken off, which we assume will start by 2030. We use the year-on-year growth rates of solar, wind and nuclear power as reference cases to define the upper bound for CCS capacity by 2040 (4.3 Gt yr^−1^). The literature is not consistent on how to use year-on-year growth rates in projecting technology diffusion, sometimes deriving them from formative phase data with erratic growth. We argue that year-on-year growth rates are most meaningful when derived from post-take-off reference cases with similar deployment levels and in conjunction with realistic estimates of initial deployment (in our case, CCS capacity in 2030). Policy support at this stage will require a strong emphasis on creating a market for CCS, either through taxing or regulating CO_2_ emissions (Supplementary Notes 3 and 4).

The final challenge is maintaining long-term CCS growth after its likely stabilization beyond 2040. Most 1.5 °C- and 2 °C-compatible pathways depict CCS capacity additions that are faster than the global growth of nuclear power and recent growth rates of wind and solar power. Nuclear power grew in the context of a rapidly expanding electricity demand and the oil crises of the 1970s^50,59,60^, while Europe’s renewable energy growth is also partially in response to an energy crisis^55^. Thus, nuclear and renewables may be too optimistic as reference cases for CCS, which is not being driven by an energy crisis.

**Extended Data Fig. 5:**
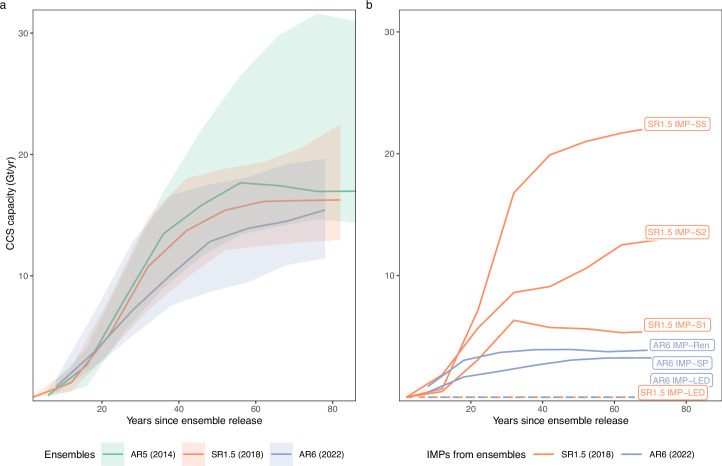
Projections of CCS capacity (incl. BECCS and DACCS) required to meet the most stringent climate targets in different IPCC scenario ensembles, in the years from the release of each ensemble. a, Median and interquartile range of CCS capacity in the three latest IPCC scenario ensembles for reaching below 1.5°C (Gt/yr). AR5^33^ sample includes Category 1 (430-480 ppm) which combines 1.5°C and (some) 2°C outcomes together^95^; SR1.5^34,96^ includes ’1.5C low overshoot’, ’1.5C high overshoot’, and ’Below 1.5C’ categories; AR6^10,32^ includes Category 1 and 2. b, 1.5°C Illustrative Mitigation Pathways (IMPs) in the IPCC SR1.5 and AR6 scenario ensembles. IMPs were used in SR1.5 (orange) and AR6 (blue). See Supplementary Table 10 for tabular illustration of this data. Source data

Our findings contribute to the debate about the potential role of CCS in climate mitigation^1,2,61–63^. The integrated assessment modelling community has addressed concerns about the outsized role of CCS in mitigation pathways^2,62^ by developing scenarios, including illustrative mitigation pathways, with low or no (BE)CCS^10,34,56,64–67^. At least three of the illustrative mitigation pathways (low demand, shifting pathways and gradual strengthening) from the recent IPCC AR6 report meet, or nearly meet, our feasibility constraints by relying on alternative mitigation strategies, such as suppressing demand, changing diets or shifting strong climate action towards the end of the century. In general, more recent pathways feature a somewhat less ambitious use of CCS (Extended Data Fig. 5, Supplementary Note 5 and Supplementary Table 10). The efforts to depict more-realistic CCS deployment in mitigation pathways has been supported by a strategy of vetting scenarios^68,69^ using thresholds for the maximum CO_2_ storage^68^ or decadal growth in electricity generation with CCS^69^.

Our more granular analysis identifies three additional constraints on CCS deployment—reaching take-off by 2030, achieving rapid acceleration in the 2030s and ensuring sustained long-term growth. Our findings are more conservative than those of previous studies^18,70,71^ because we consider potential failures of planned CCS projects and use reference cases that are more similar to CCS in terms of their complexity, capital-intensity (Supplementary Note 3), land use and public acceptance (Supplementary Note 2) than FGD^18^. We show that 10% of the 1.5 °C- and 44% of the 2 °C-compatible pathways depict realistic CCS capacity in 2040, which strongly correlates with the amount of CO_2_ cumulatively captured and stored in the long term (Supplementary Fig. 6). The additional constraints on long-term growth further reduce the number of feasible pathways to 1–4% of the 1.5 °C and 3–14% of the 2 °C pathways (depending on the assumptions about the size of the future market) (Fig. 4, Supplementary Note 6, Supplementary Figs. 7–11 and Supplementary Table 11) and reduce the (non-outlier) maximum amount of CO_2_ that can feasibly be captured and stored by 2100 to <600 GtCO_2_ (Fig. 4). This has significant implications for global carbon budgets.

Among the many applications of CCS, BECCS and DACCS have attracted a lot of attention because they are both essential for achieving negative emissions and thus climate targets^70,72–74^. Ref. ^73^ shows that BECCS and DACCS should complete the formative phase by 2040 to keep net-zero goals within reach. However, BECCS and DACCS together only account for 10% of current CCS plans^72^ (Fig. 2 and Supplementary Table 1), and while their development is important, it is the success of the remaining 90% of CCS projects that will be pivotal in enabling negative emissions technologies that would rely on capture, transportation and long-term storage developed for other CCS applications. Our results illustrate the challenges of ensuring the take-off and expansion of this wider and more diverse technological ecosystem that will be key to the success of BECCS and DACCS.

**Extended Data Fig. 6:**
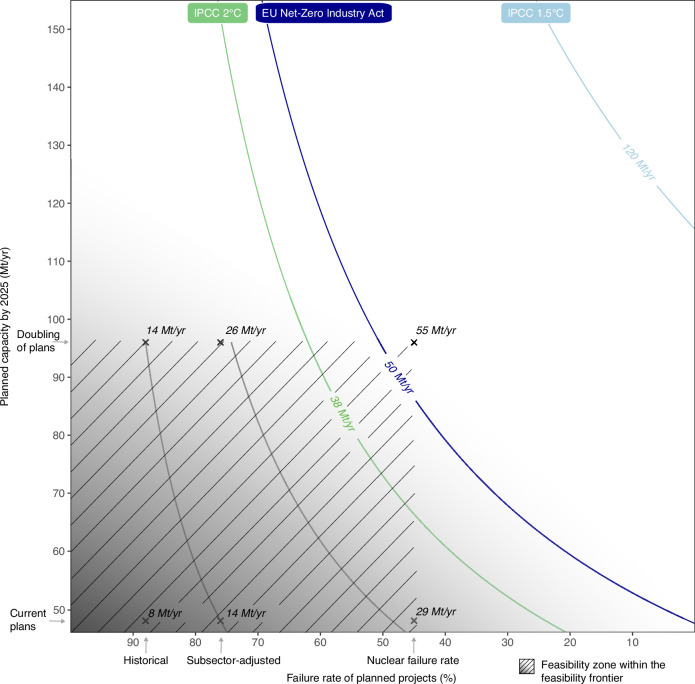
The EU Net Zero Industry Act CCS target and the feasibility space of CCS deployment at the formative phase in the EU by 2030. Feasibility space of CCS deployment in the EU depicting operational CCS capacity (Mt/yr) in 2030 as a function of CCS plans (y-axis) and their failure rates (x-axis). The hatched zone represents all observations within the feasibility frontier and thus consistent with empirically-grounded assumptions about near-term CCS plans and failure rates (crosses, Table 1). Isolines show different combinations of planned capacity and failure rates that lead to the same operational CCS capacity in 2030. The shading shows that this frontier is fuzzy^27,39^. Coloured isolines show median IPCC AR6 1.5°C and 2°C CCS capacity for R10EUROPE (which includes the United Kingdom) in 2030 as well as the EU Net-Zero Industry Act (NZIA) target (50 Mt^97^, Supplementary Note 7). Source data

**Extended Data Fig. 7:**
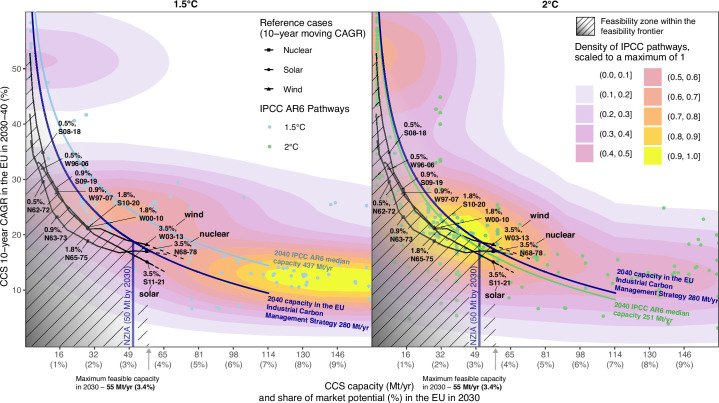
Feasibility space of CCS deployment in the EU for the acceleration phase in 2030-2040 compared to IPCC AR6 mitigation pathways. CCS capacity and market penetration achieved by 2030 (x-axis) in R10EUROPE region versus a 10-year moving compound annual growth rate (CAGR) in 2030-2040 (y-axis). 1.5°C- and 2°C-compatible scenarios^10,32^ shown as dots and their distribution forms the 2D-density plot (from white to yellow). The maximum feasible capacity in 2030 makes up the feasibility frontier along the x-axis (55 Mt/yr or 3.4% of the market potential, Fig. 2). Acceleration for reference cases make up the three feasibility frontiers (black lines) for CCS acceleration in 2030-2040 based on the historical acceleration rates of nuclear, wind, and solar power in EU28+UK as reference cases for CCS (Table 1). Dashed lines illustrate the continuation of these reference cases under higher than realistic CCS capacity by 2030. The hatched zone represents all observations within the feasibility frontier and the shading shows that this frontier is fuzzy^27,39^. For other (non-EU) reference cases, see Extended Data Table 3. Light-blue and green isolines show different combinations of the two metrics that lead to the median capacity in 1.5°C- and 2°C-compatible pathways regardless of feasibility considerations, whereas dark-blue isoline illustrates the 2040 CCS capacity indicated in the EU Industrial Carbon Management Strategy^98^. To analyse the feasible range of CCS deployment in the EU by 2040 provided that the EU Net-Zero Industry Act (NZIA) target is met by 2030 (blue line), we compare it to the feasibility frontiers shown on the figure (Supplementary Note 7). Nuclear power acceleration rate was calculated from a sample of countries that includes the former Yugoslavia. The x-axis of this figure is cut at 162 Mt/yr (10% of market potential) and 60% CAGR, thus excluding 56 1.5°C (33%) and 57 2°C pathways (16%). Density plots are constructed from the entire sample of pathways. Source data

More broadly, our research contributes to the analysis of the growth of policy-driven emerging technologies. We show how appropriate growth metrics and reference cases make it possible to develop empirically grounded feasibility spaces and feasibility frontiers for distinct phases of technological growth, and thus project its deployment based on realistic and transparent assumptions. This approach can be applied not only to global but also to national and regional targets (Fig. 1, Supplementary Note 7, Extended Data Figs. 6 and 7 and Extended Data Table 3), as well as to other climate and energy technologies that are currently in the early stages of development.

## Methods

### CCS projects database

To study current and historical CCS project plans and their failure rates, we build a dataset of completed, failed and currently planned commercial (at least 0.1 Mt yr^−1^ capacity) CCS projects starting from 1972—the completion year of the first integrated CCS project (Terrell natural gas processing plant). For each project, we code the capture rate, project announcement and completion year, facility status (for example, active, failed, planned), facility operation start and end years, CO_2_ storage type (for example, enhanced oil recovery or dedicated geological storage), sectoral and subsectoral application, country and region (Supplementary Tables 3 and 4). Data on completed and failed projects was collected from annual Global CCS Institute reports as well as dormant and existing databases listing planned CCS projects at different points in time^78–81^ (full list in Supplementary Table 2), whereas data on currently planned projects was gathered primarily from the recently published (March 2023) IEA Carbon Capture, Utilization and Storage Projects Database^7^. These sources were complemented with a systematic Google search, described in Supplementary Note 1.

To facilitate the future use of our database in combination with the IEA Carbon Capture, Utilization and Storage Projects Database, we define our variables in line with the IEA’s database and filtered our project entries to meet the minimum project capacity of 0.1 Mt yr^−1^. To facilitate the use of the database in combination with the IPCC AR6 Scenarios Database^32^, we align relevant variables with the AR6 scenario variables related to CCS. The resulting database is available in ref. ^43^.

### IPCC pathways used in this study

For analysing future trajectories of CCS deployment in the AR6 ensemble, we use mitigation pathways classified under IPCC AR6 scenario categories 1 (‘Below 1.5 °C with no or limited overshoot’), 2 (‘Below 1.5 °C with high overshoot’), 3 (‘Likely below 2 °C’), 4 (‘Below 2 °C’) and 5 (‘Below 2.5 °C’)^10^. We further group these categories into 1.5 °C- (categories 1 and 2), 2 °C- (categories 3 and 4) and 2.5 °C- (category 5) compatible mitigation pathways. Because our analysis compared future deployment trajectories for different temperature targets, we exclude model families that did not produce pathways for each of the three groups in the IPCC AR6 scenario ensemble, including TIAM (40 pathways), C-ROADS (5), EPPA (5) and MERGE (1).

Before proceeding with our analysis, we also checked scenario data for consistency with the actual CCS capacity in 2020. In the resulting sample of pathways, CCS deployment in 2020 rarely reflected its actual capacity (~35 MtCO_2_), with the majority of pathways reporting zero for 2020. We exclude 30 pathways where the CCS capacity in 2020 is reported to be more than 50 MtCO_2_ (some pathways reported up to 500 MtCO_2_ in 2020). Our final sample was 840 pathways—218 1.5 °C-, 423 2 °C- and 199 2.5 °C-compatible pathways.

Our analysis of CCS deployment covers a range of CCS technologies (Extended Data Fig. 1 and Supplementary Table 1), including those leading to negative emissions (BECCS and DACCS). Some inconsistencies related to these technologies were found among scenario outputs between model families. For example, some model families report DACCS deployment as a separate variable, others as a separate value that is also included in the total CCS variable. Some model families report DACCS and BECCS as negative values, others as positive. We harmonize these outputs and use the resulting sum of BECCS, DACCS and CCS capacity in fossil fuel and industry further in our analysis as total CCS capacity.

Lastly, we append each scenario data time series (2020–2100) with historical CCS capacity data from 2000–2020, thus replacing the zeros in 2020 (and further, if less than the actual 2020 capacity) with the latest value from our CCS Projects Database (35 MtCO_2_). The resulting dataset thus contains the 1.5 °C–2.5 °C pathways (840 pathways) with decadal CCS deployment values from 2000 to 2100.

### Defining the current and future CCS market potential

In this study, we define the market for CCS technologies as the annual gross global CO_2_ emissions in sectors where CCS is technologically applicable. To estimate today’s CCS market, we calculate CO_2_ emissions from the Emissions Database for Global Atmospheric Research (EDGAR) v.6 database^82^ in sectors and subsectors with recorded CCS plans (that is, 'Energy systems' and 'Industry' sector variables, excluding 'Other' emissions). The resulting sum of ‘capturable’ CO_2_ emissions, which we defined as *M*_2022_, results in 21 GtCO_2_.

To estimate future changes in the CCS market based on scenario data, we first calculate the sum of the CO_2_ emissions in these CCS-applicable sectors for each decade up to 2100 as the sum of the following scenario variables: Emissions∣CO_2_∣Energy∣Supply, Emissions∣CO_2_∣Energy∣Demand∣Industry, Emissions∣CO_2_∣Industrial Processes, Emissions∣CO_2_∣Waste and Emissions∣CO_2_∣Other.

These values are reported as final or net (that is, after carbon sequestration and removal). To calculate the size of the market (equation (1)), we convert the net CO_2_ emissions to gross as the sum of the former with the overall CCS capacity in the fossil-fuel-based and industrial sectors (carbon sequestration), emissions reductions achieved through non-CCS negative emissions technologies and negative emissions CCS technologies (carbon removal). Thus, the first part of equation (1) corresponds to the amount of CO_2_ entering the atmosphere in year *t* (in sectors with capturable emissions) before being captured or offset. To that, we add the BECCS and DACCS capacity as negative emissions delivered by CCS technologies in year *t* (in mitigation pathways). For an illustration of the approach, see Supplementary Fig. 4.

1 $$M_{t} = \overbrace{E_{t} + {\rm{CCS}}_{n,t} + {\rm{NET}}_{t} + {\rm{CCS}}_{ind,t}}^{{{\rm{gross}}}\,{{\rm{CO}}}_{2}\,{{\rm{emissions}}}\,{{\rm{in}}}\,{{\rm{year}}}\,{{\rm{t}}}} + \underbrace{{\rm{CCS}}_{n,t}}_{{{\rm{BECCS}}}\,{\rm{and}}\,{{\rm{DACCS}}}}$$

where:

- *M*_*t*_ is the CCS market size in year *t*;
- *E*_*t*_ is the net CO_2_ emissions in sectors where CCS technologies can be applied in year *t*;
- CCS_n,*t*_ is the sum of the biogenic and atmospheric (that is, negative) CO_2_ emissions captured and stored in year *t*;
- NET_*t*_ is the sum of the other negative CO_2_ emissions in year *t*; and
- CCS_ind,*t*_ is the sum of the fossil-based and industrial CO_2_ emissions captured and stored in year *t*.

The dataset with these variables calculated for each pathway is available in ref. ^43^. Due to missing variables, 68 pathways (~10% of the 1.5 °C- and 2 °C-compatible pathways) failed to produce this metric.

### Reference case selection

Selecting reference cases is central to the feasibility space method^27^. In this study, we use three main sets of reference cases (Table 1), one for each of the first three phases of the technology life cycle (Fig. 1). Given the centrality of the policy support shaping the expansion of CCS^42^, we use reference cases of technologies whose deployment was strongly shaped by policy support—nuclear power^50,59^, as well as solar and wind power^83–85^. These are capital-intensive technologies that have also all faced public opposition^86^ similar to the public opposition that has already started plaguing CCS projects^87^ (Supplementary Note 2). These reference technologies provided evidence of feasible CCS growth in each phase.

Given the market immaturity, capital intensity and lumpiness^9,52,53^ of CCS, we identify the project failure rates of large-scale policy-driven technologies at relatively low market penetration levels as reference cases for the failure rate of CCS projects in the formative phase. For the two less optimistic failure rates, we use both the historical failure rate of all CCS projects (88%) and a CCS subsector-adjusted rate based on current project plans (76%, see below). These failure rates are comparable to the recent failure of other emerging large-scale technologies, such as floating offshore wind power (>90%) and new applications of solar (mainly concentrated solar power, 66%)^88,89^ (Supplementary Table 5). For the optimistic case, we use the historical failure rate of nuclear power in the United States (45%) in 1972–1982^48^, when it accounted for 3% of the national electricity supply. We consider this as an optimistic benchmark for CCS projects, given that nuclear power was more commercially mature at that point.

In the acceleration and stable growth phases, we use the reference cases of nuclear, solar and wind power. In addition to being policy-driven and capital-intensive, the historical development of these technologies spans a wide range of socio-political contexts that can constrain or facilitate future CCS growth. This set of technologies varies widely in terms of modularity, complexity and the degree of customization required^52–54^, all of which might be relevant for CCS, given the variety of potential applications (for example, from the most complex BECCS to the more modular DACCS) and infrastructure needs. We validated the resulting feasibility spaces in the acceleration and stable growth phases with studies using the same^19,55^ or similar^70^ growth metrics in a wider set of technologies or contexts.

In addition, we use the historical growth of FGD as a reference case for the CO_2_ capture component of CCS^18,90^, FGD being an end-of-pipe technology without the transportation, storage, public opposition, high project costs or competitiveness challenges that CCS faces (Supplementary Note 2 and Supplementary Table 9). However, given the technological similarity to gas capture, it could be used to verify whether the capture component of CCS imposes additional constraints on the feasible speed of CCS deployment. In addition, we use evidence from FGD acceleration and growth in the sensitivity analysis on the number of IPCC pathways that clear all the feasibility constraints and the amount of CO_2_ they envision capturing (Fig. 4, Extended Data Fig. 4 and Supplementary Tables 7 and 8).

### Feasibility space for the formative phase

The formative phase is shaped by the current state of the technology and how volatile its growth is. We thus construct a feasibility space for the operational CCS capacity in 2030 (Fig. 2) using today’s planned capacity as a starting point. In projecting the operational capacity in 2030, we use two variables that shape growth volatility for emerging policy-driven technologies in the formative phase—planned capacity and its failure rate.

For the first variable, we assume the CCS plans to no more than double (to 600 Mt yr^−1^) from 2023 to 2025 (annual growth ~42%). This is in line with the annual growth rates in 2021 and 2022, which have been steadily declining since the start of the second wave, from 108% in 2019 to 43% in 2022. Due to the small number of projects planned to start operations by 2025^5^, we assume possible early project success to be insufficient to ignite industry interest beyond this ceiling by 2025. We also assume 5 years for project implementation, which is the average project delivery time of active and completed CCS projects. This is in line with recent estimates of 4 to 7 years^91^. Nevertheless, we account for a potentially longer project delivery time, which is characteristic of large-scale projects^51,92^, by using the currently planned capacity as the feasible minimum.

For the second variable, we calculate the share of planned capacity that fails to become operational by 2030 using three reference cases (Table 1). For the first failure reference case, we estimate the historical failure rate of all CCS projects (88%, equation (2)).

2 $${F}_{{\mathrm{hist}}}=\sum \frac{{\mathrm{CC{S}}}_{{\mathrm{f}},1972-2017}}{{\mathrm{CC{S}}}_{{\mathrm{p}},1972-2017}}\times 100 \%$$

where:

- *F*_hist_ is the historical CCS failure rate;
- *CCS*_f,1972−2017_ is the capacity of failed projects between 1972 and 2017; and
- CCS_p,1972−2017_ is the capacity of planned projects between 1972 and 2017.

For our second failure reference case, we calculate a subsector-adjusted failure rate (76%), which is an average of the historical subsectoral failure rates (Supplementary Table 1) weighted by their share in 2022 project plans. First, we calculate a failure rate for each subsector (equation (3)). Then, we calculate the average failure rate weighted by the share of each subsector in current project plans (equation (4)). If the application of CCS is new (for example, BECCS electricity), with only a few projects planned historically (*n* < 5), we use the historical failure rate of all CCS projects (88%).

3 $${F}_{s}=\sum \frac{{\mathrm{CC{S}}}_{{\mathrm{f}},s,1972-2017}}{{\mathrm{CC{S}}}_{{\mathrm{p}},s,1972-2017}}\times 100 \%$$

4 $${F}_{{\mathrm{adj}}}=\sum {F}_{s}\times \frac{{\mathrm{CC{S}}}_{{\mathrm{p}},s,2022}}{{\mathrm{CC{S}}}_{{\mathrm{p}},2022}}\times 100 \%$$

where:

- *F*_*s*_ is the historical failure rate in subsector *s*;
- CCS_f_ is the capacity of failed projects between 1972 and 2017 in subsector *s*;
- CCS_p_ is the capacity of planned projects between 1972 and 2017 in subsector *s*;
- *F*_adj_ is the subesector-adjusted failure rate of current CCS plans;
- CCS_p,s,2022_ is the planned capacity of projects in subsector *s* in 2022; and
- CCS_p,2022_ is the total capacity of planned projects in 2022.

Finally, for the third CCS failure rate reference case, we use a decadal failure episode of nuclear power deployment in the United States (1972–1982), as documented in ref. ^48^. The year when the first failures occurred was 1972, so some projects that failed from 1972 to 1982 had been planned before 1972. During this time period, we calculate a failure rate of 45% for all planned nuclear power plant projects. We find this episode to be a fitting reference case for the current plans for CCS globally for several reasons. First, in 1972, nuclear power production was still concentrated in a few pioneering countries led by the United States, and the global share of nuclear power in electricity production was less then 2.5%, which means it was close to the formative phase. Second, CCS and nuclear power share several similarities in terms of technological characteristics, such as lumpiness^52,53^, the need for customization^53,54^ and capital intensity^9^.

### Feasibility space in the acceleration phase

The level of technological development during the acceleration phase depends on the rate of growth and the initial level from which growth starts. We construct a feasibility space for the acceleration phase, with the potential market penetration in 2030 for the latter and the CAGR between 2030 and 2040 for the former. We measure the 10-year CAGR for three reference cases of policy-driven emerging technologies—wind, solar and nuclear power—from the time they reached similar levels of market penetration compared to what can realistically be achieved by CCS in 2030 (0.3–1.8%). For nuclear power, this period corresponds to 1961–1968, when nuclear power production had grown from 0.15% (current market share of CCS) to 1.8% (maximum achievable market share of CCS by 2030) of its maximum production in 2006. For comparison, in 1961, there were 15 operational nuclear reactors worldwide^75^. For wind power, which has not yet reached its maximum capacity, we took the period between 1995 and 2007, when wind power production grew from 0.06% to 0.7% of global electricity production, which roughly corresponds to 0.15% and 1.8%, respectively, assuming the market potential of wind power is about 40% of the electricity supply^19^. For market penetration rates from 0.3–1.8% in these two reference cases, achieved every year in selected periods (above), we estimated the acceleration rate for the following 10 years (equation (5)). For solar power, it has been less than 10 years since it gained 1.8% of its global market potential, therefore it forms a narrow segment of the feasibility frontier (from 2008, when it gained 0.15%, up to 2012, when it gained 1.1% (ref. ^77^), assuming the market potential of solar power is about 40% of the electricity supply^19^). Taken together, three time series form three *y*-axis feasibility frontiers of CCS acceleration in Fig. 3. From different combinations of *x*- and *y*-axis metrics, we estimate the feasible range of CCS deployment by 2040. In addition, we measure the acceleration of FGD normalized to the total coal power capacity, as a reference case for deployment of the CO_2_ capture component of CCS.

To map the IPCC climate pathways onto the feasibility space for acceleration, we calculate the market penetration level of CCS in 2030 by dividing the CCS capacity in 2030 in each pathway (CCS_2030_) by the current amount of capturable emissions in sectors where CCS projects are currently planned (*M*_2022_). Then, we calculate the CCS growth rates in the acceleration phase in 2030–2040 using equation (5). Taken together, these two variables formed the 2D density plot (R package ‘ggdensity’^93^) for CCS acceleration in mitigation pathways (Fig. 3).

5 $${A}_{{\mathrm{CCS}}}=\left({\left(\frac{{\mathrm{CC{S}}}_{t+10}}{{\mathrm{CC{S}}}_{t}}\right)}^{\frac{1}{10}}-1\right)\times 100 \%$$

where:

- *A*_CCS_ is the growth rate in the acceleration phase; and
- CCS_*t*_ is the CCS capacity (including BECCS and DACCS) in year *t*.

### Feasibility space in the stable growth phase

In the stable growth phase, the mechanisms supporting growth are balanced out by those slowing it down, which can be measured with the metric *G*, introduced in ref. ^19^. We construct a feasibility space for the stable growth phase based on the maximum growth rate and when this growth rate was achieved. To measure the *G* implied in the IPCC pathways, we fit the Gompertz (equation (6)) and logistic growth (equation (7)) models to a CCS deployment time series in 2030–2100 combined with the historical data on operational CCS capacity in 2000–2020. Because we are only interested in the stable growth rate, we truncate the resulting time series at the maximum annual CCS capacity to increase the goodness of fit in pathways where CCS deployment starts to decrease after stable growth and saturation. In such pathways, CCS is never phased out by the end of the century.

6 $${f}_{{\mathrm{gmp}}}(t)=L{e}^{-{e}^{-k(t-{t}_{0})}}$$

7 $${f}_{{\mathrm{log}}}(t)=\frac{L}{1+{e}^{-k(t-{t}_{0})}}$$

where:

- *e* is a constant approximately equal to 2.718;
- *k* is the growth constant; and
- *t*_0_ is the inflection point.

For the logistic curve, the inflection point is located at 50% of the asymptote, *L*. For the Gompertz curve, the inflection point is located at 37% of the asymptote, *L*.

The *G* for the maximum annual addition of CCS capacity (GtCO_2_ yr^−2^) in each pathway is calculated as follows:

8 $${G}_{{\mathrm{gmp}}}=\frac{Lk}{e}$$

9 $${G}_{{\mathrm{log}}}=\frac{Lk}{4}$$

To account for the uncertainties concerning the size and dynamics of the potential CCS market, which can either expand due to the increased use of fossil fuels or adoption of negative emissions technologies, or shrink due to climate change mitigation action, we normalize *G* to the market size. We use two normalization options—today’s market size (*M*_2022_^82^) and the market size at the inflection point (*M*_TMax_). For the latter, scenario outputs only contain decadal data, which does not allow precise measurement of the market size in the year of maximum growth. To obtain approximate estimates of this value, we assume the linear development of emissions trajectories within each decade (equation (10)).

10 $${M}_{{\mathrm{TMax}}}={M}_{t}+\frac{{M}_{t+10}-{M}_{t}}{10}\times ({\mathrm{TMax}}-t)$$

where:

- TMax is the year of the inflection point (and maximum growth);
- *t* and *t* + 10 are the years between which TMax is located (for example, 2030 and 2040); and
- *M*_*t*_ is the market size at year *t*.

These two normalization options result in two maximum growth rate variables—*G*_2022_ and *G*_TMax_. Normalization to market size enables us to compare these growth rates with global, regional and national reference cases for nuclear, solar and wind power, where the maximum growth was normalized to the size of the market. In the cases where solar and wind power are still accelerating, *G*_TMax_ could not be reliably estimated, and therefore we instead use a similar metric of maximum growth, *R*_3_—the average added production over the last three years normalized to the average market size over those years (equation (11),^19,55^). These instances are reported in Extended Data Table 1, where TMax is not reported. In addition, we measure the *R*_3_ of FGD (due to data availability^18^), normalized to the total coal power capacity as a reference case for the maximum growth of the CO_2_ capture component of CCS. Extended Data Fig. 3 displays the IQR ranges for the *G*_TMax_ estimated from cases where the stable growth phase had already been reached^19,55^.

11 $$R_3=\frac{\left(\frac{{P}_{t}-{P}_{t-3}}{3}\right)}{\left(\frac{{M}_{t}+{M}_{t-3}}{2}\right)}\times 100 \%$$

where:

- *R*_3_ is a metric used instead of *G*_TMax_ for reference cases where the maximum growth has not yet been achieved;
- *P*_*t*_ is the production in year *t*; and
- *M*_*t*_ is the market size in year *t*.

For regional reference cases, we report the maximum growth rate of nuclear power deployment in the Organization for Economic Cooperation and Development region (with membership as of 1990). For solar and wind power deployment, we estimate the maximum growth for the Organization for Economic Cooperation and Development, the 27 countries of the European Union and Asian regions, with the latter two reported in Extended Data Table 1 as the fastest for wind and solar, respectively. For national reference cases in this table, we estimate the maximum growth rate in countries with electricity markets of around 100 TWh (or more), which we consider to be the most relevant for climate change mitigation and this analysis. In Extended Data Table 1, we report regional and five national reference cases with the fastest maximum growth rate together with global values. In Extended Data Fig. 3, the IQRs of the maximum growth rates in the national reference cases are estimated based on all cases, regardless of the size of the electricity market.

We also calculate the duration of CCS diffusion from 10% to 90% of its maximum capacity, envisaged in mitigation pathways, or Δ*t* (equations (12) and (13)).

12 $$\Delta {t}_{{\mathrm{gmp}}}=\frac{\ln \left(\frac{\ln (0.1)}{\ln (0.9)}\right)}{k}$$

13 $$\Delta {t}_{{\mathrm{log}}}=\frac{\ln (81)}{k}$$

### Vetting of mitigation pathways using feasibility constraints

In vetting the IPCC AR6 climate mitigation pathways with the proposed metrics, we combine the three feasibility spaces for analysing the feasible range of CCS deployment in the formative, acceleration and stable growth phases. Our upper constraints for each step are:

- Formative phase: up to a 45% failure rate and a doubling of planned CCS capacity by 2025. This constraint results in the maximum feasible CCS capacity by 2030 (0.37 Gt yr^−1^).
- Acceleration phase: the acceleration rate of CCS in 2030–2040 is never faster than the highest acceleration observed for any of our reference cases in the acceleration phase (wind, solar and nuclear power).
- Stable growth phase: up to a 1.45% maximum annual growth rate. This global maximum annual growth rate was achieved by nuclear power in 1984 (average for Gompertz and logistic model fits). This constraint represents the maximum feasible CCS growth rate that can occur any time after take-off.

We measure the cumulative amount of CO_2_ captured and stored by 2070 and 2100 in the IPCC AR6 climate mitigation scenarios by summing the linearly extrapolated capacity of CCS technologies between each decade, starting from 2030.

### Reporting summary

Further information on research design is available in the Nature Portfolio Reporting Summary linked to this article.

## Online content

Any methods, additional references, Nature Portfolio reporting summaries, source data, extended data, supplementary information, acknowledgements, peer review information; details of author contributions and competing interests; and statements of data and code availability are available at 10.1038/s41558-024-02104-0.

## Supplementary information

Supplementary Information Supplementary Figs. 1–11, Tables 1–11 and Notes 1–7.

Reporting Summary

## Source data

Source Data Fig. 2 Statistical Source Data

Source Data Fig. 3 Statistical Source Data

Source Data Fig. 4 Statistical Source Data

Source Data Extended Data Fig. 2 Statistical Source Data

Source Data Extended Data Fig. 3 Statistical Source Data

Source Data Extended Data Fig. 4 Statistical Source Data

Source Data Extended Data Fig. 5 Statistical Source Data

Source Data Extended Data Fig. 6 Statistical Source Data

Source Data Extended Data Fig. 7 Statistical Source Data

## Figures and Tables

**Extended Data Table 1:**
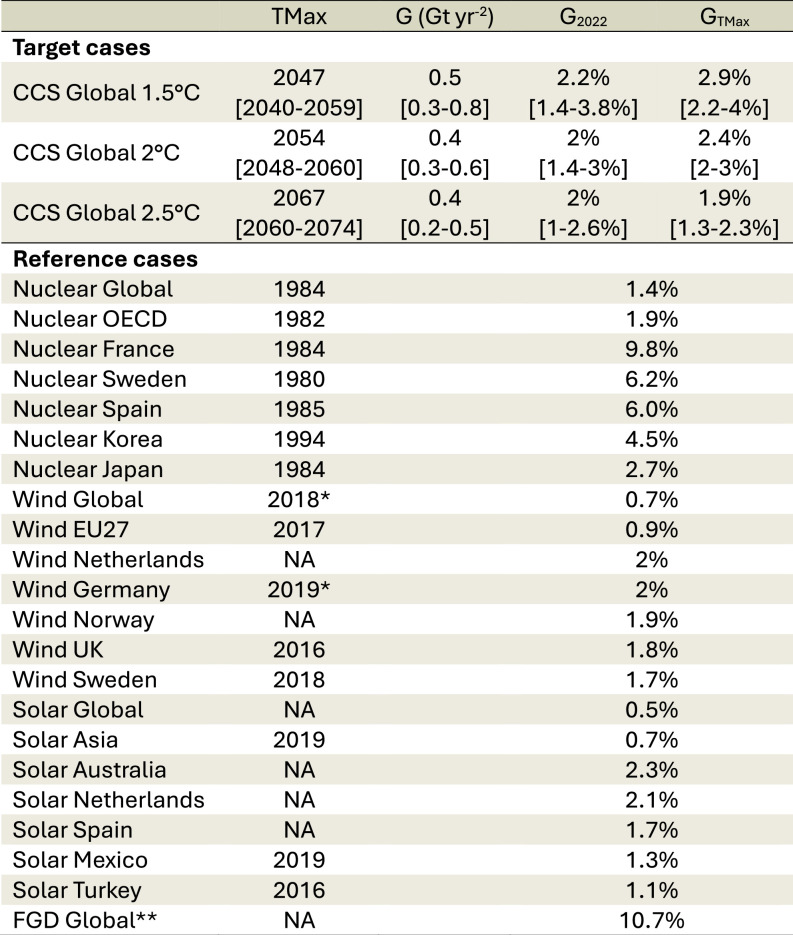
Maximum growth rate of CCS capacity in the stable growth phase in the IPCC AR6 pathways compared to observed maximum growth of reference cases

TMax - the year when maximum growth is achieved; G - maximum annual capacity additions of CCS; G_2_022 - maximum growth rate of CCS normalised to the maximum market size in 2022; G_*TMax*_ - maximum growth rate of CCS normalised to the maximum market size at TMax. For target cases^10,32^, values show the median and the IQR (in brackets) of average parameters of Gompertz and logistic fits. For reference cases (Table 1), we report the global G_*TMax*_ as well as the highest observations of G_*TMax*_ in regional and national markets (Methods) - values show average parameters of Gompertz and logistic fits unless otherwise specified. For reference cases where the maximum growth has not yet been achieved (that is TMax is not in the past) or there is insufficient data, we calculate the maximum growth rate over three years (R3): 2017-2020 for Solar Global; 2005-2008 for FGD Global; 2018-2021 for the rest (Methods). Interquartile ranges of G_*TMax*_ observations in all national markets for each reference case are shown in Extended Data Fig. 3. FGD - flue gas desulfurisation. *TMax of logistic fit^55^; **FGD is used as a reference case for the CO_2_ capture component of CCS.

**Extended Data Table 2:**
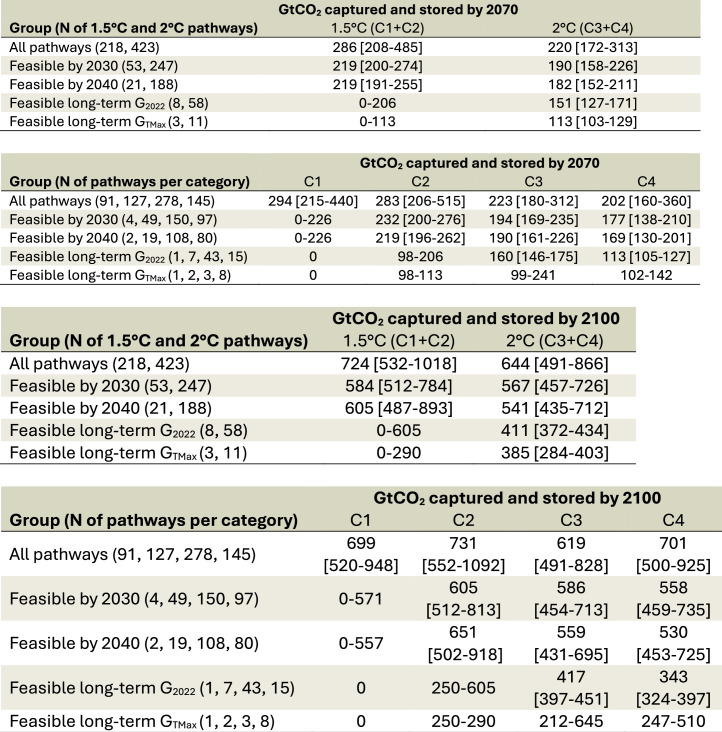
The effect of feasibility constraints at formative, acceleration, and stable growth phases used in this study on long-term cumulative capture and storage of CO_2_ in Gt by 2070 and 2100 for meeting climate targets

The table shows different groups of pathways before (’all pathways’) and after imposing feasibility constraints (that is by 2030, 2040, and over the long-term) for 1.5°C- and 2°C-compatible IPCC AR6 pathways (top) as well as for IPCC Scenario Categories (bottom of each panel). Categories vary by the global mean surface air temperature change by 2100: C1 pathways stay below 1.5°C with no or limited overshoot with a 50% probability; C2 - below 1.5°C with high overshoot; C3 - likely below 2°C; C4 - below 2°C^10^. C1 and C2 are thus grouped as 1.5°C-compatible; C3 and C4 as 2°C-compatible. Numbers in brackets in the first column show the number of pathways in each group. Numbers in the following columns show median values of CO_2_ captured and stored by 2070 (upper panel) and 2100 (lower panel); numbers in square brackets show the interquartile range of these values. Full ranges are reported for pathway groups with N < 10

**Extended Data Table 3:**
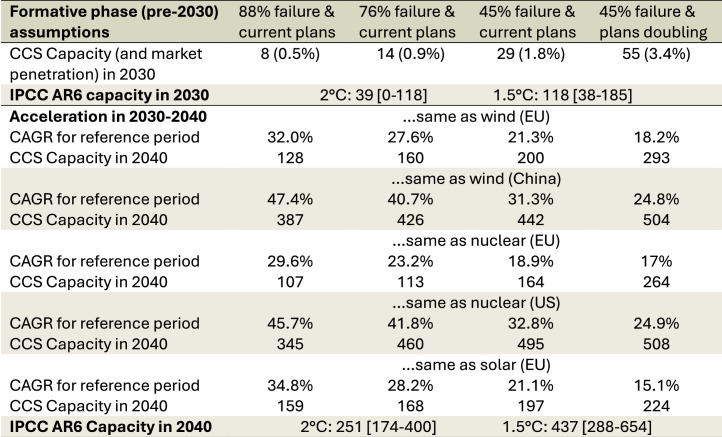
Feasible upper bounds of CCS capacity in the EU in 2030 and 2040 estimated from reference cases and compared to the IPCC AR6 mitigation pathways

Columns illustrate different outcomes at the formative phase (that is CCS deployment by 2030), whereas rows illustrate the compound annual growth rate (CAGR) for each 10-year reference period and the resulting CCS capacity by 2040. Values are reported in Mt/yr unless otherwise specified. For IPCC pathways, numbers show median values of CO_2_ captured and stored by 2040, and numbers in square brackets show the interquartile range of these values

## Data Availability

The data for this analysis, including the dataset of historical and planned CCS projects, are available via Zenodo at 10.5281/zenodo.12706872 (ref. ^43^) and GitHub at https://github.com/poletresearch/CCS_article. For our analysis of CCS deployment in climate-constrained scenarios, we used the three most recent IPCC scenario ensembles—AR5 (ref. ^33^), SR1.5 (refs. ^34,94^) and AR6 (refs. ^10,32^). For the historical acceleration of wind and the stable growth rates of wind and solar electricity production, we used IEA World Energy Balances^76^. For the historical acceleration of solar, we used EMBER yearly electricity data^77^. For the historical growth of nuclear, we used the United Nations Statistics Division Energy Statistics Database^75^. Source data are provided with this paper.

## Code availability

The code for this analysis is available via Zenodo at 10.5281/zenodo.12706872 (ref. ^43^) and GitHub at https://github.com/poletresearch/CCS_article. The code for curve fitting used in this article was originally developed by Cherp et al.^19^ and is available at the GitHub repository of the article https://github.com/poletresearch/RES_article.
