# Supplementary material for: Feasible deployment of carbon capture and storage and the requirements of climate targets
Source: Nat Clim Chang. 2024 Sep 25;14(10):1047–55. doi: 10.1038/s41558-024-02104-0 (PMC11458486; doi:10.1038/s41558-024-02104-0)
Supplement: Supplementary file 1 — Supplementary Figs. 1–11, Tables 1–11 and Notes 1–7. [file 41558_2024_2104_MOESM1_ESM.pdf]

nature climate change

Supplementary information https://doi.org/10.1038/s41558-024-02104-0

Feasible deployment of carbon capture and storage and the requirements of climate targets

In the format provided by the authors and unedited

Table of Contents

1 Supplementary Figures **2**

2 Supplementary Tables **13**

3 Supplementary Notes **22**

1 Supplementary Figures

Fig. S1 | Feasibility space of CCS deployment for the acceleration phase in 2030-2040 compared to IPCC AR6 pathways. CCS capacity and market penetration achieved by 2030 (x-axis) versus a 10-year moving compound annual growth rate (CAGR) in 2030-2040 (y-axis). 1.5°C- and 2°C-compatible pathways [1, 2] shown as dots and their distribution forms the 2D-density plot (from purple to yellow). Black lines show historical acceleration rates of nuclear (1961-1978), wind (1995-2017), and solar power (2008-2022) as reference cases for CCS (Table 1). Dark red line shows the historical acceleration of FGD (1972-1986) as a reference case for the capture component of CO_2. Reference cases are shown in the context of ref. [3] who calculate a similar metric for a broader range of technologies (Methods). The maximum feasible capacity in 2030 makes up the feasibility frontier along the x-axis (0.37 Gt/yr or 1.8% of the market, Fig. 2). Acceleration for reference cases make up the three feasibility frontiers (black lines) for CCS acceleration in 2030-2040. Dashed lines illustrate the continuation of these reference cases under higher than realistic CCS capacity by 2030. Blue and green isolines show different combinations of the two metrics that lead to the median CCS capacity in 1.5°C- and 2°C-compatible pathways respectively (Table 2) regardless of feasibility considerations. The x-axis of this figure is cut at 10% capacity (ca. 2.1 Gt/yr), thus excluding 47 1.5°C pathways (20%) and 33 2°C pathways (8%) with CCS capacity up to 21 Gt/yr by 2030. Density plots are constructed from the entire sample of pathways (n=218 for 1.5°C, n=423 for 2°C).

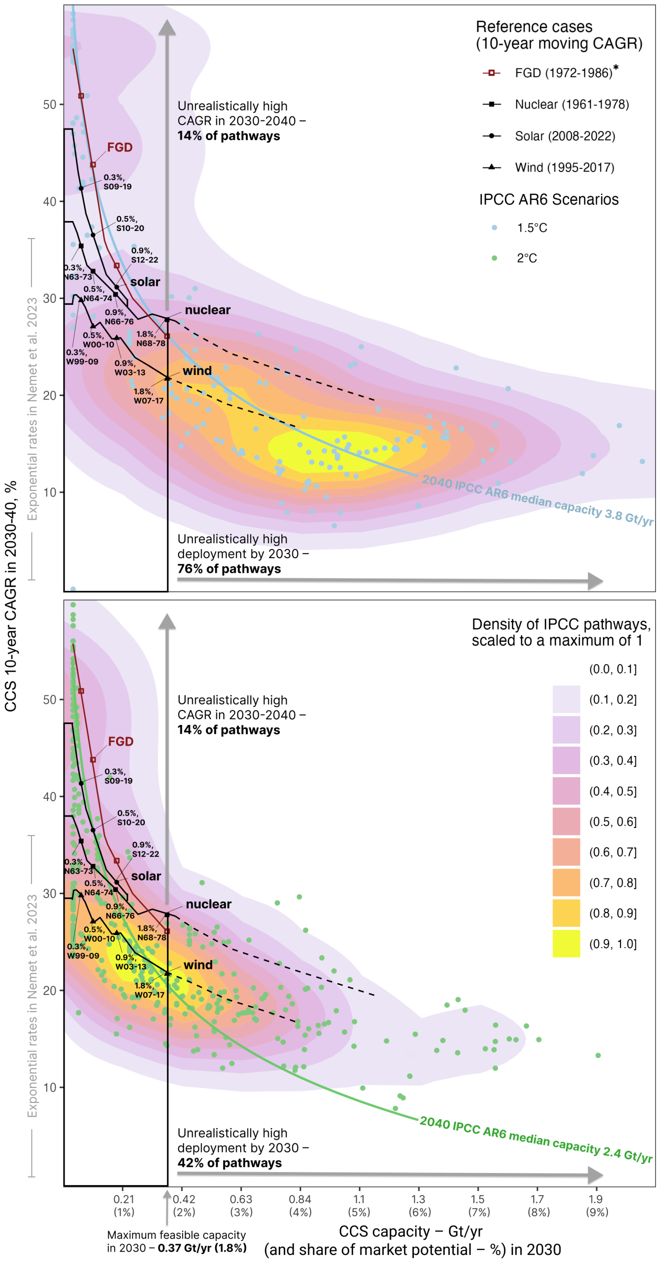

Fig. S2 | CCS capacity (incl. BECCS and DACCS) for reaching different temperature targets in the IPCC AR6 scenario ensemble [2]. This figure illustrates CCS capacity (Gt/yr) among C1-C6 IPCC Scenario Categories in 2030-2100. Categories vary by the global mean surface air temperature change by 2100: C1 pathways stay below 1.5°C with no or limited overshoot with a 50% probability (n=91); C2 - below 1.5°C with high overshoot (n=127); C3 - likely below 2°C (n=278); C4 - below 2°C (n=145); C5 - below 2.5°C (n=199); C6 - below 3.0°C (n=88) [1]. C1 and C2 are thus grouped as 1.5°C-compatible; C3 and C4 as 2°C-compatible; C5 as 2.5°C- and C6 as 3°C-compatible. Numbers in brackets show the number of pathways in each category (Methods). The box-plots show the interquartile range (IQR) with the median marked with a black line and whiskers extending from the IQR range to nonoutlier minimum and maximum. Violin plots show the distribution of these values within each pathway group.

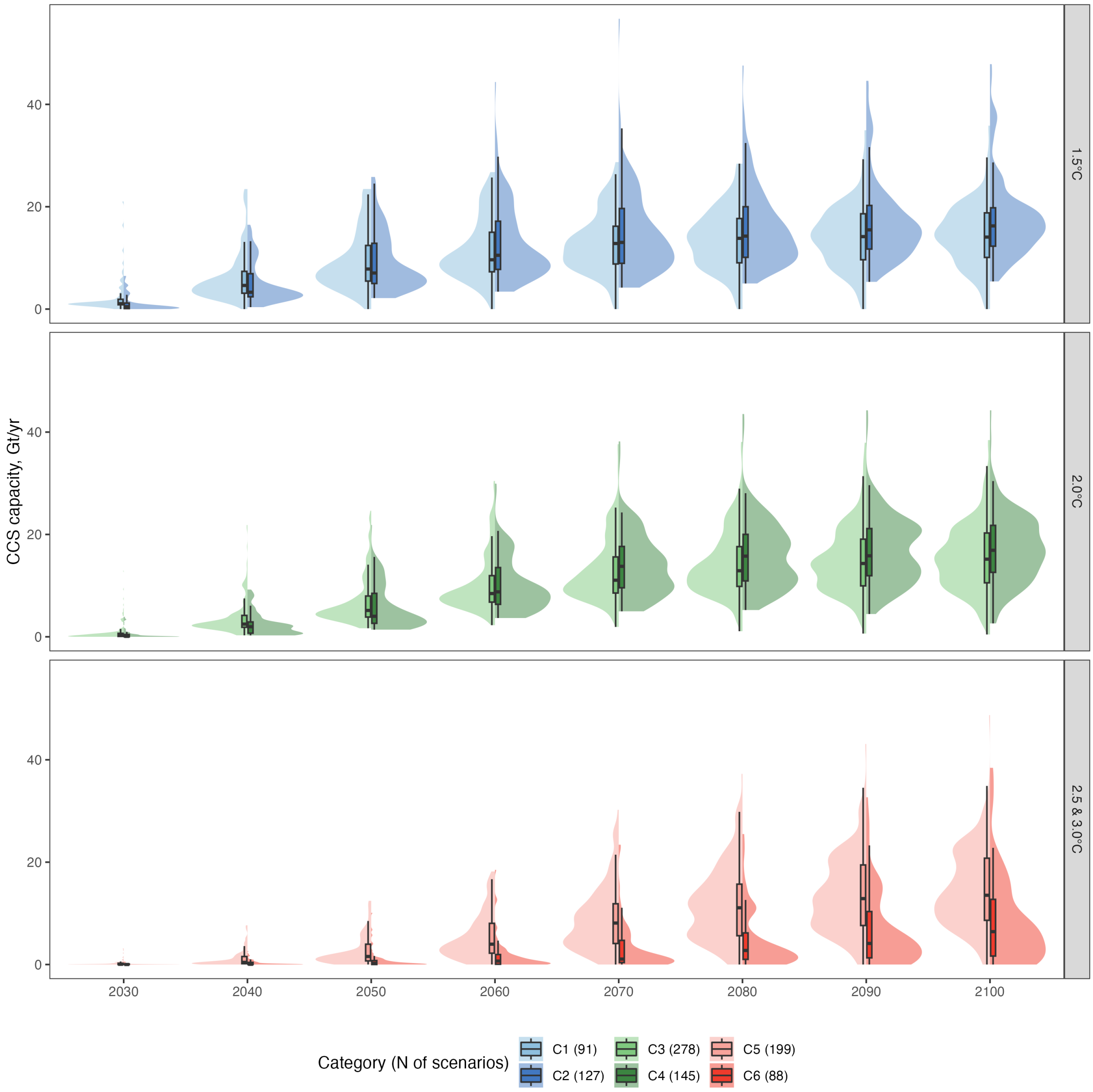

Fig. S3 | Average growth model parameters of CCS deployment in the IPCC AR6 scenario ensemble, grouped by temperature outcome. **a**, maximum annual growth rates (G, Gt/yr), the year of maximum growth ($TMax$), and maximum capacity (saturation level, in Gt/yr) in 1.5°C-, 2°C-, and 2.5°C-compatible pathways. **b**, interquartile ranges (IQR) of G by temperature group (n=218 for 1.5°C, n=418 for 2°C, n=199 for 2.5°C). **c**, IQR of the year of maximum growth ($TMax$) by temperature group (same samples as in panel B). The box-plots show the interquartile range (IQR) with the median marked with a black line and whiskers extending from the IQR range to nonoutlier minimum and maximum. Each parameter is calculated from fitting Gompertz and logistic growth function (Methods). Average values of the two models are used. Panel A of this figure does not display 18 outliers (<2% of pathway sample) where G>2 Gt/yr or where $TMax$=NA due to no deployment of CCS – these observations are still included in the calculation of interquartile ranges in panels B and C. Detailed data on parameters for each model are available in ref. [4].

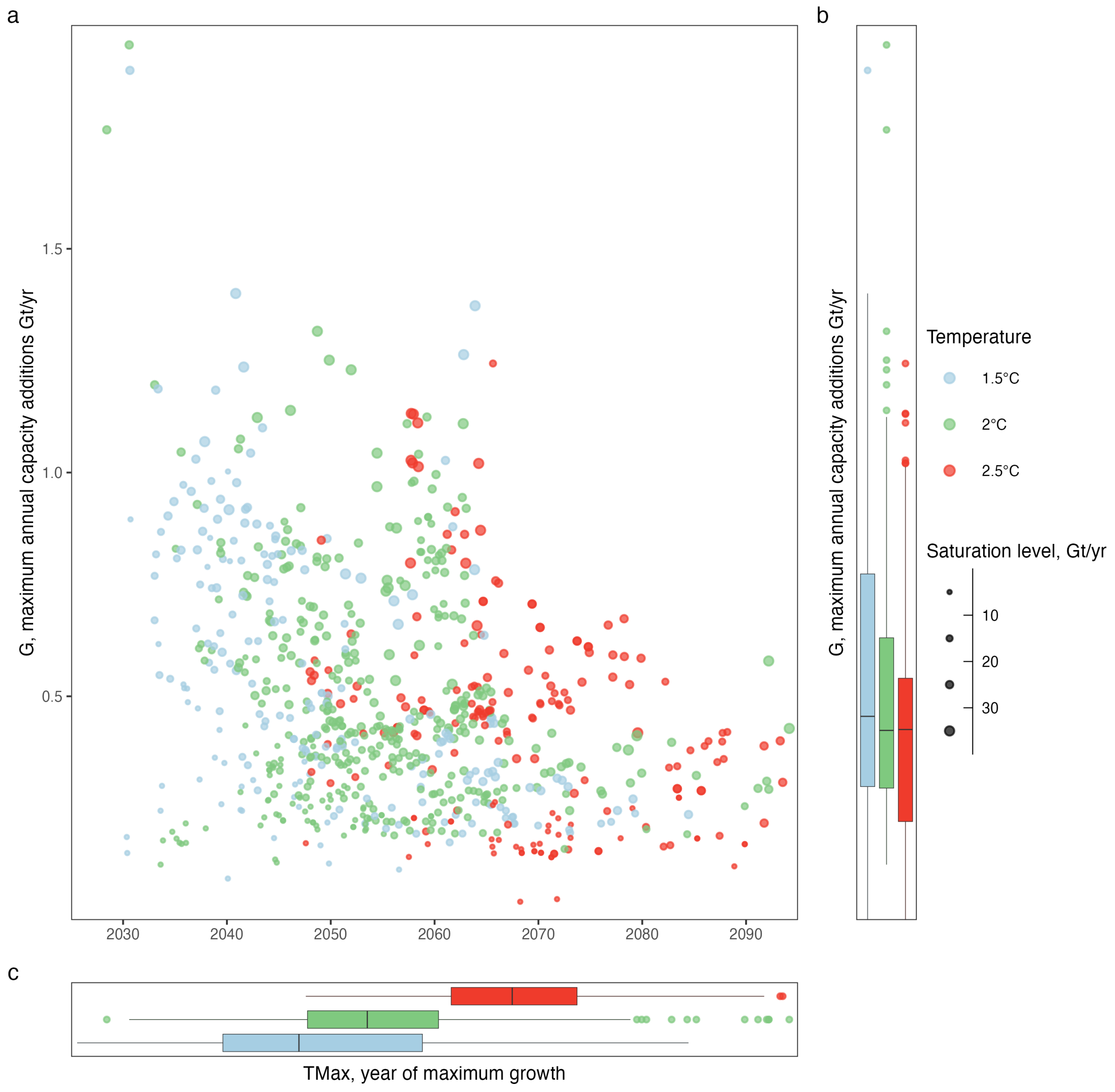

Fig. S4 | Illustration of the approach to estimate potential CCS market in the IPCC AR6 scenario ensemble (Methods). To calculate the size of the market, we convert net CO_2 emissions (black line) to gross as the sum of the former with the overall CCS capacity in fossil-fuel based and industrial sectors (blue), emission reductions achieved through non-CCS negative emission technologies (pink) and negative emission CCS technologies (green). Thus, grey and blue areas together correspond to the amount of CO_2 that enters the atmosphere in year t (in sectors with capturable emissions) before being captured or offset. To that, we add BECCS and DACCS capacity (green) as negative emissions delivered by CCS technologies in year t (in climate pathways).

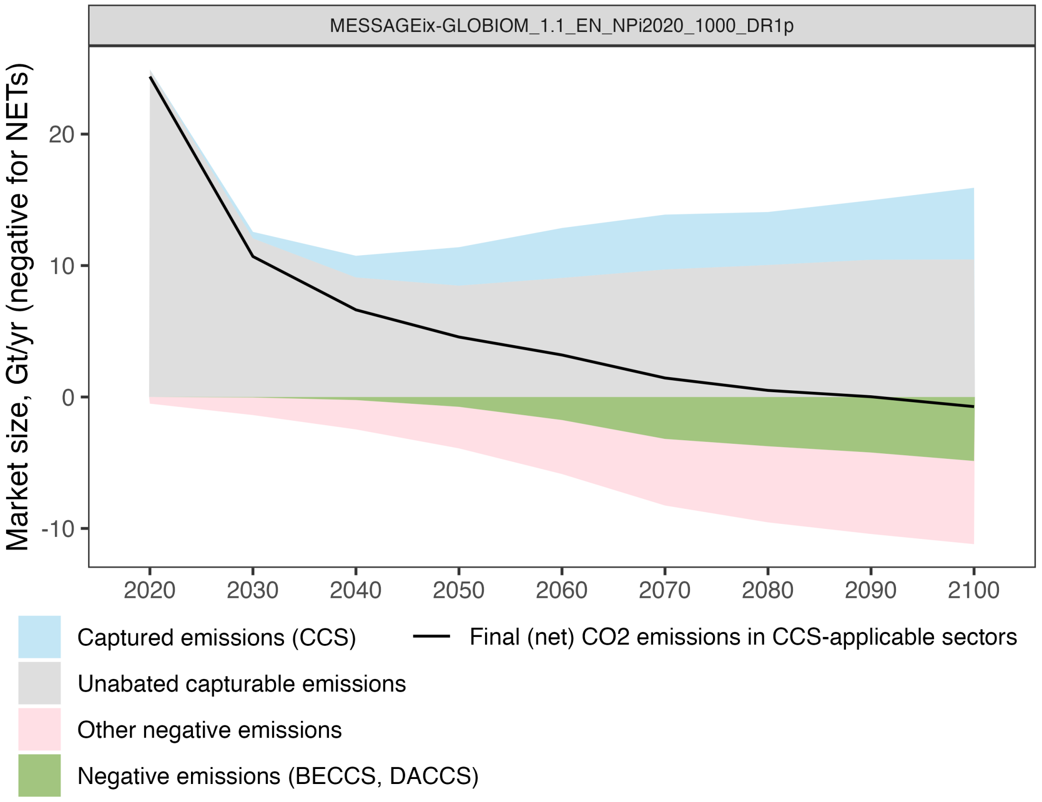

Fig. S5 | Sensitivity analysis of long-term outcomes of CCS deployment to the more optimistic and more pessimistic assumptions about the metrics of the three phases of growth in 1.5°C- and 2°C-compatible IPCC AR6 pathways. The figure illustrates how different combinations of constraints (Table 1) in the formative, acceleration (x-axis), and stable growth (columns) phases affect the cumulative CO_2 capture and storage between 2030 and 2070 (left y-axis) and the share of IPCC pathways that satisfy these assumptions (right y-axis). On the x-axis, 'All pathways' depicts all IPCC pathways [2]: n=218 for 1.5°C in top panels and n=423 for 2°C pathways in bottom panels. 'OptF' and 'PessF' depict optimistic and pessimistic constraints in the formative phase (0% failure rate and plans doubling between 2023 and 2025; 88% and current plans); 'OptA' and 'PessA' are optimistic and "pessimistic" constraints in the acceleration phase (acceleration rate of FGD and wind respectively, Fig. 3), 'CentralF' and 'CentralA' are the assumptions about the formative and acceleration phases used throughout this study (Table 1, Fig. 4, Methods). As for the stable growth phase, the right column ('G2022 w/o FGD') of the figure depicts the outcomes with the assumption of maximum growth rate used throughout this study (global nuclear power deployment), whereas the left column ('G2022 w/ FGD') depicts the outcomes with the assumption of the maximum growth rate of global FGD deployment (Extended Data Table 1). Violins and boxplots illustrate cumulative CO_2 capture and storage between 2030 and 2070 (in Gt, left y-axis): the box-plots show the interquartile range (IQR) with the median marked with a black line and whiskers extending from the IQR range to nonoutlier minimum and maximum. Grey bars illustrate the share of pathways in each group (right y-axis) relative to 'All pathways'. Black horizontal line marks cumulative capture of 200 Gt. See Supplementary Table 7 for detailed results of the sensitivity analysis.

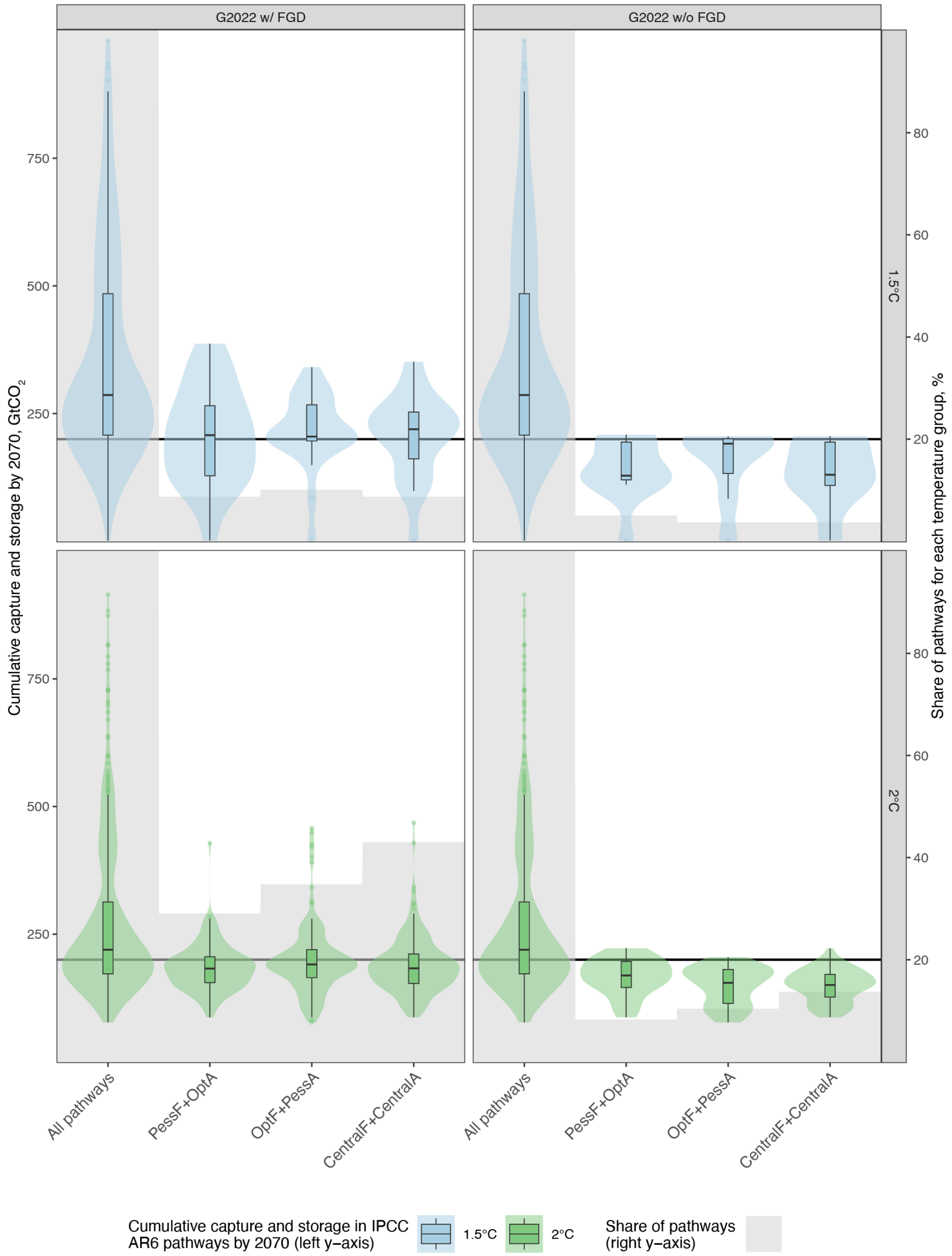

Fig. S6 | Relationship between medium-term (2040) CCS capacity (Gt/year) and long-term amount of CO_2 captured and stored (by 2070, in $GtCO_2$) in the IPCC AR6 pathways. The relationship is illustrated separately for IPCC AR6 1.5°C- (n=218) and 2°C-compatible (n=423) mitigation pathways [2]. Blue line illustrates the linear regression trend, whereas grey band illustrates a 95% confidence interval.

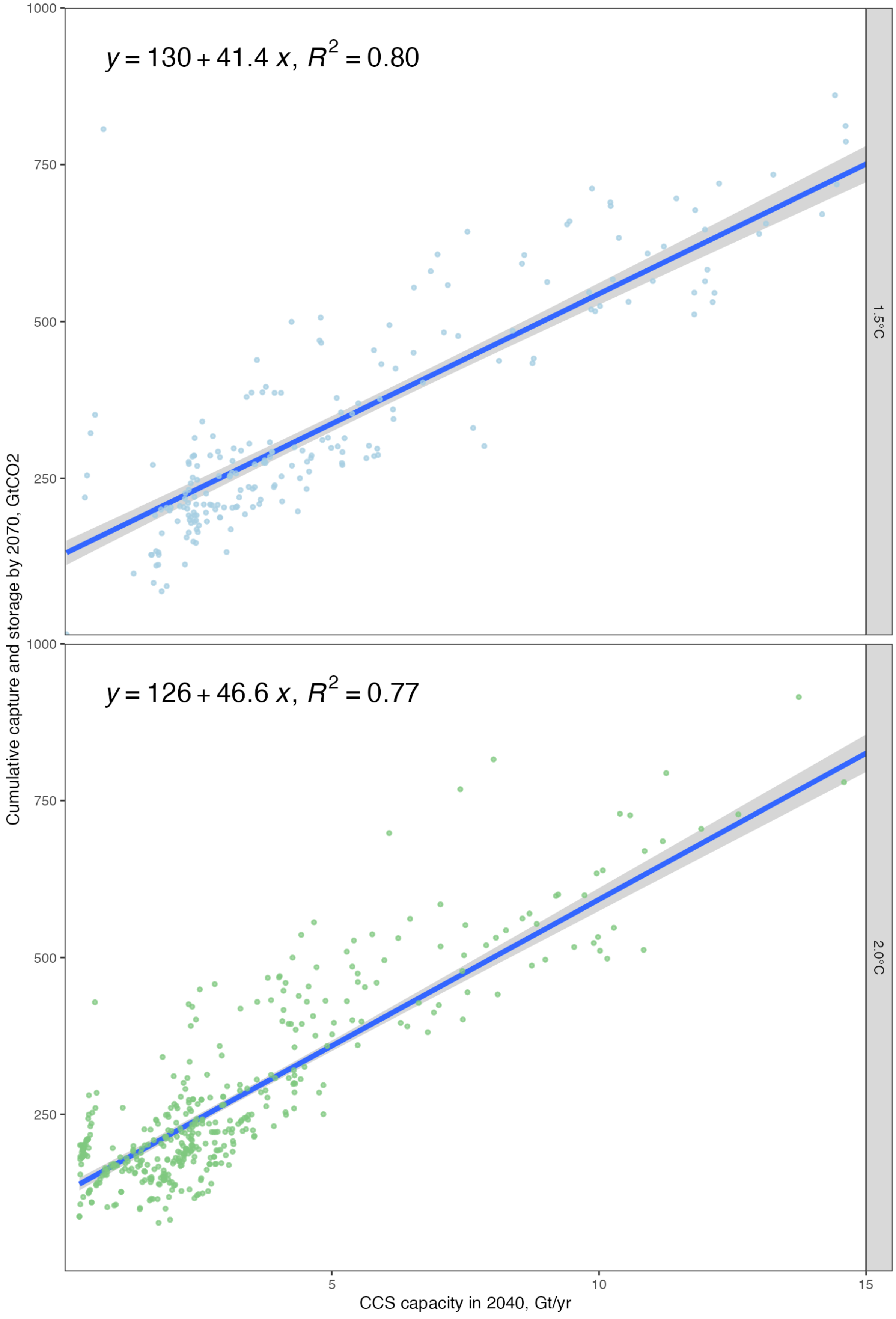

Fig. S7 | Gompertz and logistic curve fit of CCS deployment in mitigation pathways [2] consistent with our analytical approach to project feasible deployment of policy-driven technologies (i.e. "vetted", Methods). Colored lines illustrate growth curve fits – Gompertz (orange) and logistic (blue), dots illustrate inflection points of each curve. IMP-LED pathway (first row, second column) is displayed empty as it does not project any CCS capacity.

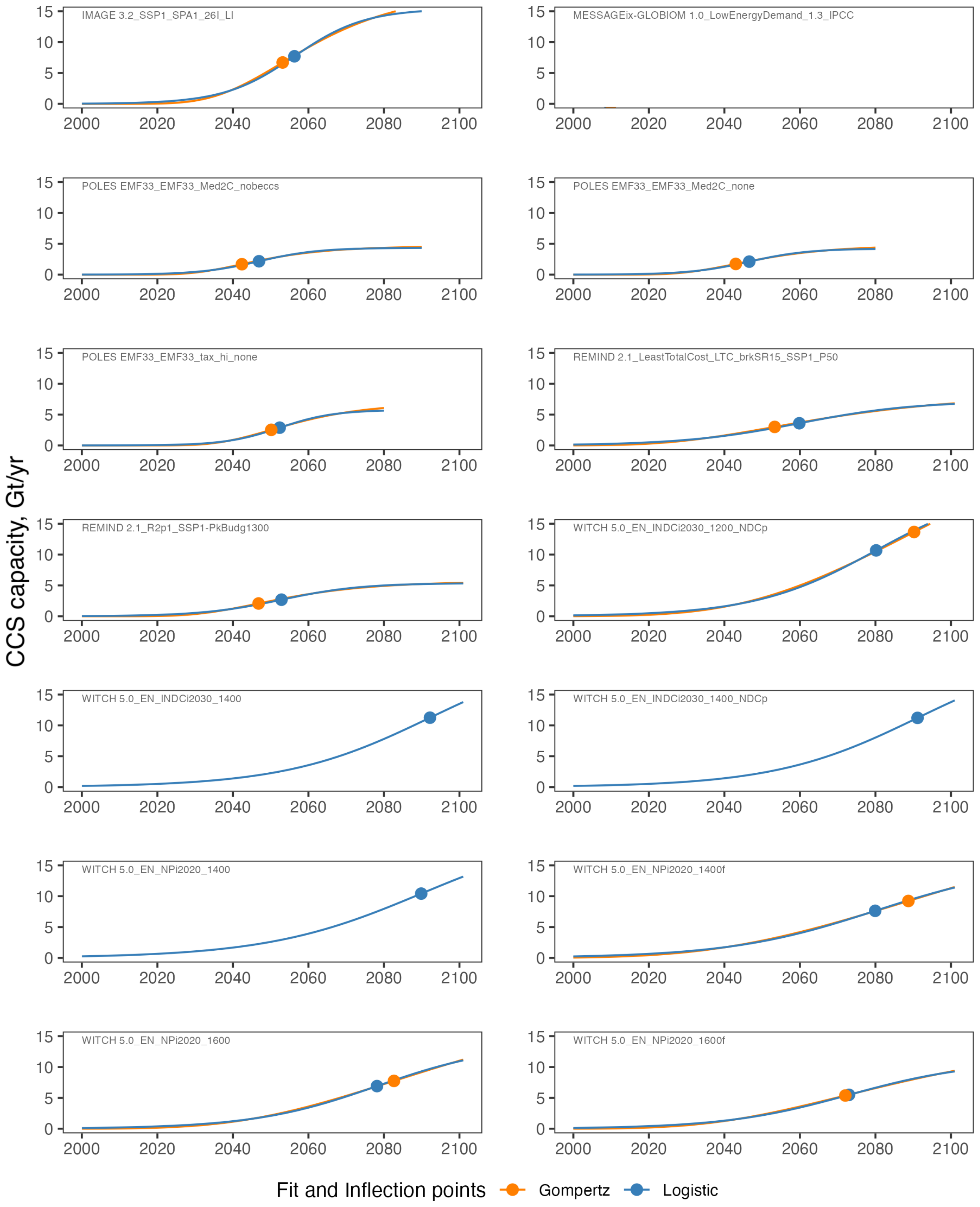

Fig. S8 | CCS market size over time in mitigation pathways [2] consistent with our analytical approach to project feasible deployment of policy-driven technologies (i.e. "vetted", Methods). See Supplementary Fig. 4 and Methods for the illustration and description of the approach to estimate potential CCS market in the IPCC AR6 scenario ensemble.

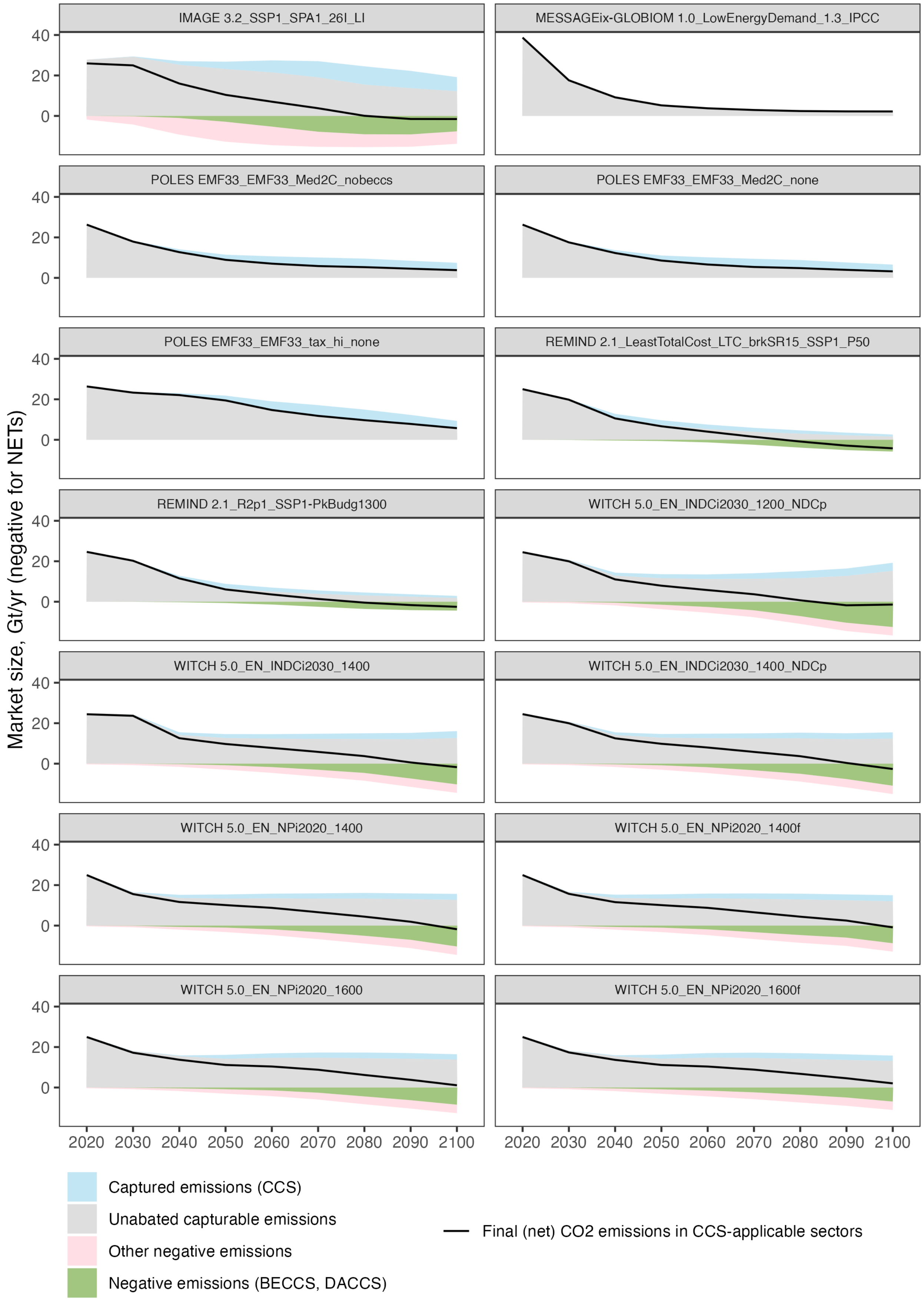

Fig. S9 | Formative phase deployment (until 2030) of mitigation pathways [2] consistent with our analytical approach to project feasible deployment of policy-driven technologies (i.e. "vetted", Methods). IMP-LED pathway is not displayed in this figure as it does not project any CCS capacity.

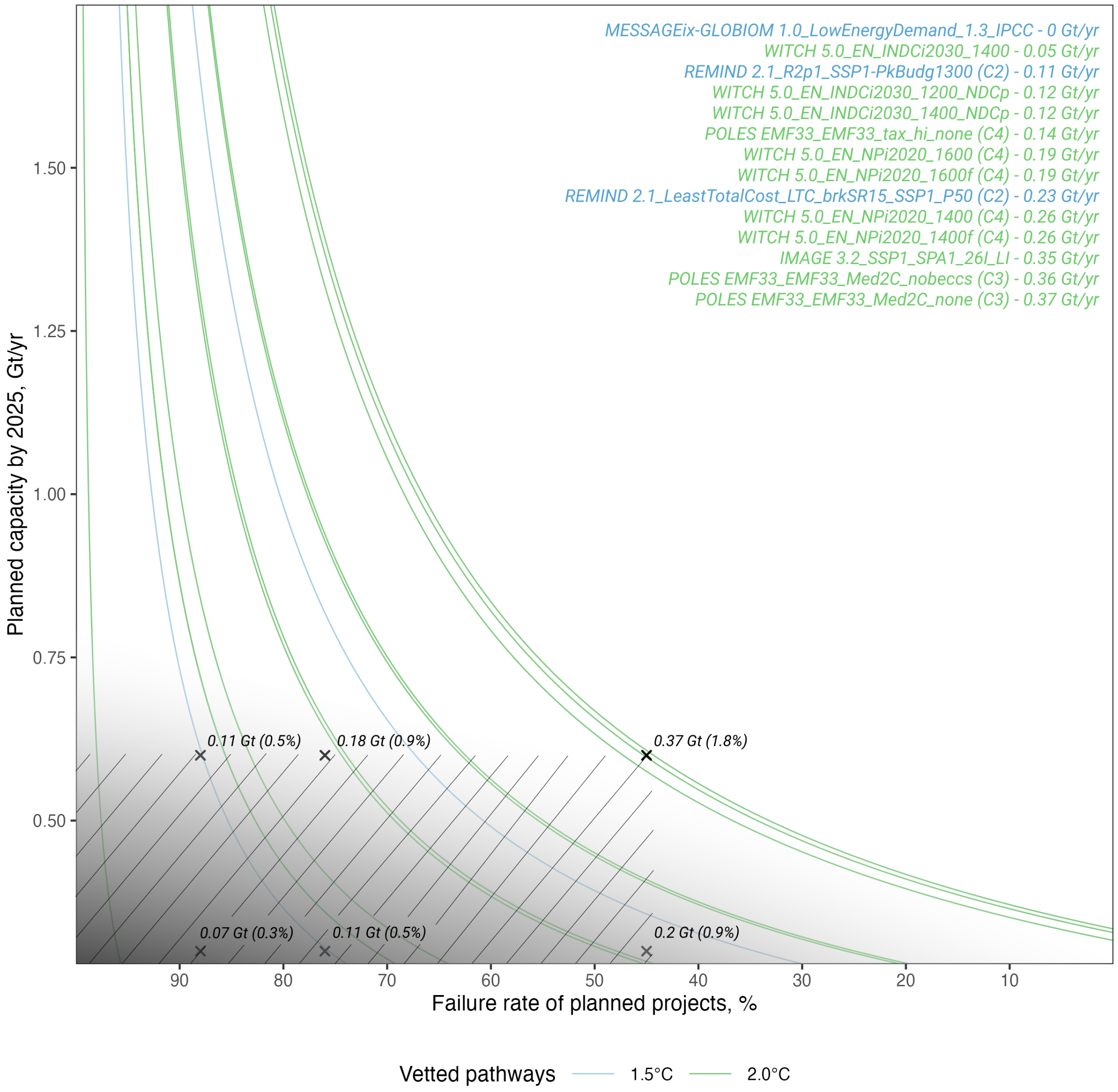

Fig. S10 | Acceleration phase growth of mitigation pathways [2] consistent with our analytical approach to project feasible deployment of policy-driven technologies (i.e. "vetted", Methods).

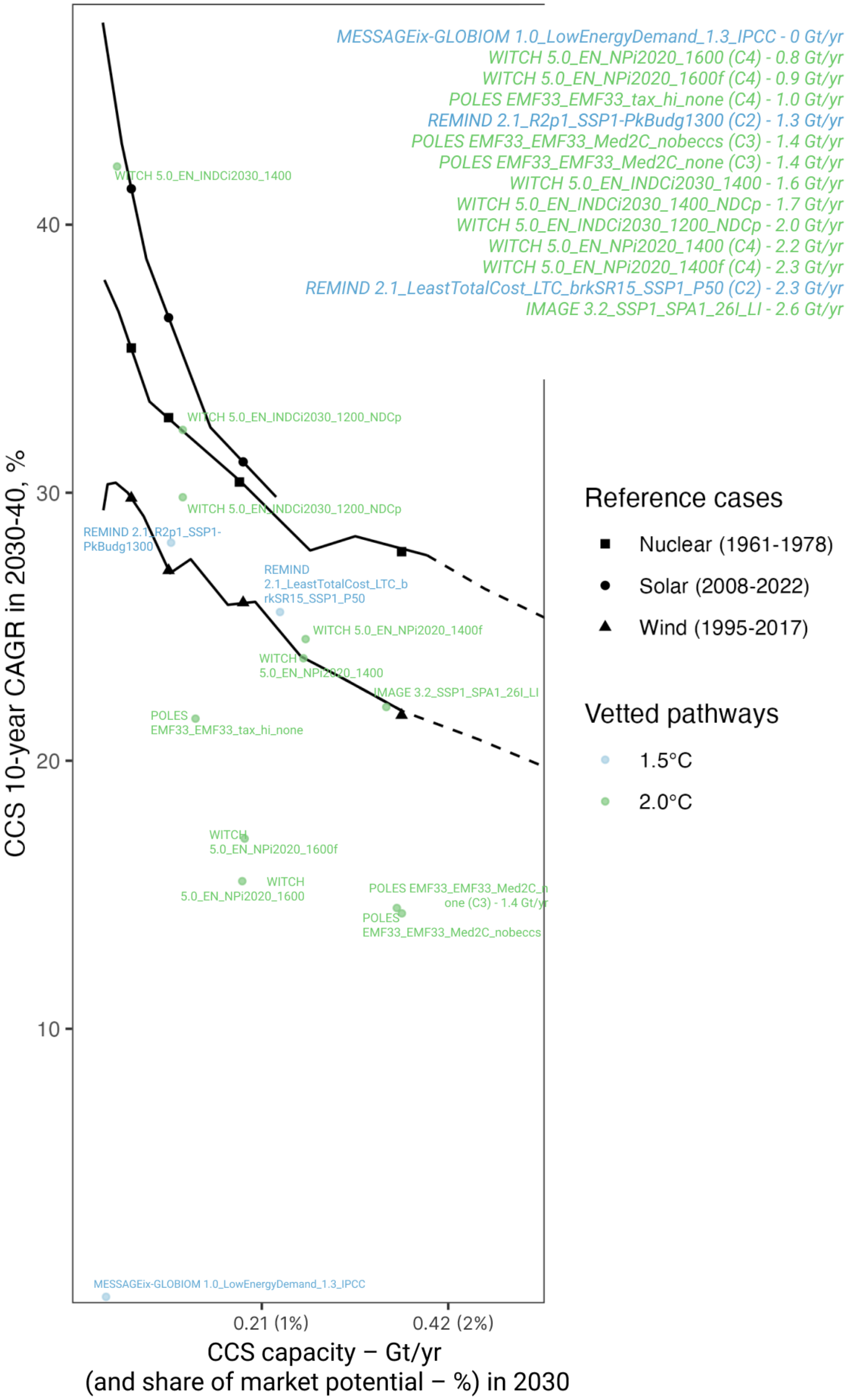

Fig. S11 | Maximum growth rates of mitigation pathways [2] consistent with our analytical approach to the feasibility of policy-driven technologies' deployment (i.e. "vetted", Methods). IMP-LED pathway is not displayed in this figure as it does not project any CCS capacity and hence does not have $TMax$.

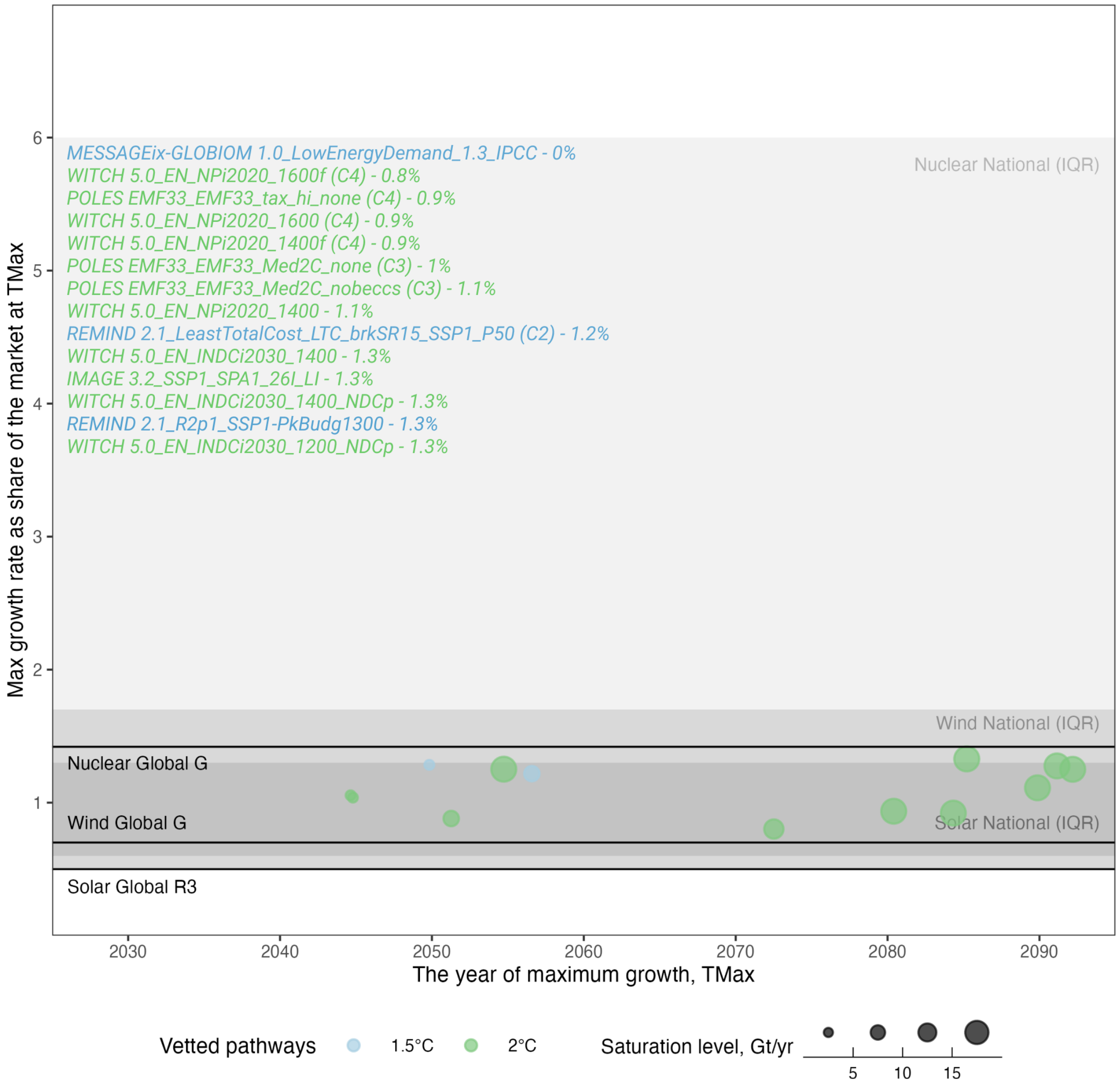

2 Supplementary Tables

Table S1 | Total planned CCS capacity before and after the start of the second wave of CCS interest and historical failure rates of CCS projects by subsector. "Sector" represents IPCC Sector categories, whereas "Subsector" distinguishes between more specific types of CCS applications (Methods, Fig. 2, Extended Data Fig. 1, Supplementary Table 3). BECCS – Bioenergy with Carbon Capture and Storage; DACCS – Direct Air Capture and Storage; NGP – Natural Gas Processing. Plans are divided in two periods – from 1972-2017 and from 2018-2022 since 2018 is the year the second wave of CCS interest started (Fig. 2). "Other" combines CCS applications with a small number of planned projects (N<5 for each application).

Sector	Subsector	CCS Plans 1972-2017			CCS Plans 2018-2022	
		Failure rate	N	Mt/yr	N	Mt/yr
Fossil Electricity	Natural Gas Power	100%	9	9,5	16	40,5
	Coal Power	98%	78	161,0	15	33,5
BECCS Electricity	Bioenergy	100%	2	2,0	6	11,5
	Waste	100%	1	0,2	7	13,8
Industry: Process	Cement	100%	1	1,0	14	14,2
Fossil Industry	Hydrogen (Coal)	91%	12	34,5	2	2,2
	Hydrogen (NG)	66%	11	8,4	50	83,3
BECCS Industry	Ethanol	70%	7	3,8	41	13,5
NGP	Natural Gas Processing	40%	20	33,2	26	48,9
DACCS	DACCS	NA	0	0,0	11	11,6
Other (N<5 in both waves)		80%	12	16,1	11	27,8
**Total**		**88%**	**153**	**269,7**	**199**	**300,8**

Table S2 | Publicly available CSS project databases

Main Sources	Details	Type	URL	Activity
Carbon Capture and Sequestration Technology Program - MIT	Mostly commercial-scale projects	Online database	https://sequestration.mit.edu/	Inactive
National Energy technology Laboratory	Commercial-scale projects, limited information on capacity (mostly in MW), sector and storage type, and time	Offline database	https://netl.doe.gov/coal/carbon-storage/worldwide-ccs-database	Active
ZeroCO2.no	Mostly commercial-scale projects	Online/Offline database	http://www.zeroco2.no/projects/list-projects	Inactive
Global CCS Institute	Only operational and planned commercial-scale projects	Online database + Annual reports	https://co2re.co/	Active
Program on Energy and Sustainable Development at Stanford University	Announced and operational projects as of 2008	Publication	https://www.readcube.com/articles/10.2139%2Fssrn.1400118	Inactive
International Association of Oil and Gas producers	Recent projects in the US, Europe, and MEA	Report	https://32zn56499nov99m251h4e9t8-wpengine.netdna-ssl.com/bookstore/wp-content/uploads/sites/2/2021/03/Global-CCS-Projects-Map.pdf	Active
Scottish Carbon Capture and Storage database	Various projects, unclear about the frequency of updates	Online database	https://www.sccs.org.uk/expertise/global-ccs-map	Unclear
Zero Emission Platform (ZEP)	Only EU projects, unclear about the frequency of updates	Online database	https://zeroemissionsplatform.eu/about-ccs-ccu/css-ccu-projects/	Unclear
IEA CCUS Projects Database	Only operational and planned commercial-scale projects	Online/Offline database	https://www.iea.org/data-and-statistics/data-tools/ccus-projects-explorer	Active
Clean Air Task Force Interactive Map	Recent projects in the US, Europe, and MEA	Offline database	https://docs.google.com/spreadsheets/d/115hsADg3ymy3lKBy4PBQRXz_MBknptqlRtlfuv79XV8/edit#gid=1540463113	Active

Table S3 | Definitions of sectors in CCS Projects Database used in this study

Sector	Subsectors	IPCC Variable	Definition
Fossil Electricity	Coal Power, Natural Gas Power	Carbon Sequestration|CCS|Fossil|Energy|Supply|Electricity	total carbon dioxide emissions captured from fossil fuel use in electricity production (part of IPCC category 1A1a) and stored in geological deposits (e.g. in depleted oil and gas fields, unmined coal seams, saline aquifers) and the deep ocean, stored amounts should be reported as positive numbers
Fossil Industry	Hydrogen (Coal), Hydrogen (Oil), Hydrogen (NG), CTL ("coal-to-liquids"), Ethylene	Carbon Sequestration|CCS|Fossil|Energy|Demand|Industry + Carbon Sequestration|CCS|Fossil|Energy|Supply (excl. Electricity)	total carbon dioxide emissions captured from fossil fuel use outside of electricity production and stored in geological deposits (e.g. in depleted oil and gas fields, unmined coal seams, saline aquifers) and the deep ocean, stored amounts should be reported as positive numbers
BECCS Electricity	Bioenergy, Waste	Carbon Sequestration|CCS|Biomass|Energy|Supply|Electricity	total carbon dioxide emissions captured from bioenergy use in electricity production (part of IPCC category 1A1a) and stored in geological deposits (e.g. in depleted oil and gas fields, unmined coal seams, saline aquifers) and the deep ocean, stored amounts should be reported as positive numbers
BECCS Industry	Ethanol, Pulp and Paper	Carbon Sequestration|CCS|Fossil|Energy|Demand|Industry + Carbon Sequestration|CCS|Fossil|Energy|Supply (excl. Electricity)	total carbon dioxide emissions captured from bioenergy use outside of electricity production and stored in geological deposits (e.g. in depleted oil and gas fields, unmined coal seams, saline aquifers) and the deep ocean, stored amounts should be reported as positive numbers
Industry: Process	Cement, Iron and Steel	Carbon Sequestration|CCS|Industrial Processes	total carbon dioxide emissions captured from industrial processes (e.g., cement production, but not from fossil fuel burning) use and stored in geological deposits (e.g. in depleted oil and gas fields, unmined coal seams, saline aquifers) and the deep ocean, stored amounts should be reported as positive numbers
NGP	Natural Gas Processing		fugitive emissions captured from the extraction and processing of the natural gas prior to its shipment and stored in geological deposits (e.g. in depleted oil and gas fields, unmined coal seams, saline aquifers) and the deep ocean
DACCS	Direct Air Capture	Carbon Sequestration|Direct Air Capture	total carbon dioxide sequestered through direct air capture

Table S4 | Definitions of regions in CCS Projects Database used in this study

Region	Description	Countries
R10NORTH_AM	countries of North America; primarily the United States of America and Canada	Canada, Guam, United States of America
R10EUROPE	countries of Eastern and Western Europe (i.e., the EU28), can include Turkey	Austria, Belgium, Croatia, Denmark, France, Finland, Spain, Sweden, Germany, Greece, Iceland, Ireland, Italy, Luxembourg, Netherlands, Norway, Portugal, Switzerland, Turkey, United Kingdom
R10PAC_OECD	countries of the Pacific OECD	Australia, Japan, New Caledonia, New Zealand, Samoa, Solomon Islands, Vanuatu
R10REF_ECON	countries from the Reforming Economies of Eastern Europe and the Former Soviet Union; primarily Russia	Armenia, Azerbaijan, Belarus, Georgia, Kazakhstan, Kyrgyzstan, Republic of Moldova, Russian Federation, Tajikistan, Turkmenistan, Ukraine, Uzbekistan
R10CHINA+	countries of centrally-planned Asia; primarily China	China (incl. Hong Kong), Cambodia, Korea (DPR), Laos (PDR), Mongolia, Viet Nam
R10INDIA+	countries of South Asia; primarily India	India, Afghanistan, Bangladesh, Bhutan, Maldives, Nepal, Pakistan, Sri Lanka,
R10REST_ASIA	other countries of Asia	(in not in India+/China+) Afghanistan, Bangladesh, Bhutan, Fiji, Maldives, Nepal, Pakistan, Sri Lanka, Cambodia, Korea (DPR), Laos (PDR), Mongolia, Viet Nam
R10AFRICA	countries of Sub-Saharan Africa	Angola, Benin, Botswana, British Indian Ocean Territory, Burkina Faso, Burundi, Cameroon, Cape Verde, Central African Republic, Chad, Comoros, Cote d'Ivoire, Congo, Djibouti, Equatorial Guinea, Eritrea, Ethiopia, Gabon, Gambia, Ghana, Guinea, Guinea-Bissau, Kenya, Lesotho, Liberia, Madagascar, Malawi, Mali, Mauritania, Mauritius, Mozambique, Namibia, Niger, Nigeria, Reunion, Rwanda, Sao Tome and Principe, Senegal, Seychelles, Sierra Leone, Somalia, South Africa, Saint Helena, Swaziland, Tanzania, Togo, Uganda, Zaire, Zambia, Zimbabwe
R10MIDDLE_EAST	countries of the Middle East; Iran, Iraq, Israel, Saudi Arabia, Qatar, etc.	Iraq, Iran (Islamic Republic), Israel, Kuwait, Lebanon, Oman, Qatar, Saudi Arabia, United Arab Emirates
R10LATIN_AM	countries of Latin America and the Caribbean	Argentina, Bahamas, Barbados, Belize, Bolivia, Brazil, Chile, Colombia, Costa Rica, Cuba, Dominican Republic, Ecuador, El Salvador, Guadeloupe, Guatemala, Guyana, Haiti, Honduras, Jamaica, Martinique, Mexico, Netherlands Antilles, Nicaragua, Panama, Paraguay, Peru, Puerto Rico, Suriname, Trinidad and Tobago, Uruguay, Venezuela
R10ROWO	Rest of the World - to be used only if decent match with the 10 regions can otherwise not be achieved	

Table S5 | Failure rates of large-scale technologies globally, calculated from ref. [5, 6]. The range of reference cases for failure rate of CCS projects are highlighted with grey. Failure rates of other technologies are calculated as a share of historically planned capacity that was not realised (as of 2013 and 2022). Emerging solar includes concentrated solar power (CSP), floating solar, and other non-photovoltaic solar power projects.

Technology	Failure rate
CCS Overall (2022)	88%
CCS Subsector-adjusted (2022)	76%
Nuclear (US, 1972-1982)	45%
Offshore floating wind (2022)	92%
Emerging solar (2013)	75%
Emerging solar (2022)	66%
Offshore wind (2013)	60%
Nuclear (2022)	42%

Table S6 | CCS capacity in 2030 based on a range of assumptions about planned projects and their failure rates.

CCS Capacity in 2030, Gt/yr	88% failure	76% failure	45% failure
Current plans	0.07	0.11	0.2
Doubling	0.11	0.18	0.37

Table S7 | Sensitivity of long-term outcomes of CCS deployment to different combinations of assumptions about the formative, acceleration, and stable growth phases in the existing IPCC AR6 scenario ensemble. The first (upper) panel of this compound table shows the cumulative CO_2 capture and storage between 2030 and 2070 (Gt) in the IPCC AR6 scenario ensemble [2]. The remaining panels of the table illustrate how different combinations of assumptions about growth metrics (Table 1) in the formative, acceleration (first two columns), and stable growth (remaining columns) phases affect this outcome value (median, Q1, Q3, 95th percentile, and maximum) and the number of pathways that satisfy these assumptions (N), for 1.5°C (second panel), 2°C pathways (third panel), and combined (fourth panel).

	temp	N	median	Q1	Q3	95th	max
Unconstrained (all pathways)	1.5°C	218	286	208	485	789	981
	2°C	423	220	172	313	569	915
	both	641	238	183	390	685	981

			Stable growth phase: FGD G2022 (Optimistic)						Stable growth phase: G2022 (Central)						Stable growth phase: FGD GTMax (Optimistic)						Stable growth phase: GTMax (Central)					
**Formative phase**	**Acceleration phase**	**temp**	**N**	**median**	**Q1**	**Q3**	**95th**	**max**	**N**	**median**	**Q1**	**Q3**	**95th**	**max**	**N**	**median**	**Q1**	**Q3**	**95th**	**max**	**N**	**median**	**Q1**	**Q3**	**95th**	**max**
**2030-failure 0% plans doubling**	*2040-FGD*	1.5°C	70	245	202	286	392	506	21	149	113	202	208	208	65	251	202	287	430	807	5					113
**2030-failure 0% plans doubling**	*2040 - nuclear + solar*	1.5°C	51	251	202	289	413	506	13	149	113	201	204	206	50	247	202	286	472	807	4					113
**2030-failure 0% plans doubling**	*2040-wind*	1.5°C	22	205	196	267	313	341	8					206	22	205	196	267	313	341	2					83
**2030-central**	*2040-FGD*	1.5°C	38	223	184	273	381	396	16	130	113	204	208	208	35	236	162	281	389	807	4					113
**2030 - central**	*2040-nuclear+solar*	1.5°C	19	219	162	253	325	351	8					206	20	223	176	258	374	807	3					113
**2030 - central**	*2040 - wind*	1.5°C	7					271	3					206	7					271	1					0
**2030-failure 88% current plans**	*2040-FGD*	1.5°C	19	208	128	265	355	387	11	128	120	194	208	208	16	234	128	318	492	807	2					111
**2030-failure 88% current plans**	*2040-nuclear + solar*	1.5°C	7					351	3					132	8					807	1					0
**2030-failure 88% current plans**	*2040-wind*	1.5°C	3					255	1					0	3					255	1					0

			Stable growth phase: FGD G2022 (Optimistic)						Stable growth phase: G2022 (Central)						Stable growth phase: FGD GTMax (Optimistic)						Stable growth phase: GTMax (Central)					
**Formative phase**	**Acceleration phase**	**temp**	**N**	**median**	**Q1**	**Q3**	**95th**	**max**	**N**	**median**	**Q1**	**Q3**	**95th**	**max**	**N**	**median**	**Q1**	**Q3**	**95th**	**max**	**N**	**median**	**Q1**	**Q3**	**95th**	**max**
**2030-failure 0% plans doubling**	*2040-FGD*	2°C	284	193	161	235	400	556	94	168	145	187	213	250	245	188	157	231	406	556	19	130	105	150	179	241
**2030-failure 0% plans doubling**	*2040 - nuclear + solar*	2°C	241	191	160	235	416	556	76	158	130	178	201	250	219	185	158	225	417	556	17	128	105	154	186	241
**2030-failure 0% plans doubling**	*2040-wind*	2°C	147	191	165	220	313	458	44	155	115	181	193	205	132	187	155	210	325	458	13	128	102	154	200	241
**2030-central**	*2040-FGD*	2°C	225	187	156	222	273	468	76	163	142	187	212	222	193	182	150	214	273	429	13	115	105	138	182	241
**2030 - central**	*2040-nuclear+solar*	2°C	182	183	153	211	280	468	58	151	127	171	193	222	167	181	150	214	273	429	11	113	103	129	191	241
**2030 - central**	*2040 - wind*	2°C	106	189	153	211	266	341	27	146	108	173	187	191	96	185	151	202	264	341	7					241
**2030-failure 88% current plans**	*2040-FGD*	2°C	123	183	155	206	260	429	35	169	146	196	222	222	105	177	149	208	263	429	3					141
**2030-failure 88% current plans**	*2040-nuclear + solar*	2°C	85	174	152	201	252	429	17	146	113	160	167	169	84	175	153	201	252	429	1					113
**2030-failure 88% current plans**	*2040-wind*	2°C	45	186	146	201	237	280	4					110	44	187	152	201	238	280	0					

			Stable growth phase: FGD G2022 (Optimistic)						Stable growth phase: G2022 (Central)						Stable growth phase: FGD GTMax (Optimistic)						Stable growth phase: GTMax (Central)					
**Formative phase**	**Acceleration phase**	**temp**	**N**	**median**	**Q1**	**Q3**	**95th**	**max**	**N**	**median**	**Q1**	**Q3**	**95th**	**max**	**N**	**median**	**Q1**	**Q3**	**95th**	**max**	**N**	**median**	**Q1**	**Q3**	**95th**	**max**
**2030-failure 0% plans doubling**	*2040-FGD*	both	354	200	167	250	398	556	115	167	130	191	208	250	310	197	160	250	412	807	24	114	102	143	172	241
**2030-failure 0% plans doubling**	*2040 - nuclear + solar*	both	292	198	163	250	419	556	89	157	128	181	204	250	269	195	160	243	424	807	21	113	101	145	172	241
**2030-failure 0% plans doubling**	*2040-wind*	both	169	194	169	223	314	458	52	159	115	183	201	206	154	191	167	214	324	458	15	106	100	154	193	241
**2030-central**	*2040-FGD*	both	263	191	157	226	290	468	92	161	128	188	208	222	228	187	150	228	305	807	17	113	102	130	162	241
**2030 - central**	*2040-nuclear+solar*	both	201	186	153	219	284	468	66	150	126	171	201	222	187	183	151	211	283	807	14	109	101	125	177	241
**2030 - central**	*2040 - wind*	both	113	191	154	214	266	341	30	148	107	175	191	206	103	187	152	207	266	341	8					241
**2030-failure 88% current plans**	*2040-FGD*	both	142	183	153	209	276	429	46	165	130	197	218	222	121	179	147	211	284	807	5					141
**2030-failure 88% current plans**	*2040-nuclear + solar*	both	92	175	149	202	269	429	20	144	112	159	167	169	92	176	152	202	282	807	2					113
**2030-failure 88% current plans**	*2040-wind*	both	48	187	145	202	247	280	5					110	47	188	150	202	248	280	1					0

Table S8 | Sensitivity of long-term outcomes of CCS deployment to different combinations of assumptions about the formative, acceleration, and stable growth phases in the existing IPCC AR6 scenario ensemble. The first (upper) panel of this compound table shows the cumulative CO_2 capture and storage between 2030 and 2100 (Gt) in the IPCC AR6 scenario ensemble [2]. The remaining panels of the table illustrate how different combinations of assumptions about growth metrics (Table 1) in the formative, acceleration (first two columns), and stable growth (remaining columns) phases affect this outcome value (median, Q1, Q3, 95th percentile, and maximum) and the number of pathways that satisfy these assumptions (N), for 1.5°C (second panel), 2°C pathways (third panel), and combined (fourth panel).

	temp	N	median	Q1	Q3	95th	max
Unconstrained (all pathways)	1.5°C	218	724	532	1018	1514	2247
	2°C	423	644	491	866	1330	2109
	both	641	682	510	926	1428	2247

			Stable growth phase: FGD G2022 (Optimistic)						Stable growth phase: G2022 (Central)						Stable growth phase: FGD GTMax (Optimistic)						Stable growth phase: GTMax (Central)					
**Formative phase**	**Acceleration phase**	**temp**	**N**	**median**	**Q1**	**Q3**	**95th**	**max**	**N**	**median**	**Q1**	**Q3**	**95th**	**max**	**N**	**median**	**Q1**	**Q3**	**95th**	**max**	**N**	**median**	**Q1**	**Q3**	**95th**	**max**
**2030-failure 0% plans doubling**	*2040-FGD*	1.5°C	70	657	486	829	1227	1515	21	419	389	519	605	605	65	678	479	832	1398	2247	5					389
**2030-failure 0% plans doubling**	*2040 - nuclear + solar*	1.5°C	51	662	487	833	1381	1515	13	419	290	516	605	605	50	662	486	834	1420	2247	4					290
**2030-failure 0% plans doubling**	*2040-wind*	1.5°C	22	543	480	809	942	1015	8					605	22	543	480	809	942	1015	2					179
**2030-central**	*2040-FGD*	1.5°C	38	627	473	782	1066	1515	16	417	394	576	605	605	35	709	440	841	1402	2247	4					389
**2030 - central**	*2040-nuclear+solar*	1.5°C	19	605	468	853	1369	1515	8					605	20	628	492	905	1551	2247	3					290
**2030 - central**	*2040 - wind*	1.5°C	7					1015	3					605	7					1015	1					0
**2030-failure 88% current plans**	*2040-FGD*	1.5°C	19	578	408	831	1369	1515	11	415	398	544	581	584	16	743	401	923	1698	2247	2					389
**2030-failure 88% current plans**	*2040-nuclear + solar*	1.5°C	7					1515	3					419	8					2247	1					0
**2030-failure 88% current plans**	*2040-wind*	1.5°C	3					1015	1					0	3					1015	1					0

			Stable growth phase: FGD G2022 (Optimistic)						Stable growth phase: G2022 (Central)						Stable growth phase: FGD GTMax (Optimistic)						Stable growth phase: GTMax (Central)					
**Formative phase**	**Acceleration phase**	**temp**	**N**	**median**	**Q1**	**Q3**	**95th**	**max**	**N**	**median**	**Q1**	**Q3**	**95th**	**max**	**N**	**median**	**Q1**	**Q3**	**95th**	**max**	**N**	**median**	**Q1**	**Q3**	**95th**	**max**
**2030-failure 0% plans doubling**	*2040-FGD*	2°C	284	583	457	744	1045	1498	94	425	396	542	604	621	245	566	452	766	1080	1498	19	405	335	510	584	645
**2030-failure 0% plans doubling**	*2040 - nuclear + solar*	2°C	241	568	445	757	1103	1498	76	414	382	449	584	621	219	561	440	766	1109	1498	17	402	320	466	591	645
**2030-failure 0% plans doubling**	*2040-wind*	2°C	147	577	453	730	1033	1242	44	413	339	454	525	600	132	566	456	735	1059	1242	13	405	247	466	604	645
**2030-central**	*2040-FGD*	2°C	225	567	456	713	865	1182	76	425	394	551	612	621	193	547	443	727	868	1182	13	392	320	510	571	645
**2030 - central**	*2040-nuclear+solar*	2°C	182	552	436	714	917	1182	58	411	372	434	583	621	167	535	433	713	905	1182	11	385	284	403	578	645
**2030 - central**	*2040 - wind*	2°C	106	594	462	731	855	1182	27	372	328	436	478	535	96	565	460	735	857	1182	7					645
**2030-failure 88% current plans**	*2040-FGD*	2°C	123	583	486	753	919	1182	35	473	422	568	613	618	105	598	461	766	931	1182	3					522
**2030-failure 88% current plans**	*2040-nuclear + solar*	2°C	85	558	454	766	1009	1182	17	420	392	432	435	438	84	561	455	766	1014	1182	1					392
**2030-failure 88% current plans**	*2040-wind*	2°C	45	713	512	771	855	1182	4					328	44	713	524	772	855	1182	0					

			Stable growth phase: FGD G2022 (Optimistic)						Stable growth phase: G2022 (Central)						Stable growth phase: FGD GTMax (Optimistic)						Stable growth phase: GTMax (Central)					
**Formative phase**	**Acceleration phase**	**temp**	**N**	**median**	**Q1**	**Q3**	**95th**	**max**	**N**	**median**	**Q1**	**Q3**	**95th**	**max**	**N**	**median**	**Q1**	**Q3**	**95th**	**max**	**N**	**median**	**Q1**	**Q3**	**95th**	**max**
**2030-failure 0% plans doubling**	*2040-FGD*	both	354	592	461	763	1078	1515	115	424	395	540	605	621	310	599	455	777	1132	2247	24	390	249	477	576	645
**2030-failure 0% plans doubling**	*2040 - nuclear + solar*	both	292	590	452	768	1138	1515	89	415	372	457	588	621	269	568	448	781	1181	2247	21	385	247	412	577	645
**2030-failure 0% plans doubling**	*2040-wind*	both	169	568	457	735	1023	1242	52	414	339	470	534	605	154	566	458	741	1031	1242	15	349	217	439	598	645
**2030-central**	*2040-FGD*	both	263	575	456	729	917	1515	92	423	394	554	608	621	228	564	442	744	930	2247	17	385	250	405	546	645
**2030 - central**	*2040-nuclear+solar*	both	201	559	435	727	944	1515	66	411	366	437	589	621	187	541	433	731	994	2247	14	335	247	399	557	645
**2030 - central**	*2040 - wind*	both	113	598	468	735	877	1034	30	384	328	452	526	621	103	567	464	745	890	1182	8					645
**2030-failure 88% current plans**	*2040-FGD*	both	142	581	465	763	1011	1515	46	436	415	569	612	618	121	599	456	777	1037	2247	5					522
**2030-failure 88% current plans**	*2040-nuclear + solar*	both	92	561	450	772	1042	1515	20	418	376	429	435	438	92	580	453	778	1107	2247	2					392
**2030-failure 88% current plans**	*2040-wind*	both	48	713	510	778	972	1182	5					328	47	714	520	779	978	1182	1					0

Table S9 | Capital costs of projects involving CCS [7–10] and selected reference cases [11–14]. For CCS, we use successful projects or projects under construction as benchmarks. For reference cases, capital costs are collected from early deployment periods (formative or acceleration phase): 1970's for nuclear power, 2000 for wind, 2010 for solar, and 1975 for FGD. Costs are then adjusted to US$2024 using CPI Inflation Calculator [15] and then adjusted for project size to provide approximate cost estimates for 240 MW for projects in the power sector and 1 MtCO2/yr in the industry sector.

	Project capital cost (for power 240 MW and for industry* 1 Mt/year)	Note on reference project(s)	Reference
**CCS capital costs**			
**CCS Power**	1.3 US$2024 billion	Petra Nova project (coal power): 4,200 US$2014/kW, 1 Mt/yr capacity	EIA 2017
**CCS Industry I** (cement, iron & steel, waste-to-energy)	2.0 US$2024 billion	Northern Lights project (cement and waste-to-energy): 0.8 Mt/yr to be captured from a cement (CAPEX 0.5 US$ billion) and waste-to-energy plant (CAPEX 0.4 US$ billion), 1.5 Mt/yr storage capacity (US$ 1 billion). In development; costs might be incomplete.	Bellona 2020
**CCS Industry II** (ethanol, ammonia, natural gas processing, ethylene)	0.4 US$2024 billion**	Illinois Industrial CCS project (ethanol): 1 Mt/yr. Costs are adjusted from US$2017 - the year when project became operational.	IEA Bioenergy 2023
**DACCS**	>2 US$2024 billion	STRATOS project: 0.5 Mt/yr. In development; costs might be incomplete.	Carbon Credits 2024
**Reference technology capital costs**			
**Nuclear**	0.4 US$2024 billion	~1000 US$2004/kW based on construction costs of projects completed in the US (ca. 1970-1975) and France (ca. 1975-1980). These are one of the lowest historical costs of the technology.	Grubler 2010
**Wind**	0.6 US$2024 billion	~1700 US$2010/kW installed project capital cost based on projects in the US and Denmark (2000)	NREL 2012
**Solar**	1.4 US$2024 billion	~4100 US$2010/kW installed system price based on utility-scale PV systems in the US (2010)	IRENA 2012
**FGD**	0.1 US$2024 billion	~250 US$1997/kW – project capital cost of FGD units (1976). These are one of the highest historical costs of the technology.	Rubin et al. 2007

*- a 240 MW Petra Nova project was planned to capture 1 Mt/yr. For a reasonable comparison of costs, we therefore scale the costs of industrial CCS projects to a similar capture and storage capacity.
**- ICCS project has been integrated with a large-scale geologic test ("Illinois Basin Decatur Project"). Costs of both projects are considered in this table. ICCS-only project cost is 0.3 US$2024 billion.

Table S10 | Median and IQR of CCS capacity variables in IPCC AR5 [16], SR1.5 [17–19], AR6, and AR6 IMP [1, 2] pathways for reaching 1.5°C degree warming by the end of the century. "Capacity 20 years after publication" is calculated using linear extrapolation between decadal values in the IPCC pathways: for AR5 (2014), this corresponds to the CCS capacity achieved by 2034, for SR1.5 (2018) – 2038, and for AR6 (2022) – 2042.

Ensemble/IMP	t°C	Capacity 20 years after publication	G (Gt/yr/yr)	L (Gt/yr)	TMax*	dT
			Average values between Gmp and Log			
IPCC Scenario ensembles						
AR5 (2014)	1.5°C	4.7 (2.6-6.5)	0.7 (0.5-0.9)	22 (17-33)	31 (23-37)	41 (29-53)
SR1.5 (2018)	1.5°C	4.6 (3.0-6.9)	0.6 (0.4-0.8)	17 (13-25)	25 (21-32)	33 (25-47)
AR6 (2022)	1.5°C	4.5 (3.1-8.4)	0.5 (0.3-0.8)	17 (12-23)	25 (18-37)	36 (28-60)
SR1.5 (2018)	2.0°C	2.1 (0.3-4.0)	0.4 (0.2-0.7)	17 (8-25)	32 (23-43)	38 (28-55)
AR6 (2022)	2.0°C	2.9 (2.0-4.8)	0.4 (0.3-0.6)	17 (12-23)	32 (26-38)	41 (34-58)
AR6 (2022)	2.5°C	0.6 (0.3-2.1)	0.4 (0.2-0.5)	16 (11-24)	45 (38-52)	47 (36-60)
Illustrative Mitigation Pathways						
AR6-SP	1.5°C	1.8	0.1	3	18	39
AR6-Ren	1.5°C	3.2	0.2	3	12	18
AR6-LED	1.5°C	0	0	0	0	0
SR1.5-S1	1.5°C	2.6	0.4	8	23	25
SR1.5-S2	1.5°C	4.9	0.3	13	25	51
SR1.5-S5	1.5°C	5.9	1	22	25	23
SR1.5-LED	1.5°C	0	0	0	0	0
AR6-GS	2.0°C	2.1	0.2	12	41	60
AR6-Neg	2.0°C	3.7	0.3	11	23	36

**Number of years until TMax from the year of ensemble publication*

Table S11 | Summary of mitigation pathways [2] consistent with our analytical approach to the feasibility of policy-driven technologies' deployment (i.e. "vetted", Supplementary Note 6). Vetted pathways are grouped by their deployment parameters in the three phases of growth into three groups ("Vetting group") described in Supplementary Note 6.

			Formative		Acceleration	Stable Growth			
Model_Scenario	Temperaure	Vetting group	CCS Capacity 2030, Mt/yr	CCS Market Share 2030, %	10-yr CAGR 2030-40,%	Maximum annual growth G, Mt/yr/yr	G_Tmax, %	TMax, yr	Saturation L, Gt
REMIND 2.1_LeastTotalCost_LTC_brkSR15_SSP1_P50	1.5°C	1	231	1,1%	26%	113	1,2%	2057	8
REMIND 2.1_R2p1_SSP1-PkBudg1300	1.5°C	1	108	0,5%	28%	126	1,3%	2050	5
POLES EMF33_EMF33_Med2C_nobeccs	2.0°C	1	363	1,7%	15%	135	1,1%	2045	4
POLES EMF33_EMF33_tax_hi_none	2.0°C	1	136	0,6%	22%	189	0,9%	2051	6
POLES EMF33_EMF33_Med2C_none	2.0°C	1	369	1,8%	14%	128	1,0%	2045	4
IMAGE 3.2_SSP1_SPA1_26I_LI	2.0°C	2	351	1,7%	22%	390	1,3%	2055	17
WITCH 5.0_EN_INDCi2030_1200_NDCp	2.0°C	2	121	0,6%	32%	323	1,3%	2085	29
WITCH 5.0_EN_INDCi2030_1400	2.0°C	2	47	0,2%	42%	292	1,3%	2092	22
WITCH 5.0_EN_INDCi2030_1400_NDCp	2.0°C	2	121	0,6%	30%	294	1,3%	2091	22
WITCH 5.0_EN_NPi2020_1400	2.0°C	2	258	1,2%	24%	254	1,1%	2090	20
WITCH 5.0_EN_NPi2020_1400f	2.0°C	2	260	1,2%	25%	192	0,9%	2084	20
WITCH 5.0_EN_NPi2020_1600	2.0°C	2	189	0,9%	16%	203	0,9%	2080	17
WITCH 5.0_EN_NPi2020_1600f	2.0°C	2	191	0,9%	17%	159	0,8%	2073	12
MESSAGEix-GLOBIOM 1.0_LowEnergyDemand_1.3_IPCC	1.5°C	3	0	0%	0%	0	0%	NA	0

3 Supplementary Notes

Supplementary Note 1: CCS Projects Database

To study historical failure rates of CCS projects, we built a dataset of completed, failed, and currently planned commercial (at least 0.1 Mt/yr capacity) CCS projects starting from 1972, the completion year of the first integrated CCS project (Terrell natural gas processing plant). For each project, we coded: the capture rate; project announcement and completion year; facility status (e.g. active, failed, planned); facility operation start and end years; CO_2 storage type (e.g. enhanced oil recovery or dedicated geological storage); sectoral and subsectoral application, country; and region (Supplementary Tables 3-4).

For the database, we documented both the first and second wave of CCS interest. For the first wave, we reviewed annual reports from the Global CCS Institute (GCCSI) which contain entries on CCS projects under development, as well as past and existing databases listing planned CCS projects at different points in time ([20–23], full list in Supplementary Table 2). We also cross-checked our entries for the first wave and captured the second wave from the recently published (March 2023) IEA CCUS Projects Database [24] which focuses on planned projects during the second wave of CCS interest (from app. 2018).

To identify projects that might have been missed in the above-mentioned sources and to fill in data gaps concerning all projects, we conducted a systematic Google search in November and December 2021, using the search string "[country name] + CCS projects" and "[country name] + carbon capture project" reviewing results from the first three results pages for evidence of missing projects and variables. To fill any data gaps related to the project capacity, we conducted another systematic Google search in February and March 2022, using the search string "[project name]" and "[project name] + capacity". This additional search was required as some past CCS project databases reported the capacity of CCS projects in the electricity sector in megawatts instead of tons of captured CO_2. To fill in any data gaps related to the year of project announcement, completion, or termination, we used the same search as above, but this time using a "customised date range" feature of the Google search engine to track the first and last mentions of the project. When coding results from our systematic Google searches, we used company press-releases; policy documents, GCCSI and other similar reports; conference proceedings; and when none of these sources were available (rarely) news reports.

Supplementary Note 2: Flue gas desulphurisation as a technology reference case for CCS: comparison of costs and public acceptance

In this study, we use flue gas desulphurisation (FGD) – a pollution control technology without pipeline transportation and underground storage needs – to verify whether the CO_2 capture component of CCS imposes additional constraints on the feasible speed of CCS deployment (Fig. 3). In addition, we test the sensitivity of the number of pathways that meet the feasibility constraints and the amount of CO_2 they capture by 2070 and 2100 to more optimistic feasibility constraints based on the assumption that the entire CCS technological chain (capture, transportation and underground storage) can grow as fast as FGD grew historically. In this Supplementary Note, we discuss the realism of this assumption, i.e. whether FGD is a valid analogy for the entire CCS technology. We focus particularly on the two aspects critical for speed of technology deployment: costs and public acceptance.

Literature documents how both factors limit the speed of CCS deployment. The history of CCS project failures in the 2010's indicates that CCS deployment was constrained by high capital costs [25, 26]. Today even the least expensive CCS applications face difficulties because of public opposition, particularly to transportation and storage. Reference cases of policy-driven energy technologies used in this study – nuclear, wind, and solar power – have also been characterised by high capital costs similar to those of CCS (Supplementary Table 9) and public acceptance issues [27–34].

High capital costs have been identified as one of the largest barriers to fast CCS deployment [26, 35–39]. Carbon capture costs vary greatly depending on the sector where the technology is applied (Supplementary Table 9), with high costs prevailing in sectors with most CO_2 abatement potential. In the power sector, where CCS was primarily targeted in the first and to a large extent in the second wave of industry interest (Fig. 1A), carbon capture costs (without transportation and storage) can double capital investments required for a power plant [1] and increase operational costs due to higher energy consumption, driving the levelised cost of electricity up by 50% or more [39]. The costs are notably higher when transportation and storage are included. For example, Petra Nova project – a 240 MW coal power plant unit which was retrofitted with CCS in 2017 – required construction costs of around US$ 1 billion or 4,200 US$2014/kW, incl. pipeline construction and CO_2 storage equipment [7]. For comparison,

capital investments of such scale could have contributed to another power plant project (without CCS) of a similar size or bigger [39]. Still, the cost of Petra Nova was considered "relatively low" by the Global CCS Institute compared to Boundary Dam (completed before Petra Nova) and Kemper (failed) projects [40].

Industrial sectors such as cement, iron and steel, and hydrogen typically fall in a similar range of costs per tonne of CO_2 captured [41]. In Norway, 359 US$ million is committed to the capital costs of a 50% CO2 capture (0.4 Mt/yr) at Norcem Brevik cement plant [8]. For comparison, recent project announcements show that there are cement plants of a larger capacity that cost two time less than this project (albeit in developing countries).

The lowest costs of carbon capture are typically found in sectors like ammonia, ethylene oxide, ethanol, natural gas processing, and coal-to-liquids [41]. However, the applicability and potential of CCS in these sectors is more nuanced: natural gas processing only requires carbon 'separation' if the CO_2 content in extracted natural gas is above the natural gas pipeline standards; ammonia facilities can use CO_2 for urea production instead of transporting and storing it; ethanol has multiple CO_2 emission sources where one (fermentation) is relatively cheap and the other (boilers) is not [42]. Even though these sectors represent an early opportunity for CCS, the eventual demand for CCS in these sectors might be limited or the cost of full CCS integration might increase.

When it comes to transportation and storage, costs can also vary depending on capacity, location (onshore or offshore), distance from the CO2 source, and storage type (enhanced oil recovery or saline formations). Out of EUR 1.5 billion capital costs of the Longship project in Norway (which combines two capture plants with cumulative capacity of 0.8 Mt/yr and transportation and storage infrastructure with 1.5 Mt/yr capacity), over a half is dedicated to the Northern Lights transportation and offshore storage part of the project [8]. Thus, CO_2 transportation and storage infrastructure can contribute to significant project cost increases when compared to capture-only costs.

Public acceptance is another factor potentially constraining the growth of new technologies [43, 44]. Our central reference technologies: nuclear power and renewables – have experienced public opposition related to safety (e.g. nuclear waste) and conflicting land uses (e.g. transmission lines), that are similar to concerns about CO_2 storage and transportation. Although familiarity with and public acceptance of CCS can vary considerably [45], scholarship shows that negative public perception can lead to cost overruns, project delays and failures [46]. For example, a multi-billion CO_2 pipeline project connecting multiple ethanol plants in the US has recently been postponed due to the opposition from landowners and advocacy groups, while paying out hundreds of millions to "supportive" landowners to build on their properties. Even if a technology succeeds, the support for large-scale projects can decrease faster in areas which previously experienced project development [29].

In comparison to CCS, FGD is cheaper and less controversial. With costs lower than those of CCS by a factor of between 4 and more than 20 (Supplementary Table 9), FGD has been deployed with "command-and-control" (Large Combustion Plant Directive in the EU) and market-based mechanisms (cap-and-trade program in the US). For example, introduced in light of an increasing public concern about the health effects of sulphur pollution between the 1950's and 1970's, the US Clean Air Act and successive regulations were sufficient to enable power plants compliance with strict SO_x emission standards despite the resistance from utilities [47]. Perhaps due the nation-wide public concern [48] and no large-scale infrastructure requirements, no evidence of public opposition to FGD has ever been documented.

Thus, despite some technological similarities, there are two key differences between FGD and CCS: FGD is less capital-intensive and lacks pipeline and storage components which are likely to cause public opposition. Whereas FGD offers arguably the best evidence for the learning rates [14] and unit scaling of pollution control technologies, it can only be a realistic reference case for the carbon capture component of CCS (even in that case FGD's lower costs need to be taken into account). For CCS as a whole, reference technologies which are more similar in capital costs and public attitudes, such as nuclear and renewable energy, are more suitable reference cases.

Supplementary Note 3: Investment needs for the estimated range of CCS uptake by 2030

According to BNEF [49], global annual investment in CCS reached US$6.4 billion in 2022, more than double the previous year's value. Is this enough to bring CCS on-track for climate targets? Projecting investment needs for upscaling emerging technologies is at least as uncertain as projecting their deployment. Both projections are associated with project failures. Whereas for deployment this means a

project plan is never realised, cost management scholars [50, 51] define failures in terms of cost overruns and delays – implying that even a completed project can be considered a failure. Research shows that large (e.g. nuclear) and new project designs (e.g. floating offshore power) tend to face cost overruns more frequently [50, 51]. Given that CCS projects share many attributes with megaprojects [26] and have project-specific attributes which affect costs (e.g. CO_2 capture costs for different point sources, distance from CO_2 source to sink, sink type), estimating investment needs for CCS deployment even in the short-term (2030) involves a lot of uncertainty and thus requires advanced modelling frameworks (see e.g. ref [52]) which are beyond the scope of this study.

Nevertheless, previous modelling studies provide useful insights for defining the CCS investment gap in the near-term (2030). For instance, IEA's Sustainable Development Scenario [53] indicates an investment gap of US$160 billion to upscale CCS capacity to 0.8 Gt/yr by 2030. We estimate 0.07-0.37 Gt/yr to be the feasible range of CCS capacity, which means a rough estimate of annual investment needs in 2024-2030 for the upper limit would be around US$12 billion annually (US$1.2 billion for the lower limit). However, McCollum et al. (2018) [54] point out that the IEA's scenario-based investment projections tend to be lower than in most Integrated Assessment Models' scenarios meeting climate targets. In their intermodel comparison, four out of six 2°C pathways project average annual investments in CCS at US$15 billion in 2025-2030 (although it is unclear what CCS capacity is reached via these investments). Two remaining models show a contrasting image: one (WITCH-GLOBIOM) projects US$60 billion annual investments already in 2020-2025, whereas another (AIM/CGE) projects CCS investments to start from 2030 at US$18 billion annually. Thus, based on recent modelling studies, investments needed to bring CCS on track for the 2°C target by 2030 range between US$12-15 billion annually – at least twice as much as in 2022, but less than 1% of annual clean energy investments the same year [55]. Reaching 0.07 Gt/yr capacity would require at least 1.2-2 US$ billion annually.

Supplementary Note 4: Policy considerations for CCS deployment in the three phases of growth

Putting CCS on track for policy and climate targets and sustaining its status as a growing climate mitigation technology requires substantial and sustained improvements of policy and regulatory support for the technology. Here we reflect on how this support could evolve over the three phases of growth.

Formative phase. Today, CCS is in the formative phase and the US and Europe are leading markets. In the US, CCS deployment is incentivised through the 45Q tax credit, which was first introduced in 2008. Recent changes in 2018 (through the Bipartisan Budget Act) and 2022 (through the Inflation Reduction Act) provided better conditions for a diversified portfolio of CCS projects, mostly through a higher tax credit (from 10 US$/t to up to 180 US$/t), reduction of the minimum capacity of eligible projects, and a 2032 deadline for project construction – all of which could lead to continuous growth of CCS plans. In the European Union, CCS deployment is driven primarily by the EU Hydrogen Strategy [56], national subsidy schemes, and the EU Innovation Fund, which have already provided support for CCS projects across a number of subsectors, with a particular emphasis on blue hydrogen (40% of planned 48 Mt/yr capacity in 2022). The recent (March 2023) call by the European Commission for a 50 Mt annual storage capacity by 2030 was proposed in the Net Zero Industry Act (NZIA), which also aims to facilitate permit-granting and access to finance. The most recent policy contribution is the EU Industrial Carbon Management Strategy published in February 2024, which provides an estimate for the role of CCS in the European Union in 2040. We analyse the feasibility of these deployment plans in Supplementary Note 7 below.

As our analysis shows, decreasing the failure rate of individual projects is the key policy priority. Recent literature highlights capital costs as the key reason of past CCS project failures [26, 57]. This empirical analysis is from the first wave projects when project developers had to experiment with different business models by stacking various revenue streams and incentive packages [25]. Today, CCS receives more financial support than during the first wave of industry interest, which is reflected in the rise of industry interest above the historical peak in 2010 (Fig. 2).

However, realising this interest and bringing CCS on-track for Paris targets will also require better regulation and broader social acceptance of the technology. Although project failures are not restricted to CCS (Supplementary Table 5), research shows that the initial hype around the technology during the first wave of industry interest had exacerbated social acceptance beyond typical issues associated with emerging technologies (e.g. NIMBY, infrastructure and storage security), creating a negative feedback loop where project failures decreased social acceptance and thus increased the likelihood of future project failures [25]. Direct support for early demonstration projects in new CCS applications can take the

technology out of the negative feedback loop and enable 'learning from diversity' of CCS applications [57]. This approach suits the current status of CCS, as projects currently in construction and scheduled for operation by 2025 span different CCS applications, among which are hydrogen, DACCS, cement, bioenergy, and power production [58] – as opposed to the 'clean coal' focus in the first wave of industry interest in CCS [57] (Fig. 2). Technology learning across a broader scope of demonstration projects could expedite completion of the formative phase, laying the foundation for reducing both costs and the failure rate that would in turn increase the number of planned projects by stimulating stronger industry interest for 'learning from replication' [57] of successful demonstrations as well as encouraging better socio-political and regulatory conditions by 2030.

Importantly, the replication of CCS plans might require a more nuanced and flexible structure of currently existing and new incentive mechanisms and R&D focus, as currently (e.g. in 45Q) high-purity emitters (e.g. natural gas processing, ethanol) have an advantage over low-purity emitters (e.g. cement, power, steel) when it comes to the cost of capture [59]. Replication will also require the ramp up of infrastructure, and today we see that early-moving projects experience difficulties due to regulatory struggles and opposition from property owners (e.g. Navigator project), pointing at the negative feedback loop indicated above. This challenge (as well as many others) is not unique to CCS (e.g. similar issues were faced by transmission lines for renewables), and the role of policymakers is critical in managing these risks, e.g. by supporting the development of CCS clusters and ensuring stakeholder engagement. Together with international cooperation and knowledge spillovers, successful demonstration via 'diversity-to-replication' could enable various capture technologies to take-off and infrastructure to be installed, leading to increased investments and commitments in the acceleration stage.

Acceleration phase. At the acceleration phase, the policy-priority shifts to creating a market for the technology. In light of our findings, the wider adoption of carbon tax or emission trading systems by 2030 could enable increased revenues from CCS projects which are characteristic of the acceleration phase of growth and have been highlighted as an important factor in defining the likelihood of a CCS project success [26]. Although the impact of these instruments on the likelihood of project success is smaller than e.g. government ownership, this could change once more projects are planned and a higher carbon price is achieved [25]. In this phase, international cooperation could play a big role [57] in driving technology costs down, enabling capital and technology transfers, and creating international CO_2 pipeline networks [52].

Stable growth phase. The uncertainty of CCS growth in the long-term introduces difficulties into making policy prescriptions. However, several points can be drawn from this study and recent literature. First, our analysis shows that most climate pathways envision high growth rates of CCS around mid-century. About one-quarter of these rates are comparable to the global maximum growth of nuclear power and current plans for the near-term growth of renewables in the EU [60]. These episodes of technology growth are motivated (among other factors) by energy supply security concerns, and therefore can be viewed as crisis-driven. As [61] highlight in their analysis of green hydrogen production growth, matching such crisis-driven growth rates would require secure revenue streams via direct investments and public co-financing of projects along the entire value chain. Second, most climate pathways foresee higher carbon prices, and this points to another way of maintaining the long-term growth of CCS. For instance, in thirteen vetted IPCC AR6 pathways that foresee the deployment of CCS and BECCS to meet climate targets (Supplementary Note 6), the cost of carbon in 2050 reaches the median of 138 US$/t (IQR 111-151 US$/t) – in some jurisdictions, stacked revenue streams of CCS projects reach this projected carbon price already today [62]. Therefore the challenge is to replicate this experience in key emitting regions by the mid-century. In addition, successful long-term deployment of CCS can potentially slow down the ambition of actors to achieve rapid decarbonisation via other technologies – in contrast to what is foreseen in the IPCC pathways (Extended Data Fig. 3).

Supplementary Note 5: Inter-ensemble comparison of CCS deployment projections in the IPCC AR5, SR1.5, and AR6

In order to examine whether more recent generations of IPCC scenario ensembles have changed their reliance on CCS for achieving temperature targets, we compare CCS deployment in AR5, SR1.5 and AR6 scenario ensembles. For this inter-ensemble comparison (Supplementary Note 5), we used pathways compatible with the most stringent climate targets (i.e. scenario categories leading to the lowest temperature outcome). The AR5 sample [16] (115 pathways) includes Category 1 (430-480 ppm) which, according to ref. [63], corresponds to "associated 2100 median temperatures ranging from 1.5 to 1.7°C above 1850–1900 levels". The SR1.5 sample [18, 19] (85 pathways) includes "Below 1.5C", "1.5C low

overshoot", and "1.5C high overshoot" – all with the probability of exceeding 1.5°C in 2100 of less than 50%. The AR6 sample [1, 2] (218 pathways) includes Categories 1 ("Below 1.5°C with no or limited overshoot") and 2 ("Below 1.5°C with high overshoot") with a 50% probability of 1.3°C and 1.4°C temperature change by 2100, respectively. In this exercise, it is important to highlight that the most stringent scenario categories in the AR5, SR1.5, and AR6 ensembles are, in fact, not equally stringent: as we explain above, AR6 sample achieves lower temperatures with higher probability by 2100 than AR5. To compare near-term CCS deployment (20 years) in these three scenario ensembles, we aligned them by the time from the year the ensemble was published and used linear interpolation of CCS deployment projections in each pathway to calculate CCS capacity in 2034 for AR5 (2014), 2038 for SR1.5 (2018), and 2042 for AR6 (2022) (Extended Data Fig. 5, Supplementary Table 10).

The three groups of pathways envision similar CCS growth trajectories until stable growth phase – 4.7 Gt/yr (IQR 2.6-6.5) in AR5 and 4.5 Gt/yr (IQR 3.1-8.4) in AR6 are achieved within 20 years since ensemble release (i.e. 2034 for AR5 and 2042 for AR6). However, the maximum growth rate and the maximum eventual capacity of CCS have declined by 29% and 23% respectively between the median of AR5 and the median of AR6 scenarios. The reduction between SR1.5 and AR6 is generally lower, but is particularly noticeable between the two sets of Illustrative Mitigation Pathways (IMPs) – a number of pathways "representing critical mitigation strategies discussed in the assessment" [1]. The first such set of IMPs in SR1.5 [18, 19] had a much bigger role for CCS technologies in reaching the 1.5°C target in all but one mitigation strategy than its descendant – the AR6 set [1, 2] (Extended Data Fig. 5). The only exception is the IMP-LED pathway which does not project any CCS capacity – this pathway was first introduced in SR1.5 and updated in AR6 [64]. Therefore, the IPCC scenarios rely on CCS to a similar extent in the short-term and less in a long-term, although depicting similar deployment trajectories (Supplementary Table 10) – with an overall duration of CCS technology deployment cycle of some 30-50 years.

Supplementary Note 6: Summary of vetted pathways

Using the three feasibility spaces for CCS deployment at the formative, acceleration and stable growth phases, we identify IPCC AR6 pathways with feasible CCS deployment trajectories throughout the century (Methods). Out of 218 1.5°C- and 423 2°C-compatible pathways in the most recent scenario ensemble, we find only three 1.5°C- and eleven 2°C-compatible pathways that are consistent with historical evidence from selected reference cases and current market interest in CCS (Supplementary Table 11). These pathways display two contrasting trajectories for feasible global CCS deployment to meet the Paris target (Supplementary Fig. 7), and another one which does not deploy any CCS.

The first group of pathways (which includes two 1.5°C and three 2°C pathways) displays a deployment trajectory that requires a 108-363 Mt/yr capacity to be implemented at the formative phase by 2030 (0.5-1.8% market share). The lower bound of this range corresponds to a subsector-adjusted failure rate (76%) and no additional plans until 2025, whereas the upper bound would require a 45% failure rate and doubling of current plans by 2025. In the following decade, two 1.5°C pathways envisage acceleration of CCS deployment inline with historical observations for global wind power deployment, whereas two 2°C pathways show acceleration of lower ambition than observed historically for wind, nuclear, or solar power globally. Later on, this group of pathways is characterised by 0.11-0.19 Gt/yr maximum annual capacity additions (0.9-1.3% when normalised to market size at $TMax$) achieved by the mid-century – this is in line with global maximum growth rates of nuclear power and significantly lower than the IQR range for all pathways in their respective temperature groups (Supplementary Table 11), both in absolute values and when normalised to the market size. CCS capacity in this group of pathways saturates by around 2070 at 4-7.5 $GtCO_2$/yr. In this group, reaching 1.5°C (two REMIND pathways) requires fossil CCS saturation by 2040 and BECCS expansion, whereas other negative emission technologies do not play a big role in emission reductions. In contrast, a similar CCS deployment trajectory with no BECCS (POLES "No BECCS" pathway) only leads to 2°C.

The second group of pathways (which includes eight 2°C pathways) displays a deployment trajectory that requires a 47-351 Mt/yr capacity to be implemented at the formative phase by 2030, however with higher maximum growth rates than in the first group – yet still modest (0.16-0.39 Gt/yr) compared to the entire sample (IQR 0.3-0.7 Gt/yr). BECCS and other negative emission technologies play an important role in offsetting CO_2 emissions which, in these pathways, do not decline as rapidly as in pathways in the first group (Supplementary Fig. 8). This results in the late timing of maximum growth achievement and high saturation levels.

Finally, the last group – depicting no CCS deployment – is represented by one pathway (IMP-LED

[64]) which foresees rapid energy demand changes to meet the 1.5°C target without overshoot.

The positions of these pathways relative to the three feasibility spaces at the formative, acceleration, and growth phases are illustrated in Supplementary Fig. 9-11.

Supplementary Note 7: Application of the proposed method to analyse the feasibility of policy-driven technologies' deployment at the regional scale (EU Net Zero Industry Act CCS Target)

Policy-driven technologies are often associated with technology targets and market plans. We show how such plans can be used in conjunction with empirically-derived failure rates to capture erratic deployment patterns of emerging technologies in the formative phase. In this Supplementary Note, we show how this approach can assist in understanding the feasibility of policy targets in the formative and acceleration phases. This Supplementary Note also serves as an example of how this approach can be used at the regional scale.

With the 2030 target proposed in the Net Zero Industry Act (50 Mt/yr [65]) and the 2040 estimate for CCS capacity indicated in the EU Industrial Carbon Management Strategy published in February 2024 (280 Mt/yr [66]), our approach is well-fit to assess the feasibility of near- and medium-term CCS deployment plans in the EU. We do so by constructing feasibility spaces of CCS deployment at the formative (until 2030) and acceleration phases (until 2040) specifically for the European Union. This allows us to, first, identify the feasible range of CCS capacity by 2030 in light of the recently proposed EU NZIA target for CO_2 storage and, second, understand what could be realistically achieved in the following decade provided that the target is met. For the formative phase (until 2030), as in our global analysis, we consider the currently planned capacity (48 Mt) and its doubling by 2025 (which is similar to 80 Mt/yr capture capacity considered plausible by the EU industry [66]), and the same reference cases for failure rates (historical 88%, subsector-adjusted 76%, and nuclear 45%). Lastly, contrasting feasible ranges of CCS capacity to the IPCC pathways allows to align deployment plans with temperature targets. For CCS deployment in scenarios, we use IPCC AR6 pathways for R10 regions that divide the globe into ten regions. Note that NZIA target only covers EU states, whereas IPCC R10EUROPE region includes the UK and some other non-EU countries.

In this decade, we see that the achievement of the proposed 50 Mt/yr target would lead to CCS capacity higher than the 2°C median by 2030 (38 Mt/yr), provided that this storage is utilised by the end of the decade. Currently planned 2030 capacity stands at 48 Mt, therefore reaching the NZIA target will require improvements in failure rates beyond the historical (88%) and subsector-adjusted (76%), even if planned capacity doubles by 2025 (Extended Data Fig. 6). The resulting feasibility range spans CCS capacity in the EU in 2030 from 8 to 55 Mt/yr, or 0.5-3.4% of today's capturable emissions (ca. 1625 Mt/yr [67]). We find only a few 1.5°C pathways that are inline with this range.

For the feasibility space at the acceleration phase of CCS deployment in the EU, we use reference cases of wind [68], solar [69] and nuclear [70] power acceleration in the EU as well as wind power acceleration in China and nuclear power acceleration in the US starting from the same level of market penetration that can be achieved at the formative phase (8-55 Mt/yr or 0.5-3.4%), which includes the NZIA target (50 Mt/yr, blue vertical line in Extended Data Fig. 7).

Meeting the NZIA CO_2 storage target would be in line with reaching a range of 2°C pathways provided that this storage is utilised by the end of the decade. What does it mean for the 2040 target? Staying on the 2°C trajectory (251 Mt/yr median) will require annual growth in the acceleration phase of around 17-18% in 2030-2040 (Extended Data Fig. 7, highest density zone in the 2D-density plot), which would be comparable to the historical acceleration of wind and nuclear power in the EU and would lead to the operational CCS capacity of ca. 280 Mt/yr by 2040. This is in line with the 2040 CCS estimate provided in the EU Industrial Carbon Management Strategy (280 Mt/yr). Getting CCS on track for 1.5°C target (437 Mt/yr median) in 2030-2040 would require more optimistic annual growth around 25% which was observed for wind power in China and nuclear power in the US (Extended Data Table 3).

References

[1] IPCC. Climate Change 2022: Mitigation of Climate Change. Contribution of Working Group III to the Sixth Assessment Report of the Intergovernmental Panel on Climate Change [P.R. Shukla, J. Skea, R. Slade, A. Al Khourdajie, R. van Diemen, D. McCollum, M. Pathak, S. Some, P. Vyas, R. Fradera, M. Belkacemi, A. Hasija, G. Lisboa, S. Luz, J. Malley, (eds.)] (2022).

[2] Byers, E. *et al.* AR6 Scenarios Database (2022). URL https://doi.org/10.5281/zenodo.5886912.

[3] Nemet, G., Greene, J., Müller-Hansen, F. & Minx, J. C. Dataset on the adoption of historical technologies informs the scale-up of emerging carbon dioxide removal measures. *Communications Earth & Environment* **4**, 397 (2023).

[4] Kazlou, T., Cherp, A. & Jewell, J. Code and data for the article "Feasible deployment of carbon capture and storage and the requirements of climate targets" (2024). URL https://doi.org/10.5281/zenodo.12706872.

[5] S&P Global Platts. World Electric Power Plants Data Base (WEPP) (2013).

[6] S&P Global Platts. World Electric Power Plants Data Base (WEPP) (2022).

[7] EIA. Petra Nova is one of two carbon capture and sequestration power plants in the world - U.S. Energy Information Administration (EIA) (2017). URL https://www.eia.gov/todayinenergy/detail.php?id=33552.

[8] Bellona. Norway's Longship CCS project (2020). URL https://network.bellona.org/content/uploads/sites/3/2020/10/Longship-Briefing_Bellona-1.pdf.

[9] IEA Bioenergy. Deployment of BECCUS value chains in the United States (2023). URL https://www.ieabioenergy.com/wp-content/uploads/2023/03/BECCUS-1.0_US-Case-Study_final_update.pdf.

[10] CarbonCredits. BlackRock Places $550M Bet on Occidental's DAC Project STRATOS (2023). URL https://carboncredits.com/blackrock-places-550m-bet-on-occidentals-dac-project-stratos/.

[11] Grubler, A. The costs of the French nuclear scale-up: A case of negative learning by doing. *Energy Policy* **38**, 5174–5188 (2010).

[12] NREL. IEA Wind Task 26: The Past And Future Cost Of Wind Energy (2012). URL https://www.nrel.gov/docs/fy12osti/53510.pdf.

[13] IRENA. Solar Photovoltaics (2012). URL https://www.irena.org/-/media/Files/IRENA/Agency/Publication/2012/RE_Technologies_Cost_Analysis-SOLAR_PV.pdf.

[14] Rubin, E. S., Yeh, S., Antes, M., Berkenpas, M. & Davison, J. Use of experience curves to estimate the future cost of power plants with CO2 capture. *International Journal of Greenhouse Gas Control* **1**, 188–197 (2007).

[15] U.S. Official Inflation Data, Alioth Finance. Inflation Calculator. (2024). URL https://www.officialdata.org/.

[16] IPCC. Climate Change 2014: Mitigation of Climate Change. Contribution of Working Group III to the Fifth Assessment Report of the Intergovernmental Panel on Climate Change. Tech. Rep., Cambridge University Press, Cambridge, United Kingdom and New York, NY, USA (2014).

[17] Rogelj, J. *et al.* Mitigation Pathways Compatible with 1.5°C in the Context of Sustainable Development (2018). URL http://www.ipcc.ch/report/sr15/.

[18] IPCC. Global warming of 1.5°C. An IPCC Special Report on the impacts of global warming of 1.5°C above pre-industrial levels and related global greenhouse gas emission pathways, in the context of strengthening the global response to the threat of climate change, sustainable development, and efforts to eradicate poverty [V. Masson-Delmotte, P. Zhai, H. O. Pörtner, D. Roberts, J. Skea, P.R. Shukla, A. Pirani, W. Moufouma-Okia, C. Péan, R. Pidcock, S. Connors, J. B. R. Matthews, Y. Chen, X. Zhou, M. I. Gomis, E. Lonnoy, T. Maycock, M. Tignor, T. Waterfield (eds.)]. (2018).

[19] Huppmann, D. *et al.* IAMC 1.5°C Scenario Explorer and Data hosted by IIASA (2019). URL https://zenodo.org/record/3363345.

[20] GCCSI. CO2RE Database. URL https://co2re.co/.

[21] MIT. Carbon Capture and Sequestration Technology Program Database. URL https://sequestration.mit.edu/.

[22] NETL. Worldwide CCS Database. URL https://netl.doe.gov/coal/carbon-storage/worldwide-ccs-database.

[23] Scottish CCS. Global CCS Map. URL https://www.sccs.org.uk/expertise/global-ccs-map.

[24] IEA. CCUS Projects Explorer (2023). URL https://www.iea.org/data-and-statistics/data-tools/ccus-projects-explorer.

[25] Wang, N., Akimoto, K. & Nemet, G. F. What went wrong? Learning from three decades of carbon capture, utilization and sequestration (CCUS) pilot and demonstration projects. *Energy Policy* **158**, 112546 (2021).

[26] Abdulla, A., Hanna, R., Schell, K. R., Babacan, O. & Victor, D. G. Explaining successful and failed investments in U.S. carbon capture and storage using empirical and expert assessments. *Environmental Research Letters* **16**, 014036 (2021).

[27] Azarova, V., Cohen, J., Friedl, C. & Reichl, J. Designing local renewable energy communities to increase social acceptance: Evidence from a choice experiment in Austria, Germany, Italy, and Switzerland. *Energy Policy* **132**, 1176–1183 (2019).

[28] Susskind, L. *et al.* Sources of opposition to renewable energy projects in the United States. *Energy Policy* **165**, 112922 (2022).

[29] Donald, J., Axsen, J., Shaw, K. & Robertson, B. Sun, wind or water? Public support for large-scale renewable energy development in Canada. *Journal of Environmental Policy & Planning* **24**, 175–193 (2022).

[30] Hall, N., Ashworth, P. & Devine-Wright, P. Societal acceptance of wind farms: Analysis of four common themes across Australian case studies. *Energy Policy* **58**, 200–208 (2013).

[31] Jones, C. R. & Eiser, J. R. Understanding 'local' opposition to wind development in the UK: How big is a backyard? *Energy Policy* **38**, 3106–3117 (2010).

[32] Pasqualetti, M. J. Social barriers to renewable energy landscapes. *Geographical Review* **101**, 201–223 (2011).

[33] Saputra, B., Fajri, H., Akmal, A. D., Wahyuni, N. & Halawa, H. S. Agree or disagree: local youth's perception of renewable energy development. *IOP Conference Series: Earth and Environmental Science* **896**, 012038 (2021).

[34] Yuan, X., Zuo, J. & Ma, C. Social acceptance of solar energy technologies in China—End users' perspective. *Energy Policy* **39**, 1031–1036 (2011).

[35] Ewijk, S. v. & McDowall, W. Diffusion of flue gas desulfurization reveals barriers and opportunities for carbon capture and storage. *Nature Communications* **11**, 4298 (2020).

[36] Malhotra, A. & Schmidt, T. S. Accelerating Low-Carbon Innovation. *Joule* (2020).

[37] Wilson, C. *et al.* Granular technologies to accelerate decarbonization. *Science* **368**, 36–39 (2020).

[38] Kearns, D., Liu, H. & Consoli, C. *Technology Readiness and costs of CCS* (Global CCS Institute, 2021).

[39] Rubin, E. S., Davison, J. E. & Herzog, H. J. The cost of CO2 capture and storage. *International Journal of Greenhouse Gas Control* **40**, 378–400 (2015).

[40] Irlam, L. GLOBAL COSTS OF CARBON CAPTURE AND STORAGE (2017).

[41] IEA. Is carbon capture too expensive? (2021). URL https://www.iea.org/commentaries/is-carbon-capture-too-expensive.

[42] Laude, A., Ricci, O., Bureau, G., Royer-Adnot, J. & Fabbri, A. CO2 capture and storage from a bioethanol plant: Carbon and energy footprint and economic assessment. *International Journal of Greenhouse Gas Control* **5**, 1220–1231 (2011).

[43] Cherp, A., Vinichenko, V., Tosun, J., Gordon, J. A. & Jewell, J. National growth dynamics of wind and solar power compared to the growth required for global climate targets. *Nature Energy* **6**, 742–754 (2021).

[44] Breetz, H., Mildenberger, M. & Stokes, L. The political logics of clean energy transitions. *Business and Politics* **20**, 492–522 (2018).

[45] Merk, C., Nordo, A. D., Andersen, G., Lægreid, O. M. & Tvinnereim, E. Don't send us your waste gases: Public attitudes toward international carbon dioxide transportation and storage in Europe. *Energy Research & Social Science* **87**, 102450 (2022).

[46] Egmond, S. v. & Hekkert, M. P. Analysis of a prominent carbon storage project failure – The role of the national government as initiator and decision maker in the Barendrecht case. *International Journal of Greenhouse Gas Control* **34**, 1–11 (2015).

[47] Taylor, M. R., Rubin, E. S. & Hounshell, D. A. Control of SO2 emissions from power plants: A case of induced technological innovation in the U.S. *Technological Forecasting and Social Change* **72**, 697–718 (2005).

[48] Taylor, M. *The Influence of Government Actions on Innovative Activities in the Development of Environmental Technologies to Control Sulfur Dioxide Emissions from Stationary Sources.* Ph.D. thesis, Carnegie Institute of Technology (2001).

[49] BNEF. Carbon Capture Investment Hits Record High of $6.4 Billion (2023). URL `https://about.bnef.com/blog/carbon-capture-investment-hits-record-high-of-6-4-billion/`.

[50] Flyvbjerg, B. & Gardner, D. *How Big Things Get Done: The Surprising Factors That Determine the Fate of Every Project, from Home Renovations to Space Exploration and Everything In Between* (McClelland & Stewart, 2023).

[51] Merrow, E. W. Industrial Megaprojects: Concepts, strategies and practices for success (2011).

[52] Wei, Y.-M. *et al.* A proposed global layout of carbon capture and storage in line with a 2 °C climate target. *Nature Climate Change* **11**, 112–118 (2021).

[53] IEA. Energy Technology Perspectives 2020: Special Report on Carbon Capture, Utilisation and Storage (2020). URL `https://iea.blob.core.windows.net/assets/181b48b4-323f-454d-96fb-0bb1889d96a9/CCUS_in_clean_energy_transitions.pdf`.

[54] McCollum, D. L. *et al.* Energy investment needs for fulfilling the Paris Agreement and achieving the Sustainable Development Goals. *Nature Energy* **3**, 589–599 (2018).

[55] IEA. World Energy Investment 2023 (2023). URL `https://iea.blob.core.windows.net/assets/8834d3af-af60-4df0-9643-72e2684f7221/WorldEnergyInvestment2023.pdf`.

[56] European Commission. COMMUNICATION FROM THE COMMISSION TO THE EUROPEAN PARLIAMENT, THE COUNCIL, THE EUROPEAN ECONOMIC AND SOCIAL COMMITTEE AND THE COMMITTEE OF THE REGIONS A hydrogen strategy for a climate-neutral Europe (2020). URL `https://eur-lex.europa.eu/legal-content/EN/TXT/?uri=CELEX:52020DC0301`.

[57] Reiner, D. M. Learning through a portfolio of carbon capture and storage demonstration projects. *Nature Energy* **1**, 15011 (2016).

[58] GCCSI. Global Status of CCS 2022: Global CCS Institute Report. (2022). URL `https://status22.globalccsinstitute.com/wp-content/uploads/2022/10/Global-Status-of-CCS-2022-Report-Final-compressed.pdf`.

[59] Rode, D. C., Anderson, J. J., Zhai, H. & Fischbeck, P. S. Six principles to guide large-scale carbon capture and storage development. *Energy Research & Social Science* **103**, 103214 (2023).

[60] Vinichenko, V., Jewell, J., Jacobsson, J. & Cherp, A. Historical diffusion of nuclear, wind and solar power in different national contexts: implications for climate mitigation pathways. *Environmental Research Letters* **18** (2023).

[61] Odenweller, A., Ueckerdt, F., Nemet, G. F., Jensterle, M. & Luderer, G. Probabilistic feasibility space of scaling up green hydrogen supply. *Nature Energy* 1–12 (2022).

[62] GCCSI. The Investment Case for CCS: Policy Drive and Case Studies. URL `https://www.globalccsinstitute.com/wp-content/uploads/2023/09/The-Investment-Case-for-CCS-Global-CCS-Institute.pdf`.

[63] Kikstra, J. S. *et al.* The IPCC Sixth Assessment Report WGIII climate assessment of mitigation pathways: from emissions to global temperatures. *Geoscientific Model Development* **15**, 9075–9109 (2022).

[64] Grubler, A. *et al.* A low energy demand scenario for meeting the 1.5 °C target and sustainable development goals without negative emission technologies. *Nature Energy* **3**, 515–527 (2018).

[65] European Commission. Proposal for a Regulation of the European Parliament and of the Council on establishing a framework of measures for strengthening Europe's net-zero technology products manufacturing ecosystem (Net Zero Industry Act) (2023). URL `https://eur-lex.europa.eu/legal-content/EN/ALL/?uri=CELEX:52023PC0161`.

[66] European Commission. COMMUNICATION FROM THE COMMISSION TO THE EUROPEAN PARLIAMENT, THE COUNCIL, THE EUROPEAN ECONOMIC AND SOCIAL COMMITTEE AND THE COMMITTEE OF THE REGIONS. Towards an ambitious Industrial Carbon Management for the EU (2024). URL `https://eur-lex.europa.eu/legal-content/EN/TXT/PDF/?uri=CELEX:52024DC0062`.

[67] Minx, J. C. *et al.* A comprehensive and synthetic dataset for global, regional and national greenhouse gas emissions by sector 1970-2018 with an extension to 2019 (2022). URL `https://zenodo.org/record/6483002/export/hx`.

[68] IEA. World Energy Statistics and Balances (database) (2022).

[69] EMBER. Yearly electricity data. Access Date: 02.08.2023. URL `https://ember-climate.org/data-catalogue/yearly-electricity-data/`.

[70] UNSD. Energy Statistics Database (2013).
