# Supplementary material for: Feasible deployment of carbon capture and storage and the requirements of climate targets
Source: Nat Clim Chang. 2024 Sep 25;14(10):1047–55. doi: 10.1038/s41558-024-02104-0 (PMC11458486; doi:10.1038/s41558-024-02104-0)
Supplement: Supplementary file 2 — Reporting Summary [file 41558_2024_2104_MOESM2_ESM.pdf]

nature portfolio

Corresponding author(s): Jessica Jewell

Last updated by author(s): Jul 16, 2024

Reporting Summary

Nature Portfolio wishes to improve the reproducibility of the work that we publish. This form provides structure for consistency and transparency in reporting. For further information on Nature Portfolio policies, see our Editorial Policies and the Editorial Policy Checklist.

Statistics

For all statistical analyses, confirm that the following items are present in the figure legend, table legend, main text, or Methods section.

n/a	Confirmed	
☐	☒	The exact sample size (*n*) for each experimental group/condition, given as a discrete number and unit of measurement
☒	☐	A statement on whether measurements were taken from distinct samples or whether the same sample was measured repeatedly
☒	☐	The statistical test(s) used AND whether they are one- or two-sided<br>*Only common tests should be described solely by name; describe more complex techniques in the Methods section.*
☒	☐	A description of all covariates tested
☒	☐	A description of any assumptions or corrections, such as tests of normality and adjustment for multiple comparisons
☐	☒	A full description of the statistical parameters including central tendency (e.g. means) or other basic estimates (e.g. regression coefficient) AND variation (e.g. standard deviation) or associated estimates of uncertainty (e.g. confidence intervals)
☒	☐	For null hypothesis testing, the test statistic (e.g. *F*, *t*, *r*) with confidence intervals, effect sizes, degrees of freedom and *P* value noted<br>*Give P values as exact values whenever suitable.*
☒	☐	For Bayesian analysis, information on the choice of priors and Markov chain Monte Carlo settings
☒	☐	For hierarchical and complex designs, identification of the appropriate level for tests and full reporting of outcomes
☒	☐	Estimates of effect sizes (e.g. Cohen's *d*, Pearson's *r*), indicating how they were calculated

Our web collection on statistics for biologists contains articles on many of the points above.

Software and code

Policy information about availability of computer code

Data collection	No software was used for data collection
Data analysis	This study used R programming language (v. 4.3.2 2023-10-31) for data analysis.

For manuscripts utilizing custom algorithms or software that are central to the research but not yet described in published literature, software must be made available to editors and reviewers. We strongly encourage code deposition in a community repository (e.g. GitHub). See the Nature Portfolio guidelines for submitting code & software for further information.

Data

Policy information about availability of data

All manuscripts must include a data availability statement. This statement should provide the following information, where applicable:

- Accession codes, unique identifiers, or web links for publicly available datasets
- A description of any restrictions on data availability
- For clinical datasets or third party data, please ensure that the statement adheres to our policy

The data for this Analysis, including the dataset of historical and planned CCS projects, are available via Zenodo at https://doi.org/10.5281/zenodo.12706872 and GitHub at https://github.com/poletresearch/CCS_article. For our analysis of CCS deployment in climate-constrained scenarios, we use three most recent IPCC scenario ensembles: AR5 (https://iiasa.ac.at/models-tools-data/ar5), SR1.5 (https://data.ene.iiasa.ac.at/iamc-1.5c-explorer/#/login?redirect=%2Fworkspaces), and AR6 (https://iiasa.ac.at/models-tools-data/ar6-scenario-explorer-and-database). For the historical acceleration of wind and stable growth rates of wind and solar

electricity production, we use IEA World Energy Balances (https://www.iea.org/data-and-statistics/data-product/world-energy-balances). For historical acceleration of solar, we use EMBER Yearly electricity data (https://ember-climate.org/data-catalogue/yearly-electricity-data/). For the historical growth of nuclear, we use UNSD Energy Statistics Database (https://unstats.un.org/unsd/energystats/).

Human research participants

Policy information about studies involving human research participants and Sex and Gender in Research.

Reporting on sex and gender	N/A
Population characteristics	N/A
Recruitment	N/A
Ethics oversight	N/A

Note that full information on the approval of the study protocol must also be provided in the manuscript.

Field-specific reporting

Please select the one below that is the best fit for your research. If you are not sure, read the appropriate sections before making your selection.

☐ Life sciences ☒ Behavioural & social sciences ☐ Ecological, evolutionary & environmental sciences

For a reference copy of the document with all sections, see nature.com/documents/nr-reporting-summary-flat.pdf

Behavioural & social sciences study design

All studies must disclose on these points even when the disclosure is negative.

Study description	The study projects feasible ranges of carbon capture and storage (CCS) deployment in this century based on historical evidence of CCS deployment and other policy-driven technologies.
Research sample	The study uses quantitative data of CCS industry plans, historical growth of reference cases, and CCS growth in the IPCC pathways.
Sampling strategy	For CCS industry plans, we built a dataset of completed, failed, and currently planned commercial (at least 0.1 Mt/yr capacity) CCS projects starting from 1972, the completion year of the first integrated CCS project (Terrell natural gas processing plant). For analysing future trajectories of CCS deployment in the IPCC AR6 scenario ensemble, we used pathways classified under IPCC AR6 Scenario Categories 1 ("Below 1.5°C with no or limited overshoot") and 2 ("Below 1.5°C with high overshoot"), 3 ("Likely below 2°C"), 4 ("Below 2°C"), and 5 ("Below 2.5°C"). We further grouped these categories into 1.5°C- (Category 1 and 2), 2°C- (Category 3 and 4), and 2.5°C -(Category 5) compatible pathways For the historical growth of reference cases, we used datasets described in the Data Availability statement without any corrections.
Data collection	Data on completed and failed projects has been collected from annual Global CCS Institute (GCCSI) reports as well as dormant and existing databases listing planned CCS projects at different points in time (full list in Supplementary Table 2), whereas data on currently planned projects has been gathered primarily from the recently published (March 2023) IEA CCUS Projects Database. These sources have been complemented with a systematic Google search described in the Supplementary Note 1.
Timing	The systematic Google search for CCS industry plans was conducted in November 2021 - March 2022 (Methods, Supplementary Note 1).
Data exclusions	Since our analysis compares future deployment trajectories for different temperature targets, we excluded pathways made by model families that did not produce scenarios for each of the three temperature groups (described above) in the IPCC AR6 scenario ensemble: TIAM (40 scenarios), C-ROADS (5), EPPA (5), and MERGE (1). We also excluded 30 scenarios where CCS capacity in 2020 was reported to be more than 50 MtCO2 (which is not representative of the actual CCS capacity in 2023). Our final sample was 840 scenarios: 218 1.5°C-, 423 2°C-, 199 2.5°C-compatible scenarios. The resulting sample was harmonized to represent the combined capacity of BECCS, DACCS, and CCS technologies for our further analysis.
Non-participation	N/A
Randomization	N/A

Reporting for specific materials, systems and methods

We require information from authors about some types of materials, experimental systems and methods used in many studies. Here, indicate whether each material, system or method listed is relevant to your study. If you are not sure if a list item applies to your research, read the appropriate section before selecting a response.

Materials & experimental systems

n/a	Involved in the study
☒	☐ Antibodies
☒	☐ Eukaryotic cell lines
☒	☐ Palaeontology and archaeology
☒	☐ Animals and other organisms
☒	☐ Clinical data
☒	☐ Dual use research of concern

Methods

n/a	Involved in the study
☒	☐ ChIP-seq
☒	☐ Flow cytometry
☒	☐ MRI-based neuroimaging
